# Discovery of Selective Inhibitor Leads by Targeting an Allosteric Site in Insulin-Regulated Aminopeptidase

**DOI:** 10.3390/ph14060584

**Published:** 2021-06-18

**Authors:** Ioannis Temponeras, Lykourgos Chiniadis, Athanasios Papakyriakou, Efstratios Stratikos

**Affiliations:** 1 National Centre for Scientific Research Demokritos, Agia Paraskevi, Attikis, 15341 Athens, Greece; john.t13111@windowslive.com (I.T.); lchiniadis@gmail.com (L.C.); 2 Laboratory of Biochemistry, Department of Chemistry, National and Kapodistrian University of Athens, Panepistimioupolis Zografou, 15771 Athens, Greece

**Keywords:** aminopeptidase, peptides, lead-like inhibitors, docking, molecular dynamics, free-energy calculations, enzymatic assays, allosteric, cognitive enhancement, immune system

## Abstract

Insulin-Regulated aminopeptidase (IRAP) is a zinc-dependent aminopeptidase with several important biological functions and is an emerging pharmaceutical target for cognitive enhancement and immune system regulation. Aiming to discover lead-like IRAP inhibitors with enhanced selectivity versus homologous enzymes, we targeted an allosteric site at the C-terminal domain pocket of IRAP. We compiled a library of 2.5 million commercially available compounds from the ZINC database, and performed molecular docking at the target pocket of IRAP and the corresponding pocket of the homologous endoplasmic reticulum aminopeptidase 1 (ERAP1). Of the top compounds that showed high selectivity, 305 were further analyzed by molecular dynamics simulations and free energy calculations, leading to the selection of 33 compounds for in vitro evaluation. Two orthogonal functional assays were employed: one using a small fluorogenic substrate and one following the degradation of oxytocin, a natural peptidic substrate of IRAP. In vitro evaluation suggested that several of the compounds tested can inhibit IRAP, but the inhibition profile was dependent on substrate size, consistent with the allosteric nature of the targeted site. Overall, our results describe several novel leads as IRAP inhibitors and suggest that the C-terminal domain pocket of IRAP is a promising target for developing highly selective IRAP inhibitors.

## 1. Introduction

Insulin-Regulated Aminopeptidase (IRAP, EC 3.4.11.3) is a transmembrane zinc metalloprotease that has been implicated in several important biological functions ranging from roles in glucose metabolism, cognitive functions and the immune system [1,2,3,4]. Currently, IRAP is under investigation as a pharmaceutical target for cognitive disorders [5]. Although several IRAP inhibitors have been developed [6,7,8,9] no clinical applications have yet been reported. Possible reasons include limited selectivity, limited pharmacodynamics or deficient pharmacokinetics. Up to now, most efforts on developing IRAP inhibitors have either targeted the active site or utilized random library screening [10,11,12]. However, the IRAP active site is highly homologous to other aminopeptidases of the M1 family and is characterized by structural plasticity that could complicate the development of potent and selective inhibitors [13]. As a result, targeting other sites in IRAP could constitute a viable strategy that would allow the discovery of novel leads that could be developed to inhibitors with superior selectivity and pharmacodynamics. Indeed, in a recent study, a spiro-oxindole dihydroquinazolinone derivative was demonstrated to be an un-competitive inhibitor of IRAP trimming a small dipeptide substrate, although the exact mechanism of inhibition is yet to be determined [12].

**Figure 1:**
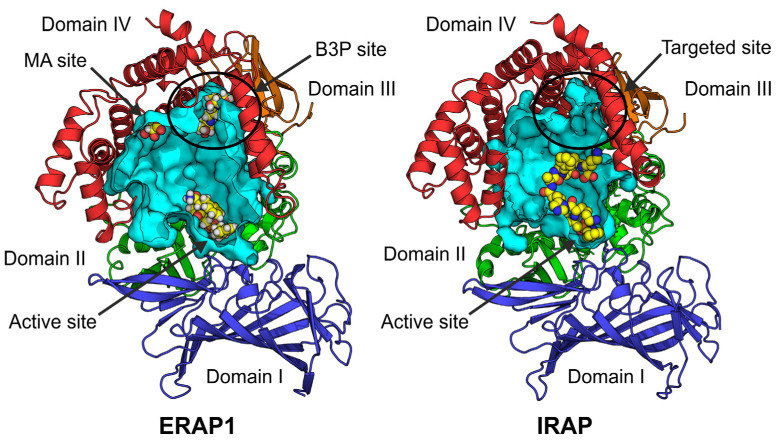
Schematic representations of the internal cavity or ERAP1 (**left**) and IRAP (**right**). Figures were constructed based on the coordinates of PDB ID: 6q4r and 4z7i, respectively, retrieved from RCSB Protein Data Bank [17]. Internal cavities are shown in cyan surface representation and proteins in cartoon representations are color-coded by domain. Ligands found in each cavity are shown in sphere representation (C: yellow, N: blue, O: red, H: white). The active sites accommodating an inhibitor for ERAP1 and a peptide analogue for IRAP are indicated. The sites where a bis-tris propane (B3P) and a malic acid (MA) molecule were found in ERAP1 are indicated, as well as the equivalent site of IRAP that was targeted for virtual ligand screening.

The IRAP crystal structure has revealed a large internal cavity, sufficient in size to accommodate large peptidic substrates, adjacent to the catalytic site [14]. An additional crystal structure of a complex between a pseudopeptide inhibitor and IRAP revealed a distinct conformation in which the enzyme adopts a closed structure and the internal cavity has no direct access to the outside solvent [13]. A structure with similar configuration, solved at 1.7 Å resolution, has been reported for the homologous aminopeptidase ER aminopeptidase 1 (ERAP1), an enzyme important for the adaptive immune response [15]. In that structure, a similar internal cavity in ERAP1 was found to feature small MW buffer components, a malic acid (MA) and a bis-tris-propane molecule (B3P) bound onto two distinct allosteric sites (Figure 1). The site accommodating the malic acid was later discovered to be the site of an allosteric inhibitor of ERAP1 [16]. Inspired by that finding, we decided to focus on the allosteric site in IRAP that is equivalent to the bis-tris-propane site in ERAP1, in order to discover allosteric inhibitors with enhanced selectivity (Figure 1).

## 2. Results

### 2.1. Selection of a Lead-Like Dataset

**Figure 2:**
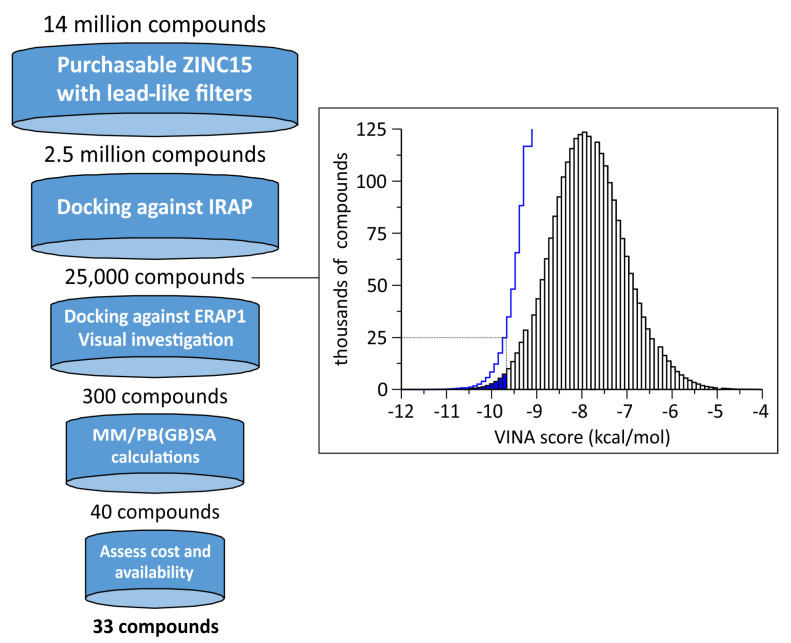
Virtual screening strategy employed for the discovery of lead-like allosteric inhibitors of IRAP, indicating the approximate number of compounds used in each stage. Inset, the distribution of number of compounds (in thousands) as a function of estimated free energy of binding (VINA score). The blue line shows the cumulative distribution and the highlighted region indicates the top 1% (lowest energy) of the compounds selected.

With the aim to discover compounds that bind at the corresponding pocket of IRAP and inhibit its enzymatic activity for natural substrates, we carried out a multi-step virtual screening strategy (Figure 2). First, we obtained a lead-like compilation of compounds from the ZINC15 database [18] by applying commercial availability, reactivity and physiochemical properties filters. The ZINC15 chemical space comprises more than 1.4 billion of annotated substances, of which approximately 14 million 3D protomers (in the pH range of 6.4–8.4) are readily available for purchase from commercial vendors (lead time of 2 weeks) and display standard reactivity (including mildly reactive electrophilic or nucleophilic groups, http://wiki.docking.org/index.php/Reactivity_axis, accessed on 3 June 2021). From this chemical space, we selected a subset of lead-like compounds that was slightly modified from the predefined ZINC15 “Lead-Like” subset (Figure S1). In particular, a 2.5-million subset was obtained using the following filters: (i) in-stock availability; (ii) reference pH of 7.4; (iii) free from reactive groups or PAINS patterns (anodyne subset) [19]; (iv) MW between 200 and 350; (v) calculated logP (octanol-water partition coefficient) below 3.0. The lower MW and higher hydrophobicity cut-offs were applied so as to maximize the potential aqueous solubility of the selected compounds (the preset “Lead-Like” subset contains substances with MW > 250 and logP < 3.5). The anodyne filter was chosen in order to avoid compounds with reactive groups or assay-interfering patterns, including PAINS, which could react with the enzyme or interfere with the assays employed (vide infra).

### 2.2. Virtual Screening of the Compounds

From the selected lead-like subset, a total of ca. 2.45 million compounds were finally obtained in AutoDock PDBQT format [20] and were employed in docking to IRAP using AutoDock VINA [21]. The highest resolution structure of IRAP resolved at 2.5 Å was employed (PDB ID: 5mj6) [13] and the search space was centered within the targeted pocket (see Methods 4.1). The resulting conformers were ranked according to the estimated binding affinity, as given by the VINA score, and from the top-ranked solutions we selected ca. 25,000 compounds for further evaluation (top-1%, Figure 2). In order to maximize our chances of obtaining IRAP-selective inhibitors, we performed a second round of docking calculations against the corresponding site of ERAP1, the highest homologous enzyme of IRAP within the M1 family of aminopeptidases [22]. The top-ranked subset of compounds identified for IRAP was then employed in docking to ERAP1, using the high-resolution X-ray structure (PDB ID: 6q4r [15]). The resulting VINA scores for ERAP1 were then used to rank the compounds according to their difference in their binding affinities with respect to IRAP, so as to disfavor compounds with high probability to bind ERAP1. In this way, we narrowed down the top-ranked subset to 423 compounds that displayed either binding scores below −10.9 kcal/mol for IRAP, or a difference in the estimated free energy of binding with respect to ERAP1 below −2.5 kcal/mol (Tables S1 and S2, respectively).

### 2.3. Free Energy Calculations

Considering that AutoDock VINA has achieved a standard error of 2.85 kcal/mol between predicted and experimental free energies of binding [21], and that our selection of compounds displays a range of scores within 2 kcal/mol (between −11.7 and −9.7), we sought to employ a more accurate method for the determination of their relative binding affinity for IRAP. For this reason, we selected MM/PB(GB)SA, a methodology that combines molecular mechanics energies with the Poisson–Boltzmann (or generalized Born) methods and the surface area continuum solvation methods [23]. Although this method is not as accurate for the determination of absolute binding free energies as are more rigorous free energy methods (such as the free energy perturbation and thermodynamic integration methods), MM/PB(GB)SA is very popular and efficient for the purpose of virtual screening [24]. In particular, we employed the single-trajectory approach by extracting the ensemble average of the free receptor and ligand from a single simulation of the complex. We also omitted calculation of the entropic term, as it has been shown to have minimal effect in comparative results from large sets in virtual screening [25].

**Figure 3:**
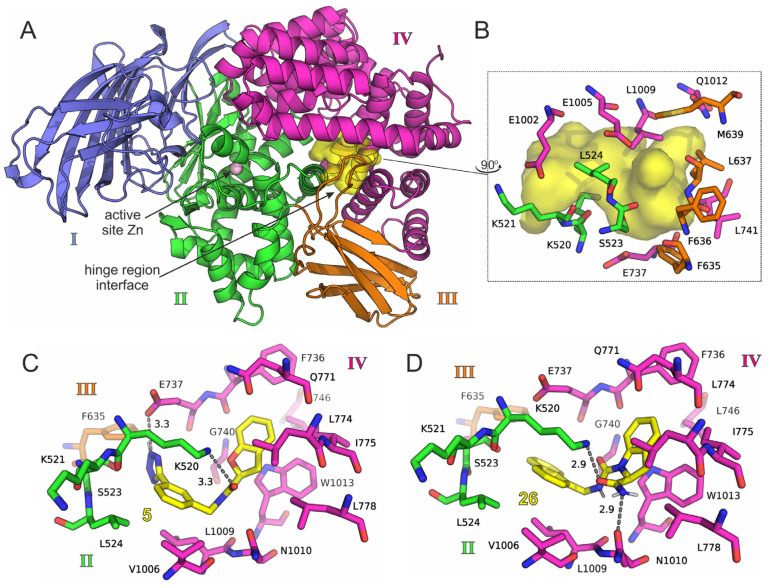
(**A**) Crystal structure of IRAP illustrating the position of targeted pocket and color-coded according to its functional domain I–IV. (**B**) Key residues that comprise the targeted pocket, color-coded according to IRAP domain. (**C**,**D**) Representative docked poses of two compounds, **5** and **26**, shown in yellow-C sticks. Hydrogen bonding interactions with key residues from domains II and IV of IRAP are indicated with dashed lines and the corresponding distance in Å.

Before setting up the MD simulations for the free energy calculations, we performed an additional step comprising visual investigation of the predicted bound conformations for the 423 selected IRAP–ligand complexes, so as to discard compounds that did not display potential for interactions with key residues, or hydrogen-bonding interactions (Figure 3). Specifically, we favored compounds with the potential for interactions with Lys520 from domain II, Phe635 from domain III and the pocket comprising mainly of the hydrophobic residues Phe736, Leu774, Leu1009 and Trp1013 from domain IV. In this way, we selected a final set of 305 compounds for further evaluation with MM/PB(GB)SA calculations, which displayed the most favorable interactions at the hinge domain of IRAP and at the same time had specific interactions with residues of domains II and IV. Each complex was simulated for 20 ns of unbiased MD run in explicit solvent, for an aggregate of > 6 μs of simulation time (see Methods 4.2). From the second half of each simulation 1000 equally sampled snapshots were extracted for the free energy calculations using 2 parameter sets of MM/GBSA and 2 sets of MM/PBSA calculations for comparison (see Methods 4.3). The full set of results from the free energy calculations, including energy decomposition, are given in Supplementary Material (Tables S3–S6).

### 2.4. Final Selection of the Compounds for Evaluation

The final assessment for the selection of compounds for experimental screening was mainly based on the free energy results from the MM/PBSA calculations. As a result, 32 compounds were selected among the top-30 ranked results obtained using the two MM/PBSA methods (Table S7). In addition, we selected compounds that displayed highly ranked free energy of binding in MM/GBSA calculations, but also taking into consideration their ranking from MM/PBSA results. This way, an additional set of 8 compounds was selected (**1**, **7**, **12**, **15**, **24**, **28**, **29** and **33** in Table 1). From the final set of 40 unique compounds, 33 were finally obtained from MolPort after considering their availability and cost (Figures S2 and S3). Compounds were used without further purification and as mixtures of enantiomers or diastereomers, where applicable (Table S7). A complete set of their physiochemical properties was calculated using SwissADME server [26] and is given in Figure S4 and Table S8.

### 2.5. In Vitro Screening using a Small Fluorigenic Peptide

**Figure 4:**
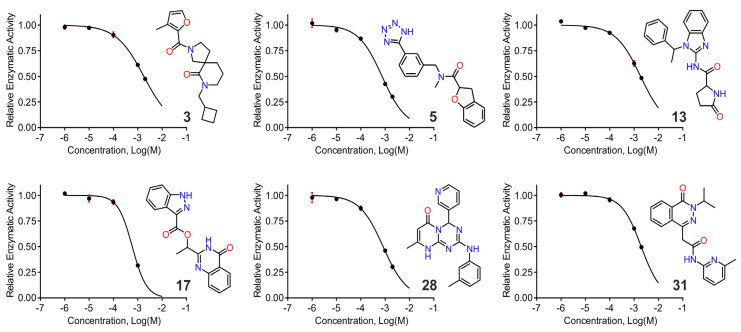
Representative titrations showing the dose-dependent decrease in hydrolysis of the small substrate Leu-AMC by IRAP upon addition of compounds. The structure of the corresponding compound is indicated in each panel and error bars indicate standard deviation that is only shown if significantly larger than the data point size. Solid lines represent fits to a variable-slope inhibitor vs response model using Graphpad prism™.

To evaluate the inhibitory activity of the selected compounds, we first employed a commonly used kinetic fluorogenic assay that follows hydrolysis of l-Leucine-7-amino-4-methylcoumarin [28]. The assay was validated with two previously described IRAP inhibitors, namely DG013A, a pseudopeptide transition state analogue targeting the catalytic site [29], and KE552 (described as compound L-5 in [11]) that was discovered via a library screening and which has an unknown mechanism of inhibition. Their IC_50_ values of 12 nM for DG013A and 2.1 μΜ for KE552 (Figure S5) were consistent with published results. Of the 33 compounds tested, 6 resulted in significant dose-dependent inhibition profiles and IC_50_ values of 50–200 μM (Table 2 and Figure 4). Eight additional compounds showed limited inhibition at the highest concentration tested and were not considered as hits. None of the 6 hit compounds showed any inhibition of the homologous enzyme ERAP1 at concentrations up to 200 μΜ (Figure S6). However, these hits were less selective for ERAP2, with five compounds demonstrating limited inhibition and compound **3** displaying higher potency for ERAP2 than IRAP (Table 2 and Figure S7).

### 2.6. Inhibition of Oxytocin Trimming

**Figure 5:**
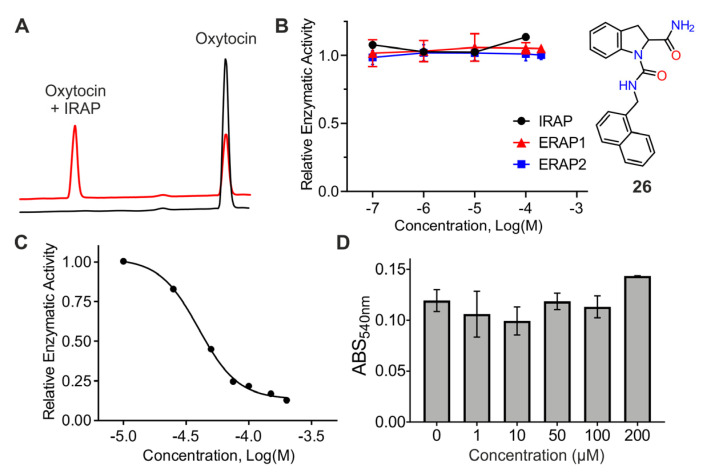
(**A**) Representative chromatograms of trimming of oxytocin by IRAP as monitored by HPLC. (**B**) Effect of compound **26** (inset) on the hydrolysis of 50 μM Leu-AMC by IRAP and ERAP1, and on the hydrolysis of 50 μΜ Arg-AMC by ERAP2. (**C**) Dose-dependent inhibition of IRAP trimming of oxytocin by compound **26.** (**D**) Evaluation of the cellular toxicity of **26** using the MTT assay.

Although small substrates are a useful tool for characterization of inhibitors of aminopeptidases, the allosteric nature of the targeted site and the complex mechanism employed by IRAP [30] prompted us to also characterize the activity of the compounds in inhibiting the hydrolysis of a physiological substrate of IRAP, oxytocin [31]. The cleavage of the N-terminal residue of the cyclic nonapeptide oxytocin can be followed by high-performance liquid chromatography on a C18 reversed phase column (Figure 5A). This assay was validated using DG013A and KE552 as described above for the Leu-AMC substrate. DG013A was able to completely inhibit oxytocin trimming by IRAP when used at a concentration of 1 μM as expected by its calculated IC_50_ for Leu-AMC. Remarkably, KE552 was less effective against oxytocin and inhibited only 10% of trimming at 10 μΜ and up to 38% when used at 100 μM. The % inhibition of IRAP activity for each compound tested is shown in Table 3. The most active compound was found to be **26**, which was, surprisingly, not active in inhibiting the hydrolysis of Leu-AMC by IRAP and ERAP1, nor the hydrolysis of Arg-AMC by ERAP2 (Figure 5B and Figure S8). The ability of **26** to inhibit the processing of oxytocin was found to be dose-dependent with IC_50_ of 41 ± 2 μΜ (Figure 5C).

### 2.7. Evaluation of Cellular Toxicity

Given the interesting properties of compound **26**, we evaluated its toxicity against cultured cells. MOLT4 cells were incubated with **26** at concentrations up to 200 μM for 48 h and their viability was assessed by the standard 3-(4,5-dimethylthiazol-2-yl)-2,5-diphenyltetrazolium bromide (MTT) assay [32]. No apparent toxicity was observed up to 200 μΜ (Figure 5D).

## 3. Discussion

Targeting allosteric sites of enzymes in order to develop inhibitors can be challenging since binding of the compound does not directly compete with binding of the substrate at the catalytic site. At the same time, the conserved nature of many enzymatic active sites, while it can empower rational design, can make achievement of high degrees of selectivity difficult. This has been reported previously for IRAP using phosphinic pseudopeptides that act as transition-state analogues and which are potent inhibitors, but often target homologous aminopeptidases such as ERAP1 and ERAP2 [29]. To circumvent this problem, we targeted an allosteric site in IRAP that has been shown to be able to bind small molecules in the homologous ERAP1 [15]. By combining virtual docking with molecular dynamics and free energy calculations, we were able to identify at least 5 compounds that are selective inhibitors of IRAP with μΜ potencies and could thus serve as leads for the development of more potent inhibitors (Table 2 and Figure 4).

One surprising result from our study is the different behavior of some of the hit compounds when using different substrates. Of the 6 most potent compounds that were validated with the small substrate Leu-AMC of IRAP, 5 were able to display some inhibition of oxytocin (Table 3). However, several more compounds that were not able to inhibit Leu-AMC, did display appreciable inhibition of oxytocin cleavage. One notable such case, compound **26,** was inactive as an inhibitor of Leu-AMC but inhibited oxytocin cleavage with an IC_50_ of 41 μΜ. A previously identified inhibitor, KE552, demonstrated an apparently opposite behavior, being a good inhibitor of Leu-AMC and a poor inhibitor of oxytocin. This apparent dissociation of inhibitory behavior for different size substrates may be due to the relatively low affinity of these compounds which hinders accurate comparisons. Alternatively, the position of binding away from the active site could underlie such phenomena. Modulation of cleavage of the small substrate would have to proceed allosterically through unknown mechanisms. Such a case has been described for the homologous ERAP1, whose activity can be modulated in a substrate-dependent manner by a small compound binding in the malic acid site, by an allosteric/conformational mechanism [16]. Indeed, a spiro-oxindole dihydroquinazolinone derivative was recently demonstrated to be an uncompetitive inhibitor of IRAP and an allosteric binding site adjacent to the zinc catalytic site was proposed [12]. However, inhibition of oxytocin, a substrate that is large enough to reach towards the allosteric site, could also proceed by a more competitive-like mechanism. It should be noted that the only structure of a peptidic substrate with IRAP known is a structure with a linear peptide analogue that does not appear to occupy the allosteric site targeted here (Figure 1) [14]. However, oxytocin, being a cyclic peptide, could bind in a different conformation and at least partially compete with the targeted site. KE552 binding in a distinct site that does not overlap with oxytocin, could underlie its reduced effectiveness. Additional structural studies will be required to clarify the mechanism of inhibition by these compounds.

Although deciphering the exact mechanism of inhibition by allosteric compounds can be difficult without structural information, some insight can be drawn from the known conformational behavior of IRAP and homologous enzymes. IRAP has been crystalized in two distinct conformations in which domains I/II move in relation to domain IV around the hinge domain III and either form an open configuration that exposes the internal cavity to the solvent or a closed configuration that excludes solvent access to the internal cavity [13,14]. Structural analysis using small-angle X-ray scattering has suggested that IRAP in solution is in an open conformation and closes upon inhibitor or substrate binding [33]. As a result, induction of the closed conformation, a conformation that would block substrate–product exchange has been proposed as a possible mechanism of inhibition and could also be the mechanism at work here. A similar mechanism has been proposed for an allosteric inhibitor of ERAP1 [16].

In summary, we describe a virtual docking approach that targets an allosteric site in IRAP and the discovery of several novel and selective IRAP inhibitor leads. Our results suggest that targeting this allosteric site in IRAP is a viable alternative approach for the development of IRAP inhibitors with potential for pharmaceutical applications in cognitive enhancement and the modulation of immune responses.

## 4. Materials and Methods

### 4.1. Docking Calculations

The high-resolution X-ray crystal structures of IRAP in complex with a potent inhibitor (PDB ID: 5mj6) [13] and ERAP1 in complex with a phosphinic pseudopeptide inhibitor (PDB ID: 6q4r) [15] were retrieved from the Protein Data Bank. After removing all heteroatoms, including the inhibitors, their coordinates were superimposed with respect to the domain IV residues comprising a major part of the target pockets. Missing atoms were added using Modeller v9.10 [34] and the H++ server was used to assign protonation states of the titratable groups and add hydrogen atoms accordingly [35]. After removing the non-polar hydrogens, Gasteiger charges and atom types were assigned using AutoDockTools [36]. The search space was defined by a 27-Å cube that was centered within the targeted pocket ((x, y, z) = (5, −54, −23) with reference to PDB ID: 5mj6). AutoDock VINA [21] was used for docking of c.a. 2.45 million lead-like compounds to IRAP, and its top-ranked subset of 24,842 compounds to ERAP1. The default parameters of VINA were retained except for the exhaustiveness level that was increased to 20. The 3D structure of the compounds were obtained from the ZINC15 database [18] in AutoDock PDBQT format and were used without further optimization. Calculations were performed at the HPC facility “ARIS” of the Hellenic National Infrastructures for Research and Technology, GRNeT, which is kindly acknowledged for the allocated time.

### 4.2. Molecular Dynamics Simulations

Molecular dynamics calculations in explicit solvent were performed for 305 complexes of IRAP with selected, top-ranked compounds using the GPU-accelerated version of PMEMD module in AMBER 18 [37,38]. The AMBER *ff14SB* force field [39] was used for IRAP and the *gaff2* force field [40] for the ligands, for which atom types and *AM1-BCC* atomic charges [41] were generated using the ANTECHAMBER module in AMBER. For the metal we employed a simple force field with covalent bonds for tetrahedral Zn(II) [13], after replacing the inhibitor with a water molecule. The complexes were immersed into truncated octahedral solvent boxed of pre-equilibrated TIP3P water molecules with a minimum buffer of 10 Å around the complex, and the required number of counter ions was added to obtain charge neutralization of the systems using the LEaP module in AMBER. The equilibration and production protocol employed was similar to that described in our previous works [13,42,43]. Briefly, a 1-ns equilibration period was followed by 20 ns of unrestrained production runs in the isothermal-isobaric (NPT) ensemble. For the free energy calculations 1000 snapshots were equally sampled from the last 10 ns of the trajectories using the CPPTRAJ module in AMBER [44]. Explicit solvent, counter ions and the metal were removed for the implicit PBSA or GBSA solvent models to be used in the estimation of the solvation energies.

### 4.3. Free energy Calculations

According to the MM/PBSA and MM/GBSA approaches [23,45,46] the binding free energy (Δ*G*_bind_) of a small ligand (L) bound to a protein (P) is typically estimated by the ensemble average of the free protein and ligand extracted from a single simulation of their complex (PL):

(1) $$\Delta G_{\mathrm{bind}}=\langle G_{\mathrm{PL}}-G_{P}-G_{L}\rangle$$

The free energy of each state is estimated from:

(2) $$G=E_{\mathrm{bond}}+E_{\mathrm{el}}+E_{\mathrm{vdW}}+G_{\mathrm{pol}}+G_{\mathrm{np}}-TS$$

where the first three terms are the molecular mechanics energy terms from bonded (*E*_bond_), electrostatic interactions (*E*_el_) and van der Waals interactions (*E*_vdW_), followed by the polar and non-polar contributions to the solvation free energy (*G*_pol_ and *G*_np_, respectively). The last term represents the entropic contribution to the free energy of binding (the absolute temperature *T* multiplied by the entropy *S*) and is often estimated by normal-mode analysis [47]. Considering the high computational cost of the entropy calculations and given that we employed the free energy calculations for a large dataset comparatively, this term was omitted as suggested by others [48].

The polar solvation term *G*_pol_ in Equation (2) is calculated by applying an implicit continuum solvent and using a finite-difference solution or a Generalized Born (GB) pairwise approximation of the Poisson-Boltzmann equation (PB) [24]. The non-polar solvation free energy contribution to the solvation free energy (*G*_np_ in Equation (2)) can be simply estimated as being proportional to the solvent accessible surface area (SASA) of the solute:

(3) $$\Delta G_{\mathrm{np}}=\gamma *\mathrm{SASA}+b$$

where the surface tension *γ* and the correction term b are given constant values for all solute molecules. In more recent approaches [49] the solute cavity formation and the van der Waals interactions free energy terms are estimated as separate terms:

(4) $$\Delta G_{\mathrm{np}}=\Delta G_{\mathrm{disp}}+\gamma *\mathrm{SASA}+b$$

where the dispersion term (Δ*G*_disp_) is often computed using a solvent accessible surface, or a solvent accessible volume integration, and the scaling factors *γ* and b are adjusted according to the choice of the atomic and solvent probe radii employed (see Table 4 below).

In this work, we employed two MM/PBSA and two MM/GBSA parameter sets designated as *PB-1* and *PB-4* for the MM/PBSA, and *GB-1* and *GB-5* for the MM/GBSA approach (Table 4), in reference to AMBER 18 manual (https://ambermd.org/doc12/Amber18.pdf; accessed on 3 June 2021). Post-processing of the snapshots taken from the MD trajectories was performed with the MPI version of the *MMPBSA* python script in AMBER 18. Selection of the compounds for experimental testing was mainly based on the free energy results of MM/PBSA method *PB-1*, followed by a selection of highly ranked compounds as given by *PB-4* and the two MM/GBSA methods, in conjunction with their relative ranking in all four methods. The free energy results and energy decomposition for the 305 compounds using the four methods are provided within the Supplementary Material (Tables S3–S6) and a summary of the results obtained for the 33 selected compounds shown in Figure S2 is given in Table S7.

### 4.4. Protein Production and Purification

The extracellular domain of human IRAP was isolated in recombinant form from stably transfected HEK 293S GnTI^(-)^ cells as described previously [14]. Human recombinant ERAP1 and ERAP2 was isolated from Hi5 cells infected by baculovirus as described previously [42,56].

### 4.5. Enzymatic Assays

Enzymatic assays using the substrate L-Leucine-7-amido-4-coumarin (Sigma, St. Louis, MO, USA) or L-Arginine-7-amido-4-coumarin (Sigma) were performed as described previously [42]. Oxytocin cleavage by IRAP was followed by high-performance liquid chromatography. Briefly, oxytocin (40 μM) was incubated with recombinant IRAP (2–200 nM) at 37°C for 30 min and the reaction was terminated by adding 0.25% (*v*/*v*) trifluoroacetic acid (TFA) and kept at –80 °C until analysis. The reactions were analyzed using a reversed phase C18 column (chromolith C-18 column, Merck, Kenilworth, NJ, USA) by following the absorbance at 220 nm using a linear gradient (solvent A: 0.05% TFA, 10% acetonitrile, solvent B: 0.05% TFA, 40% acetonitrile). The percentage of substrate cleaved was calculated by integrating the surface of the substrate and product peaks.

### 4.6. Inhibitors

Compound KE552 (described as compound L-5 in [11]) was generously provided by Dr. Mathias Hallberg (Uppsala University, Sweden). Compound DG013A (described in [29]) was generously provided by Dr. Dimitris Georgiadis (National and Kapodistrian University of Athens, Greece). Compounds **1**–**33** were purchased from MolPort and were used without further purification, as mixtures of enantiomers or diastereomers where applicable (Table S7).

### 4.7. Cellular Toxicity Assay

MOLT4 cells (CRL-1582 from ATCC, 5000 cells/well) were cultured in 100 μL RPMI 1640 supplemented with 2 mM glutamine, 10% heat-inactivated FBS (Gibco), 1% penicillin and streptomycin and incubated at 37 °C, 5% CO_2_ in the presence of varying concentrations of compound **26** (0–200 μM) for 48 h. After the incubation, 100 μL of RPMI 1640 containing 2 mg/mL 3-(4,5-dimethylthiazol-2-yl)-2,5-diphenyltetrazolium bromide (MTT reagent) were added to each well. The cells were allowed to rest for 4 h and then centrifuged at 1250 rpm for 5 min at room temperature. The resulting formazan crystals were dissolved in DMSO (100 μL) and the absorbance intensity measured on a TECAN infinite M200 microplate fluorescence reader at 540 nm with reference at 620 nm. All experiments were performed in triplicate.

## Figures and Tables

**Table 1:** The final set of 33 compounds obtained for experimental evaluation, indicating docking scores against IRAP and ERAP1, and estimated binding free energy obtained from MM/PBSA calculations using two parameter sets. Energies are in kcal/mol and ranking (#) is relative in each set.

ID	MolPort ID	MW	log*P* ^*a*^	VINA Score		MM/PBSA Δ*G*_bind_	
				IRAP	ERAP1	*PB-1* (Rank)	*PB-4* (Rank)
**1**	MolPort-027-863-667	342.4	2.85	–10.2	–7.5	–1.3 (#105)	–30.3 (#59)
**2**	MolPort-039-039-060	331.4	2.38	–11.0	–9.2	–8.1 (#17)	–31.5 (#41)
**3**	MolPort-005-163-536	330.4	3.45	–9.9	–7.1	–10.8 (#6)	–32.6 (#29)
**4**	MolPort-027-857-260	341.5	2.96	–9.9	–7.4	–7.8 (#20)	–36.9 (#3)
**5**	MolPort-038-425-524	335.4	2.34	–11.0	–8.9	–7.5 (#22)	–33.5 (#17)
**6**	MolPort-006-484-933	335.4	2.71	–9.9	–7.4	–14.1 (#2)	–38.7 (#1)
**7**	MolPort-007-773-311	323.4	3.2	–10.6	–8.1	–5.0 (#42)	–29.1 (#78)
**8**	MolPort-027-906-298	332.4	2.75	–10.2	–7.4	–7.4 (#23)	–32.5 (#31)
**9**	MolPort-035-834-132	348.4	3.07	–11.1	–10.0	–4.5 (#51)	–35.4 (#9)
**10**	MolPort-020-152-989	348.4	2.96	–10.4	–7.9	–9.8 (#9)	–33.7 (#14)
**11**	MolPort-019-674-325	338.4	2.19	–11.1	–8.0	–8.0 (#19)	–32.6 (#30)
**12**	MolPort-020-162-189	330.4	2.15	–10.9	–9.5	–5.0 (#43)	–27.2 (#119)
**13**	MolPort-028-771-825	348.4	1.58	–11.1	–8.5	–6.5 (#27)	–35.4 (#8)
**14**	MolPort-009-543-745	326.3	2.42	–10.9	–8.5	–8.0 (#18)	–29.5 (#72)
**15**	MolPort-004-188-947	333.5	2.97	–10.1	–7.1	0.3 (#142)	–26.4 (#137)
**16**	MolPort-027-679-372	332.4	2.77	–10.9	–9.5	–10.5 (#8)	–36.2 (#6)
**17**	MolPort-005-610-771	334.3	1.59	–10.9	–9.6	–9.7 (#10)	–36.0 (#7)
**18**	MolPort-009-519-757	345.4	2.34	–10.9	–8.7	–5.1 (#41)	–33.1 (#22)
**19**	MolPort-039-259-003	322.4	1.78	–10.9	–8.6	–7.0 (#25)	–32.2 (#32)
**20**	MolPort-009-450-517	346.4	2.62	–10.9	–9.2	–9.2 (#12)	–35.1 (#10)
**21**	MolPort-004-164-037	349.5	3.37	–10.8	–8.1	–7.3 (#24)	–37.7 (#2)
**22**	MolPort-027-704-758	324.4	1.77	–10.5	–8.0	–13.9 (#3)	–36.3 (#4)
**23**	MolPort-020-057-841	333.4	2.94	–10.9	–9.2	–5.8 (#33)	–32.9 (#25)
**24**	MolPort-009-027-892	347.4	3.02	–10.7	–8.1	–3.0 (#75)	–31.0 (#46)
**25**	MolPort-020-053-566	338.4	2.87	–9.8	–7.1	–7.7 (#21)	–33.2 (#20)
**26**	MolPort-003-173-337	345.4	2.34	–11.0	–9.7	–15.7 (#1)	–34.3 (#13)
**27**	MolPort-003-801-767	332.4	1.81	–10.9	–9.0	–8.3 (#16)	–33.6 (#15)
**28**	MolPort-000-832-181	346.4	2.82	–10.9	–9.3	–3.9 (#68)	–30.8 (#53)
**29**	MolPort-000-828-576	333.4	2.72	–10.9	–8.3	–4.7 (#50)	–29.3 (#75)
**30**	MolPort-000-694-123	336.4	2.95	–10.2	–7.7	–9.5 (#11)	–33.4 (#18)
**31**	MolPort-023-297-297	336.4	2.91	–10.4	–7.8	–10.5 (#7)	–34.4 (#12)
**32**	MolPort-000-407-697	325.4	3.47	–10.1	–7.6	–8.6 (#14)	–28.5 (#92)
**33**	MolPort-001-905-390	323.4	3.06	–10.3	–7.7	–4.4 (#53)	–31.0 (#50)

*^a^* Calculated n-octanol/water partition coefficient using the implicit log P method, iLOGP [27].

**Table 2:** IC_50_ values for compounds **1**–**33** calculated from the small fluorogenic substrate assays.

ID	IC_50_ (μM)			ID	IC_50_ (μM)			ID	IC_50_ (μM)		
	IRAP	ERAP1	ERAP2		IRAP	ERAP1	ERAP2		IRAP	ERAP1	ERAP2
**1**	>300 *^a^*	-	-	**12**	*NI*	-	-	**23**	>300 *^a^*	-	-
**2**	*NI*	-	-	**13**	192 ± 21	*NI*	>300 *^a^*	**24**	>300 *^a^*	-	-
**3**	192 ± 35	*NI*	65 ± 5	**14**	*NI*	-	-	**25**	*NI*	*-*	*-*
**4**	*NI*	-	-	**15**	*NI*	-	-	**26**	*NI*	*-*	*-*
**5**	76 ± 9	*NI*	>300 *^a^*	**16**	*NI*	-	-	**27**	*NI*	*-*	*-*
**6**	*NI*	-	-	**17**	56 ± 6	*NI*	280 ± 54	**28**	92 ± 10	*NI*	>300 *^a^*
**7**	*NI*	-	-	**18**	>300 *^a^*	-	-	**29**	*NI*	*-*	*-*
**8**	*NI*	-	-	**19**	>300 *^a^*	-	-	**30**	*NI*	*-*	*-*
**9**	*NI*	-	-	**20**	>300 *^a^*	-	-	**31**	194 ± 24	*NI*	>300 *^a^*
**10**	*NI*	-	-	**21**	*NI*	-	-	**32**	*NI*	*-*	*-*
**11**	>300 *^a^*	-	-	**22**	>300 *^a^*	-	-	**33**	*NI*	*-*	*-*

*^a^* Limited inhibition observed only at the highest concentration tested; *NI*: no inhibition; (-) not tested.

**Table 3:** Inhibition of oxytocin trimming by IRAP in the presence of **1**–**33** and the two known inhibitors, DG013A and KE552, at a concentration of 100 μΜ.

ID	% Inhibition	ID	% Inhibition	ID	% Inhibition
**1**	0	**12**	44	**23**	26
**2**	26	**13**	10	**24**	0
**3**	7	**14**	25	**25**	14
**4**	11	**15**	9	**26**	69
**5**	17	**16**	32	**27**	32
**6**	15	**17**	23	**28**	*n.d.*
**7**	0	**18**	0	**29**	39
**8**	0	**19**	0	**30**	22
**9**	30	**20**	34	**31**	10
**10**	0	**21**	14	**32**	28
**11**	13	**22**	0	**33**	10
**KE552**	38	**ΚΕ552** *^a^*	10	**DG013A** *^b^*	100

*n.d.* not determined due to peak overlap; *^a^* KE552 also tested at 10 μΜ; *^b^* DG013A tested at 1 μΜ.

**Table 4:** Summary of the parameter sets employed in free energy calculations, indicating the method used for calculation of the polar (*G*_pol_) and the non-polar solvation energy (*G*_np_) terms.

Alias	*G*_pol_ Method	Atomic Radii	*G*_np_ Method	SASA *^c^*	*γ*	b
*PB-1*	PBSA ^*a*^	TL-mbondi ^*b*^	Equation (4)	PBSA ^*d*^	0.03780	−0.5692
*PB-4*	PBSA ^*a*^	mbondi [50]	Equation (3)	Molsurf [51]	0.00720	0.0000
*GB-1*	GB^HTC^ [52]	mbondi [50]	Equation (3)	LCPO [53]	0.00720	0.0000
*GB-5*	GB^OTC-II^ [54]	mbondi2 [54]	Equation (3)	LCPO [53]	0.00500	0.0000

*^a^* Poisson-Boltzmann solver PICCG as implemented in the *PBSA* module of AMBER 18. *^b^* Radii for protein atoms as optimized by Tan & Luo [55], with mbondi radii for the ligand [50]. *^c^* Method or program used for the calculation of the solvent-accessible surface area. *^d^* Δ*G*_disp_ calculated by a numerical determination of the solvent accessible surface area [49].

## Data Availability

The data presented in this study are available on reasonable request from the corresponding authors.

## Supplementary Materials

The following are available online at https://www.mdpi.com/article/10.3390/ph14060584/s1, Detailed computational results (Figures S1–S4 and Tables S1–S8), and supplementary experimental results in Figures S5–S8.
