# Supplementary material for: Discovery of Selective Inhibitor Leads by Targeting an Allosteric Site in Insulin-Regulated Aminopeptidase
Source: Pharmaceuticals (Basel). 2021 Jun 18;14(6):584. doi: 10.3390/ph14060584 (PMC8233869; doi:10.3390/ph14060584)
Supplement: Supplementary file 1 [file pharmaceuticals-14-00584-s001.zip › pharmaceuticals-1267655-supplementary.pdf]

# Supplementary Material

# Discovery of selective inhibitor leads by targeting an allosteric site in Insulin-Regulated Aminopeptidase

**Ioannis Temponeras[1], Lykourgos Chiniadis[1], Athanasios Papakyriakou[*,1] and Efstratios Stratikos[*,1,2]**

[1] National Centre for Scientific Research Demokritos, Agia Paraskevi, Greece
[2] Department of Chemistry, National and Kapodistrian University of Athens, Greece
[*] Correspondence: thpap@bio.demokritos.gr or estratikos@chem.uoa.gr

## Table of Contents

Figure S1 .............................................. 2
Table S1 .............................................. 3
Table S2 .............................................. 8
Table S3 .............................................. 13
Table S4 .............................................. 20
Table S5 .............................................. 27
Table S6 .............................................. 34
Table S7 .............................................. 41
Figure S2 .............................................. 43
Figure S3 .............................................. 44
Figure S4 .............................................. 45
Table S8 .............................................. 47
Figure S5 .............................................. 52
Figure S6 .............................................. 53
Figure S7 .............................................. 54
Figure S8 .............................................. 55

Rep. | 2D | 3D | React. | Standard ▾ | Purch. | In-Stock ▾ | pH | Ref Mid ▾ | Charge | -2 -1 0 +1 +2 ▾

**Molecular Weight (up to, Daltons)**

| LogP (up to) | 200 | 250 | 300 | 325 | 350 | 375 | 400 | 425 | 450 | 500 | >500 | Totals, by LogP |
|---|---|---|---|---|---|---|---|---|---|---|---|---|
| -1 | 7,719 | 6,132 | 7,451 | 4,008 | 3,677 | 2,632 | 1,651 | 1,207 | 1,054 | 1,422 | 2,841 | 39,794 |
| 0 | 29,273 | 21,724 | 33,586 | 21,166 | 21,547 | 13,000 | 6,809 | 4,249 | 2,871 | 2,853 | 2,805 | 159,883 |
| 1 | 77,696 | 88,965 | 157,807 | 112,036 | 115,193 | 66,828 | 33,838 | 20,413 | 13,093 | 12,868 | 6,156 | 704,893 |
| 2 | 100,929 | 187,772 | 396,402 | 319,413 | 231,293 | 231,825 | 126,158 | 82,640 | 58,856 | 52,909 | 16,812 | 1,805,009 |
| 2.5 | 35,177 | 105,806 | 280,909 | 250,419 | 326,514 | 206,142 | 128,713 | 95,823 | 71,193 | 65,420 | 19,493 | 1,585,609 |
| 3 | 20,249 | 84,355 | 262,517 | 248,186 | 377,939 | 282,752 | 181,246 | 141,630 | 111,534 | 110,991 | 35,445 | 1,856,844 |
| 3.5 | 8,732 | 52,427 | 198,754 | 218,204 | 325,910 | 281,982 | 215,073 | 183,732 | 151,939 | 160,634 | 61,553 | 1,858,940 |
| 4 | 2,609 | 24,056 | 117,310 | 139,596 | 234,162 | 240,875 | 255,592 | 216,924 | 181,436 | 208,383 | 96,290 | 1,717,233 |
| 4.5 | 542 | 8,213 | 54,255 | 80,431 | 139,126 | 175,095 | 197,418 | 206,406 | 185,318 | 231,640 | 110,254 | 1,388,698 |
| 5 | 93 | 2,271 | 20,547 | 35,136 | 68,741 | 103,831 | 120,103 | 150,108 | 158,689 | 228,537 | 170,303 | 1,058,359 |
| >5 | 27 | 750 | 8,702 | 18,028 | 43,936 | 84,740 | 119,766 | 180,013 | 222,032 | 478,712 | 848,071 | 2,004,777 |
| **Totals, by Weight** | 283,046 | 582,471 | 1,538,240 | 1,446,623 | 1,888,038 | 1,689,702 | 1,386,367 | 1,283,145 | 1,158,015 | 1,554,369 | 1,370,023 | **14M** Protomers **8.2K** Tranches |

Rep. | 2D | 3D | React. | Anodyne ▾ | Purch. | In-Stock ▾ | pH | Ref ▾ | Charge | -2 -1 0 +1 +2 ▾

**Molecular Weight (up to, Daltons)**

| LogP (up to) | 200 | 250 | 300 | 325 | 350 | 375 | 400 | 425 | 450 | 500 | >500 | Totals, by LogP |
|---|---|---|---|---|---|---|---|---|---|---|---|---|
| -1 | 5,811 | 4,565 | 5,585 | 2,895 | 2,687 | 1,947 | 1,190 | 817 | 610 | 872 | 1,580 | 15,732 |
| 0 | 21,311 | 15,619 | 23,920 | 15,050 | 15,757 | 9,840 | 5,179 | 3,225 | 2,002 | 1,974 | 1,792 | 70,346 |
| 1 | 55,040 | 61,897 | 110,983 | 79,182 | 82,677 | 49,668 | 25,497 | 15,351 | 9,930 | 9,625 | 4,142 | 334,739 |
| 2 | 72,651 | 131,269 | 280,168 | 225,896 | 165,025 | 169,663 | 93,472 | 61,725 | 43,919 | 39,501 | 11,225 | 802,358 |
| 2.5 | 26,171 | 75,496 | 199,503 | 178,925 | 235,035 | 149,771 | 94,225 | 70,224 | 51,985 | 47,917 | 12,815 | 688,959 |
| 3 | 15,404 | 60,977 | 187,239 | 178,061 | 274,897 | 204,830 | 133,169 | 103,335 | 81,027 | 80,171 | 23,101 | 701,174 |
| 3.5 | 6,767 | 38,433 | 141,659 | 158,058 | 241,061 | 204,940 | 157,607 | 133,892 | 110,285 | 115,848 | 40,814 | 0 |
| 4 | 1,960 | 17,971 | 85,298 | 101,696 | 173,946 | 175,929 | 186,322 | 158,014 | 130,211 | 147,318 | 63,844 | 0 |
| 4.5 | 398 | 5,639 | 39,665 | 59,418 | 103,205 | 129,648 | 143,846 | 149,535 | 131,741 | 160,867 | 80,207 | 0 |
| 5 | 54 | 1,395 | 14,255 | 25,796 | 51,378 | 77,184 | 67,985 | 108,443 | 113,047 | 157,521 | 111,630 | 0 |
| >5 | 8 | 404 | 4,837 | 11,551 | 30,356 | 60,157 | 85,261 | 125,113 | 151,823 | 309,900 | 489,345 | 0 |
| **Totals, by Weight** | 0 | 349,823 | 807,398 | 680,009 | 776,078 | 0 | 0 | 0 | 0 | 0 | 0 | **2.6M** Protomers **240** Tranches |

**Figure S1.** Chemical space of ZINC15 in tranches of 3D protomers according to size (MW) and hydrophobicity (LogP), for a total of 14 million readily available compounds (in-stock with standard reactivity, upper) and the lead-like subset selected (lower). Screenshots were taken in March 2019.

**Table S1.** The top-ranked selection of compounds based on their estimated binding affinity for IRAP (given as VINA score in kcal/mol). The corresponding estimated affinity for ERAP1 and their difference to IRAP are also given as *Delta* = $\Delta(\Delta G_{bind}^{(IRAP)} - \Delta G_{bind}^{(ERAP1)}$.

| # | ZINC ID | VINA score (kcal/mol) | | |
|---|---|---|---|---|
| | | IRAP | ERAP1 | *Delta* |
| 1 | ZINC000257220067 | -11.7 | -8.9 | -2.8 |
| 2 | ZINC000034895992 | -11.6 | -9.6 | -2.0 |
| 3 | ZINC000041548359 | -11.6 | -9.1 | -2.5 |
| 4 | ZINC000012550491 | -11.6 | -8.9 | -2.7 |
| 5 | ZINC000003617904 | -11.6 | -8.8 | -2.8 |
| 6 | ZINC000006117573 | -11.5 | -10.9 | -0.6 |
| 7 | ZINC000022241454 | -11.5 | -10.2 | -1.3 |
| 8 | ZINC000000489138 | -11.5 | -9.8 | -1.7 |
| 9 | ZINC000014689114 | -11.5 | -9.8 | -1.7 |
| 10 | ZINC000255187536 | -11.5 | -9.5 | -2.0 |
| 11 | ZINC000100191495 | -11.5 | -9.4 | -2.1 |
| 12 | ZINC000072807915 | -11.5 | -9.3 | -2.2 |
| 13 | ZINC000100193430 | -11.5 | -9.3 | -2.2 |
| 14 | ZINC000003947438 | -11.4 | -9.8 | -1.6 |
| 15 | ZINC000004722089 | -11.4 | -9.8 | -1.6 |
| 16 | ZINC000001810957 | -11.4 | -9.6 | -1.8 |
| 17 | ZINC000016543876 | -11.4 | -9.3 | -2.1 |
| 18 | ZINC000068269627 | -11.4 | -9.3 | -2.1 |
| 19 | ZINC000009318779 | -11.4 | -9.2 | -2.2 |
| 20 | ZINC000257310083 | -11.4 | -9.0 | -2.4 |
| 21 | ZINC000067263105 | -11.3 | -10.1 | -1.2 |
| 22 | ZINC000018053795 | -11.3 | -9.7 | -1.6 |
| 23 | ZINC000085879141 | -11.3 | -9.6 | -1.7 |
| 24 | ZINC000045070892 | -11.3 | -9.4 | -1.9 |
| 25 | ZINC000084686490 | -11.3 | -9.3 | -2.0 |
| 26 | ZINC000008848147 | -11.3 | -9.2 | -2.1 |
| 27 | ZINC000035398834 | -11.3 | -9.2 | -2.1 |
| 28 | ZINC000225746732 | -11.3 | -9.2 | -2.1 |
| 29 | ZINC000062639858 | -11.3 | -9.1 | -2.2 |
| 30 | ZINC000000187795 | -11.3 | -9.0 | -2.3 |
| 31 | ZINC000012557331 | -11.3 | -8.9 | -2.4 |
| 32 | ZINC000253433259 | -11.3 | -7.8 | -3.5 |
| 33 | ZINC000012323933 | -11.2 | -10.1 | -1.1 |
| 34 | ZINC000007030710 | -11.2 | -9.7 | -1.5 |
| 35 | ZINC000257206610 | -11.2 | -9.7 | -1.5 |
| 36 | ZINC000067643674 | -11.2 | -9.6 | -1.6 |
| 37 | ZINC000014689108 | -11.2 | -9.5 | -1.7 |
| 38 | ZINC000012557330 | -11.2 | -9.4 | -1.8 |
| 39 | ZINC000013278475 | -11.2 | -9.4 | -1.8 |
| 40 | ZINC000044908085 | -11.2 | -9.4 | -1.8 |
| 41 | ZINC000048249797 | -11.2 | -9.4 | -1.8 |
| 42 | ZINC000061067795 | -11.2 | -9.4 | -1.8 |

| # | ZINC ID | VINA score (kcal/mol) | | |
|---|---|---|---|---|
| | | IRAP | ERAP1 | *Delta* |
| 43 | ZINC000009338249 | -11.2 | -9.3 | -1.9 |
| 44 | ZINC000028790813 | -11.2 | -9.3 | -1.9 |
| 45 | ZINC000089857747 | -11.2 | -9.2 | -2.0 |
| 46 | ZINC000225752266 | -11.2 | -9.1 | -2.1 |
| 47 | ZINC000001431797 | -11.2 | -9.0 | -2.2 |
| 48 | ZINC000095951555 | -11.2 | -9.0 | -2.2 |
| 49 | ZINC000097413355 | -11.2 | -8.9 | -2.3 |
| 50 | ZINC000001620500 | -11.2 | -8.6 | -2.6 |
| 51 | ZINC000040386288 | -11.2 | -8.6 | -2.6 |
| 52 | ZINC000077522487 | -11.2 | -8.5 | -2.7 |
| 53 | ZINC000023995834 | -11.2 | -8.4 | -2.8 |
| 54 | ZINC000040473994 | -11.2 | -8.4 | -2.8 |
| 55 | ZINC000585093224 | -11.2 | -8.4 | -2.8 |
| 56 | ZINC000239762559 | -11.2 | -7.8 | -3.4 |
| 57 | ZINC000000136922 | -11.1 | -10.2 | -0.9 |
| **58** | **ZINC000057563200** | **-11.1** | **-10.0** | **-1.1** |
| 59 | ZINC000044874685 | -11.1 | -9.9 | -1.2 |
| 60 | ZINC000192753414 | -11.1 | -9.9 | -1.2 |
| 61 | ZINC000069775761 | -11.1 | -9.8 | -1.3 |
| 62 | ZINC000004003439 | -11.1 | -9.7 | -1.4 |
| 63 | ZINC000030875068 | -11.1 | -9.7 | -1.4 |
| 64 | ZINC000072437791 | -11.1 | -9.7 | -1.4 |
| 65 | ZINC000095393415 | -11.1 | -9.6 | -1.5 |
| 66 | ZINC000014791001 | -11.1 | -9.5 | -1.6 |
| 67 | ZINC000268943077 | -11.1 | -9.5 | -1.6 |
| 68 | ZINC000000082526 | -11.1 | -9.4 | -1.7 |
| 69 | ZINC000007030648 | -11.1 | -9.4 | -1.7 |
| 70 | ZINC000072300732 | -11.1 | -9.4 | -1.7 |
| 71 | ZINC000003617861 | -11.1 | -9.2 | -1.9 |
| 72 | ZINC000057771728 | -11.1 | -9.2 | -1.9 |
| 73 | ZINC000077503523 | -11.1 | -9.2 | -1.9 |
| 74 | ZINC000078914774 | -11.1 | -9.2 | -1.9 |
| 75 | ZINC000009318781 | -11.1 | -9.1 | -2.0 |
| 76 | ZINC000019327176 | -11.1 | -9.0 | -2.1 |
| 77 | ZINC000084563579 | -11.1 | -9.0 | -2.1 |
| 78 | ZINC000013562617 | -11.1 | -8.9 | -2.2 |
| 79 | ZINC000013691850 | -11.1 | -8.9 | -2.2 |
| 80 | ZINC000072290626 | -11.1 | -8.8 | -2.3 |
| 81 | ZINC000194368147 | -11.1 | -8.7 | -2.4 |
| 82 | ZINC000299785131 | -11.1 | -8.6 | -2.5 |
| **83** | **ZINC000095415912** | **-11.1** | **-8.5** | **-2.6** |
| 84 | ZINC000000065653 | -11.1 | -8.4 | -2.7 |
| 85 | ZINC000050857093 | -11.1 | -8.2 | -2.9 |
| 86 | ZINC000100756934 | -11.1 | -8.1 | -3.0 |
| **87** | **ZINC000051467674** | **-11.1** | **-8.0** | **-3.1** |

| # | ZINC ID | VINA score (kcal/mol) | | |
|---|---|---|---|---|
| | | IRAP | ERAP1 | *Delta* |
| 88 | ZINC000004807849 | -11.0 | -10.4 | -0.6 |
| 89 | ZINC000018177963 | -11.0 | -10.4 | -0.6 |
| 90 | ZINC000005426253 | -11.0 | -10.3 | -0.7 |
| 91 | ZINC000017888288 | -11.0 | -10.3 | -0.7 |
| 92 | ZINC000052955795 | -11.0 | -10.2 | -0.8 |
| 93 | ZINC000095469884 | -11.0 | -10.2 | -0.8 |
| 94 | ZINC000012323932 | -11.0 | -10.0 | -1.0 |
| 95 | ZINC000230113696 | -11.0 | -9.8 | -1.2 |
| 96 | ZINC000005893172 | -11.0 | -9.7 | -1.3 |
| **97** | **ZINC000010256259** | **-11.0** | **-9.7** | **-1.3** |
| 98 | ZINC000065167646 | -11.0 | -9.7 | -1.3 |
| 99 | ZINC000072896858 | -11.0 | -9.7 | -1.3 |
| 100 | ZINC000136345745 | -11.0 | -9.7 | -1.3 |
| 101 | ZINC000004100408 | -11.0 | -9.6 | -1.4 |
| 102 | ZINC000013973188 | -11.0 | -9.6 | -1.4 |
| 103 | ZINC000016228943 | -11.0 | -9.6 | -1.4 |
| 104 | ZINC000065925006 | -11.0 | -9.6 | -1.4 |
| 105 | ZINC000072896859 | -11.0 | -9.6 | -1.4 |
| 106 | ZINC000029134571 | -11.0 | -9.5 | -1.5 |
| 107 | ZINC000035671101 | -11.0 | -9.5 | -1.5 |
| 108 | ZINC000104346290 | -11.0 | -9.5 | -1.5 |
| 109 | ZINC000008771448 | -11.0 | -9.4 | -1.6 |
| 110 | ZINC000008776545 | -11.0 | -9.4 | -1.6 |
| 111 | ZINC000021835963 | -11.0 | -9.4 | -1.6 |
| 112 | ZINC000178511392 | -11.0 | -9.4 | -1.6 |
| 113 | ZINC000096122792 | -11.0 | -9.3 | -1.7 |
| 114 | ZINC000003313076 | -11.0 | -9.2 | -1.8 |
| 115 | ZINC000095387190 | -11.0 | -9.2 | -1.8 |
| 116 | ZINC000100193536 | -11.0 | -9.2 | -1.8 |
| **117** | **ZINC000244816835** | **-11.0** | **-9.2** | **-1.8** |
| 118 | ZINC000072290711 | -11.0 | -9.1 | -1.9 |
| 119 | ZINC000000088011 | -11.0 | -9.0 | -2.0 |
| 120 | ZINC000006086854 | -11.0 | -9.0 | -2.0 |
| 121 | ZINC000018181694 | -11.0 | -9.0 | -2.0 |
| 122 | ZINC000019973960 | -11.0 | -9.0 | -2.0 |
| 123 | ZINC000257211885 | -11.0 | -9.0 | -2.0 |
| 124 | ZINC000058202763 | -11.0 | -8.9 | -2.1 |
| 125 | ZINC000095447759 | -11.0 | -8.9 | -2.1 |
| **126** | **ZINC000223692657** | **-11.0** | **-8.9** | **-2.1** |
| 127 | ZINC000089827813 | -11.0 | -8.8 | -2.2 |
| 128 | ZINC000004722011 | -11.0 | -8.6 | -2.4 |
| 129 | ZINC000019796838 | -11.0 | -8.6 | -2.4 |
| 130 | ZINC000257257831 | -11.0 | -8.6 | -2.4 |
| 131 | ZINC000096238948 | -11.0 | -8.5 | -2.5 |
| 132 | ZINC000171342968 | -11.0 | -8.5 | -2.5 |

| # | ZINC ID | VINA score (kcal/mol) | | |
|---|---|---|---|---|
| | | IRAP | ERAP1 | *Delta* |
| 133 | ZINC000178206155 | -11.0 | -8.5 | -2.5 |
| 134 | ZINC000000439839 | -11.0 | -8.4 | -2.6 |
| 135 | ZINC000096837115 | -10.9 | -10.7 | -0.2 |
| 136 | ZINC000002465083 | -10.9 | -10.6 | -0.3 |
| 137 | ZINC000006117574 | -10.9 | -10.6 | -0.3 |
| 138 | ZINC000575617533 | -10.9 | -10.4 | -0.5 |
| 139 | ZINC000003852421 | -10.9 | -9.9 | -1.0 |
| 140 | ZINC000057511709 | -10.9 | -9.9 | -1.0 |
| 141 | ZINC000072290753 | -10.9 | -9.9 | -1.0 |
| 142 | ZINC000003875380 | -10.9 | -9.8 | -1.1 |
| 143 | ZINC000013691849 | -10.9 | -9.8 | -1.1 |
| 144 | ZINC000019973951 | -10.9 | -9.8 | -1.1 |
| 145 | ZINC000072277544 | -10.9 | -9.8 | -1.1 |
| 146 | ZINC000022549345 | -10.9 | -9.7 | -1.2 |
| 147 | ZINC000034407910 | -10.9 | -9.7 | -1.2 |
| 148 | ZINC000299793701 | -10.9 | -9.7 | -1.2 |
| **149** | **ZINC000005360885** | **-10.9** | **-9.6** | **-1.3** |
| 150 | ZINC000001758646 | -10.9 | -9.5 | -1.4 |
| 151 | ZINC000004003438 | -10.9 | -9.5 | -1.4 |
| 152 | ZINC000005088834 | -10.9 | -9.5 | -1.4 |
| 153 | ZINC000014240188 | -10.9 | -9.5 | -1.4 |
| 154 | ZINC000029134573 | -10.9 | -9.5 | -1.4 |
| **155** | **ZINC000069778215** | **-10.9** | **-9.5** | **-1.4** |
| 156 | ZINC000084598174 | -10.9 | -9.5 | -1.4 |
| **157** | **ZINC000084598177** | **-10.9** | **-9.5** | **-1.4** |
| 158 | ZINC000003438521 | -10.9 | -9.4 | -1.5 |
| 159 | ZINC000008316344 | -10.9 | -9.4 | -1.5 |
| 160 | ZINC000003245357 | -10.9 | -9.3 | -1.6 |
| **161** | **ZINC000004998502** | **-10.9** | **-9.3** | **-1.6** |
| 162 | ZINC000006556210 | -10.9 | -9.3 | -1.6 |
| 163 | ZINC000010218408 | -10.9 | -9.3 | -1.6 |
| 164 | ZINC000040266307 | -10.9 | -9.3 | -1.6 |
| 165 | ZINC000050859891 | -10.9 | -9.3 | -1.6 |
| 166 | ZINC000072432742 | -10.9 | -9.3 | -1.6 |
| 167 | ZINC000119429896 | -10.9 | -9.3 | -1.6 |
| 168 | ZINC000257206611 | -10.9 | -9.3 | -1.6 |
| 169 | ZINC000000390374 | -10.9 | -9.2 | -1.7 |
| **170** | **ZINC000022191233** | **-10.9** | **-9.2** | **-1.7** |
| **171** | **ZINC000071915224** | **-10.9** | **-9.2** | **-1.7** |
| 172 | ZINC000001711692 | -10.9 | -9.1 | -1.8 |
| 173 | ZINC000006576136 | -10.9 | -9.1 | -1.8 |
| 174 | ZINC000008738218 | -10.9 | -9.1 | -1.8 |
| 175 | ZINC000012875967 | -10.9 | -9.1 | -1.8 |
| 176 | ZINC000153353600 | -10.9 | -9.1 | -1.8 |
| 177 | ZINC000229224134 | -10.9 | -9.1 | -1.8 |

| # | ZINC ID | VINA score (kcal/mol) | | |
|---|---|---|---|---|
| | | IRAP | ERAP1 | *Delta* |
| 178 | ZINC000584897275 | -10.9 | -9.1 | -1.8 |
| **179** | **ZINC000000333210** | **-10.9** | **-9.0** | **-1.9** |
| 180 | ZINC000002465084 | -10.9 | -9.0 | -1.9 |
| 181 | ZINC000004535480 | -10.9 | -9.0 | -1.9 |
| 182 | ZINC000047209496 | -10.9 | -9.0 | -1.9 |
| 183 | ZINC000135810445 | -10.9 | -9.0 | -1.9 |
| 184 | ZINC000253433605 | -10.9 | -9.0 | -1.9 |
| 185 | ZINC000002646979 | -10.9 | -8.9 | -2.0 |
| 186 | ZINC000011781217 | -10.9 | -8.9 | -2.0 |
| 187 | ZINC000072300733 | -10.9 | -8.9 | -2.0 |
| 188 | ZINC000075530760 | -10.9 | -8.9 | -2.0 |
| 189 | ZINC000091943530 | -10.9 | -8.9 | -2.0 |
| 190 | ZINC000020116324 | -10.9 | -8.8 | -2.1 |
| 191 | ZINC000050857092 | -10.9 | -8.8 | -2.1 |
| 192 | ZINC000069538683 | -10.9 | -8.8 | -2.1 |
| 193 | ZINC000074014922 | -10.9 | -8.8 | -2.1 |
| 194 | ZINC000284798034 | -10.9 | -8.8 | -2.1 |
| 195 | ZINC000001465257 | -10.9 | -8.7 | -2.2 |
| 196 | ZINC000002922856 | -10.9 | -8.7 | -2.2 |
| 197 | ZINC000004732939 | -10.9 | -8.7 | -2.2 |
| 198 | ZINC000005776998 | -10.9 | -8.7 | -2.2 |
| 199 | ZINC000006887927 | -10.9 | -8.7 | -2.2 |
| 200 | ZINC000040548686 | -10.9 | -8.7 | -2.2 |
| 201 | ZINC000041546843 | -10.9 | -8.7 | -2.2 |
| **202** | **ZINC000043550595** | **-10.9** | **-8.7** | **-2.2** |
| 203 | ZINC000089827814 | -10.9 | -8.7 | -2.2 |
| 204 | ZINC000177842371 | -10.9 | -8.7 | -2.2 |
| 205 | ZINC000585093122 | -10.9 | -8.7 | -2.2 |
| 206 | ZINC000000390138 | -10.9 | -8.6 | -2.3 |
| 207 | ZINC000003292603 | -10.9 | -8.6 | -2.3 |
| **208** | **ZINC000240454699** | **-10.9** | **-8.6** | **-2.3** |
| 209 | ZINC000000079035 | -10.9 | -8.5 | -2.4 |
| **210** | **ZINC000044919578** | **-10.9** | **-8.5** | **-2.4** |
| 211 | ZINC000069846908 | -10.9 | -8.5 | -2.4 |
| 212 | ZINC000072096437 | -10.9 | -8.5 | -2.4 |
| 213 | ZINC000091304527 | -10.9 | -8.5 | -2.4 |
| 214 | ZINC000027755553 | -10.9 | -8.4 | -2.5 |
| **215** | **ZINC000008913866** | **-10.9** | **-8.3** | **-2.6** |
| 216 | ZINC000012867343 | -10.9 | -8.3 | -2.6 |
| 217 | ZINC000077353582 | -10.9 | -8.3 | -2.6 |
| 218 | ZINC000095415911 | -10.9 | -8.3 | -2.6 |
| 219 | ZINC000097105546 | -10.9 | -8.3 | -2.6 |
| 220 | ZINC000012508838 | -10.9 | -8.2 | -2.7 |
| 221 | ZINC000334003030 | -10.9 | -8.1 | -2.8 |
| 222 | ZINC000000326909 | -10.9 | -7.9 | -3.0 |

**Table S2.** Top-ranked selection of compounds based on their difference in the estimated binding affinity for IRAP versus ERAP1 (*Delta* < –2.5 kcal/mol), where *Delta* = $\Delta(\Delta G_{bind}^{(IRAP)} - \Delta G_{bind}^{(ERAP1)}$. Their ranking (#) is relative to the affinity for IRAP and follows that of **Table S1**. Entries marked in boldface indicate compounds that were finally selected for experimental screening.

| # | ZINC ID | VINA score (kcal/mol) | | |
|---|---|---|---|---|
| | | IRAP | ERAP1 | *Delta* |
| 223 | ZINC000008609997 | -10.8 | -8.3 | -2.5 |
| 224 | ZINC000097105494 | -10.8 | -8.3 | -2.5 |
| 225 | ZINC000013692677 | -10.8 | -8.2 | -2.6 |
| 226 | ZINC000023764785 | -10.8 | -8.2 | -2.6 |
| **227** | **ZINC000003463362** | **-10.8** | **-8.1** | **-2.7** |
| 228 | ZINC000257218603 | -10.8 | -7.9 | -2.9 |
| 229 | ZINC000091664087 | -10.8 | -7.4 | -3.4 |
| 230 | ZINC000000082954 | -10.7 | -8.2 | -2.5 |
| 231 | ZINC000002837442 | -10.7 | -8.2 | -2.5 |
| 232 | ZINC000005303591 | -10.7 | -8.2 | -2.5 |
| **233** | **ZINC000025032259** | **-10.7** | **-8.1** | **-2.6** |
| 234 | ZINC000100119126 | -10.7 | -8.0 | -2.7 |
| 235 | ZINC000012142779 | -10.7 | -7.9 | -2.8 |
| 236 | ZINC000012239509 | -10.7 | -7.9 | -2.8 |
| 237 | ZINC000065463577 | -10.7 | -7.9 | -2.8 |
| 238 | ZINC000050584912 | -10.7 | -7.6 | -3.1 |
| 239 | ZINC000040386093 | -10.7 | -7.5 | -3.2 |
| 240 | ZINC000092760184 | -10.7 | -7.5 | -3.2 |
| 241 | ZINC000252509448 | -10.7 | -7.4 | -3.3 |
| 242 | ZINC000004806959 | -10.6 | -8.1 | -2.5 |
| **243** | **ZINC000008595944** | **-10.6** | **-8.1** | **-2.5** |
| 244 | ZINC000040386114 | -10.6 | -8.1 | -2.5 |
| 245 | ZINC000215185750 | -10.6 | -8.1 | -2.5 |
| 246 | ZINC000253407066 | -10.6 | -8.1 | -2.5 |
| 247 | ZINC000575618196 | -10.6 | -8.1 | -2.5 |
| 248 | ZINC000096024249 | -10.6 | -8.0 | -2.6 |
| 249 | ZINC000021722970 | -10.6 | -7.9 | -2.7 |
| 250 | ZINC000067460397 | -10.6 | -7.9 | -2.7 |
| 251 | ZINC000096003964 | -10.6 | -7.9 | -2.7 |
| 252 | ZINC000306141880 | -10.6 | -7.4 | -3.2 |
| 253 | ZINC000100737122 | -10.6 | -7.1 | -3.5 |
| 254 | ZINC000095402766 | -10.6 | -6.9 | -3.7 |
| 255 | ZINC000014901170 | -10.5 | -8.0 | -2.5 |
| **256** | **ZINC000084578820** | **-10.5** | **-8.0** | **-2.5** |
| 257 | ZINC000274936458 | -10.5 | -8.0 | -2.5 |
| 258 | ZINC000096249561 | -10.5 | -7.9 | -2.6 |
| 259 | ZINC000001580185 | -10.5 | -7.7 | -2.8 |
| 260 | ZINC000004495846 | -10.5 | -7.6 | -2.9 |
| 261 | ZINC000257299555 | -10.5 | -7.6 | -2.9 |
| 262 | ZINC000012122382 | -10.5 | -7.5 | -3.0 |

| # | ZINC ID | VINA score (kcal/mol) | | |
|---|---|---|---|---|
| | | IRAP | ERAP1 | *Delta* |
| 263 | ZINC000075530913 | -10.5 | -7.3 | -3.2 |
| 264 | ZINC000040386098 | -10.4 | -7.9 | -2.5 |
| **265** | **ZINC000069562405** | **-10.4** | **-7.9** | **-2.5** |
| **266** | **ZINC000079491038** | **-10.4** | **-7.8** | **-2.6** |
| 267 | ZINC000006669352 | -10.4 | -7.7 | -2.7 |
| 268 | ZINC000012142783 | -10.4 | -7.7 | -2.7 |
| 269 | ZINC000238831563 | -10.4 | -7.7 | -2.7 |
| 270 | ZINC000253473237 | -10.4 | -7.7 | -2.7 |
| 271 | ZINC000257192562 | -10.4 | -7.7 | -2.7 |
| 272 | ZINC000238841632 | -10.4 | -7.6 | -2.8 |
| 273 | ZINC000257311417 | -10.4 | -7.6 | -2.8 |
| 274 | ZINC000095950700 | -10.4 | -7.5 | -2.9 |
| 275 | ZINC000306138347 | -10.4 | -7.5 | -2.9 |
| 276 | ZINC000081397352 | -10.4 | -7.4 | -3.0 |
| 277 | ZINC000096201851 | -10.4 | -7.4 | -3.0 |
| 278 | ZINC000278659999 | -10.4 | -7.4 | -3.0 |
| 279 | ZINC000092021781 | -10.4 | -7.3 | -3.1 |
| 280 | ZINC000091270544 | -10.4 | -7.1 | -3.3 |
| 281 | ZINC000005023816 | -10.3 | -7.8 | -2.5 |
| 282 | ZINC000012888207 | -10.3 | -7.8 | -2.5 |
| 283 | ZINC000014741626 | -10.3 | -7.8 | -2.5 |
| 284 | ZINC000061717590 | -10.3 | -7.8 | -2.5 |
| 285 | ZINC000069349055 | -10.3 | -7.8 | -2.5 |
| 286 | ZINC000170016387 | -10.3 | -7.8 | -2.5 |
| 287 | ZINC000584887110 | -10.3 | -7.8 | -2.5 |
| **288** | **ZINC000002216953** | **-10.3** | **-7.7** | **-2.6** |
| 289 | ZINC000006918427 | -10.3 | -7.7 | -2.6 |
| 290 | ZINC000065249112 | -10.3 | -7.7 | -2.6 |
| 291 | ZINC000065625052 | -10.3 | -7.7 | -2.6 |
| 292 | ZINC000075118384 | -10.3 | -7.7 | -2.6 |
| 293 | ZINC000119149039 | -10.3 | -7.7 | -2.6 |
| 294 | ZINC000065248686 | -10.3 | -7.6 | -2.7 |
| 295 | ZINC000065591272 | -10.3 | -7.6 | -2.7 |
| 296 | ZINC000005026046 | -10.3 | -7.5 | -2.8 |
| 297 | ZINC000058099587 | -10.3 | -7.3 | -3.0 |
| 298 | ZINC000154455008 | -10.3 | -7.3 | -3.0 |
| 299 | ZINC000091486946 | -10.3 | -7.2 | -3.1 |
| 300 | ZINC000170800517 | -10.3 | -7.1 | -3.2 |
| 301 | ZINC000000062112 | -10.2 | -7.7 | -2.5 |
| 302 | ZINC000005244779 | -10.2 | -7.7 | -2.5 |
| 303 | ZINC000006782318 | -10.2 | -7.7 | -2.5 |
| **304** | **ZINC000018148904** | **-10.2** | **-7.7** | **-2.5** |
| 305 | ZINC000069492530 | -10.2 | -7.7 | -2.5 |
| 306 | ZINC000079001413 | -10.2 | -7.7 | -2.5 |
| 307 | ZINC000000199600 | -10.2 | -7.6 | -2.6 |

| # | ZINC ID | VINA score (kcal/mol) | | |
|---|---|---|---|---|
| | | IRAP | ERAP1 | *Delta* |
| 308 | ZINC000003327891 | -10.2 | -7.6 | -2.6 |
| 309 | ZINC000004483817 | -10.2 | -7.6 | -2.6 |
| 310 | ZINC000042025678 | -10.2 | -7.6 | -2.6 |
| 311 | ZINC000057526383 | -10.2 | -7.6 | -2.6 |
| 312 | ZINC000065395709 | -10.2 | -7.6 | -2.6 |
| 313 | ZINC000096058349 | -10.2 | -7.6 | -2.6 |
| 314 | ZINC000257256038 | -10.2 | -7.6 | -2.6 |
| 315 | ZINC000006660103 | -10.2 | -7.5 | -2.7 |
| 316 | ZINC000057526528 | -10.2 | -7.5 | -2.7 |
| **317** | **ZINC000091486133** | **-10.2** | **-7.5** | **-2.7** |
| 318 | ZINC000097050589 | -10.2 | -7.5 | -2.7 |
| 319 | ZINC000330033487 | -10.2 | -7.5 | -2.7 |
| **320** | **ZINC000095410774** | **-10.2** | **-7.4** | **-2.8** |
| 321 | ZINC000096199614 | -10.2 | -7.4 | -2.8 |
| 322 | ZINC000000551535 | -10.2 | -7.2 | -3.0 |
| 323 | ZINC000096201858 | -10.2 | -6.9 | -3.3 |
| 324 | ZINC000002390471 | -10.1 | -7.6 | -2.5 |
| **325** | **ZINC000004511530** | **-10.1** | **-7.6** | **-2.5** |
| 326 | ZINC000005348603 | -10.1 | -7.6 | -2.5 |
| 327 | ZINC000012407997 | -10.1 | -7.6 | -2.5 |
| 328 | ZINC000096010936 | -10.1 | -7.6 | -2.5 |
| 329 | ZINC000097086417 | -10.1 | -7.6 | -2.5 |
| 330 | ZINC000195547314 | -10.1 | -7.6 | -2.5 |
| 331 | ZINC000215185695 | -10.1 | -7.6 | -2.5 |
| 332 | ZINC000225489282 | -10.1 | -7.6 | -2.5 |
| 333 | ZINC000237955843 | -10.1 | -7.6 | -2.5 |
| 334 | ZINC000588229346 | -10.1 | -7.6 | -2.5 |
| 335 | ZINC000057526426 | -10.1 | -7.5 | -2.6 |
| 336 | ZINC000069740630 | -10.1 | -7.5 | -2.6 |
| 337 | ZINC000071282106 | -10.1 | -7.5 | -2.6 |
| 338 | ZINC000092078469 | -10.1 | -7.4 | -2.7 |
| 339 | ZINC000096158052 | -10.1 | -7.4 | -2.7 |
| 340 | ZINC000096251711 | -10.1 | -7.4 | -2.7 |
| 341 | ZINC000000106944 | -10.1 | -7.3 | -2.8 |
| 342 | ZINC000092022499 | -10.1 | -7.3 | -2.8 |
| 343 | ZINC000334160460 | -10.1 | -7.3 | -2.8 |
| 344 | ZINC000091321614 | -10.1 | -7.2 | -2.9 |
| 345 | ZINC000096069677 | -10.1 | -7.2 | -2.9 |
| **346** | **ZINC000023642159** | **-10.1** | **-7.1** | **-3.0** |
| 347 | ZINC000096240774 | -10.1 | -7.1 | -3.0 |
| 348 | ZINC000001648546 | -10.1 | -7.0 | -3.1 |
| 349 | ZINC000001381807 | -10.0 | -7.5 | -2.5 |
| 350 | ZINC000095375482 | -10.0 | -7.5 | -2.5 |
| 351 | ZINC000192929211 | -10.0 | -7.5 | -2.5 |
| 352 | ZINC000229623928 | -10.0 | -7.5 | -2.5 |

| # | ZINC ID | VINA score (kcal/mol) | | |
|---|---|---|---|---|
| | | IRAP | ERAP1 | *Delta* |
| 353 | ZINC000253433258 | -10.0 | -7.5 | -2.5 |
| 354 | ZINC000000201272 | -10.0 | -7.4 | -2.6 |
| 355 | ZINC000020729822 | -10.0 | -7.4 | -2.6 |
| 356 | ZINC000057526442 | -10.0 | -7.4 | -2.6 |
| 357 | ZINC000065507679 | -10.0 | -7.4 | -2.6 |
| 358 | ZINC000096038482 | -10.0 | -7.4 | -2.6 |
| 359 | ZINC000096069669 | -10.0 | -7.4 | -2.6 |
| 360 | ZINC000100756927 | -10.0 | -7.4 | -2.6 |
| 361 | ZINC000237699092 | -10.0 | -7.4 | -2.6 |
| 362 | ZINC000238121810 | -10.0 | -7.4 | -2.6 |
| 363 | ZINC000064871452 | -10.0 | -7.3 | -2.7 |
| 364 | ZINC000092065698 | -10.0 | -7.3 | -2.7 |
| 365 | ZINC000092077873 | -10.0 | -7.3 | -2.7 |
| 366 | ZINC000245171655 | -10.0 | -7.3 | -2.7 |
| 367 | ZINC000071497775 | -10.0 | -7.0 | -3.0 |
| 368 | ZINC000018240833 | -9.9 | -7.4 | -2.5 |
| 369 | ZINC000022067509 | -9.9 | -7.4 | -2.5 |
| **370** | **ZINC000035869910** | **-9.9** | **-7.4** | **-2.5** |
| 371 | ZINC000040385915 | -9.9 | -7.4 | -2.5 |
| 372 | ZINC000055418850 | -9.9 | -7.4 | -2.5 |
| 373 | ZINC000065170071 | -9.9 | -7.4 | -2.5 |
| 374 | ZINC000069895275 | -9.9 | -7.4 | -2.5 |
| 375 | ZINC000071746202 | -9.9 | -7.4 | -2.5 |
| **376** | **ZINC000091270543** | **-9.9** | **-7.4** | **-2.5** |
| 377 | ZINC000091487017 | -9.9 | -7.4 | -2.5 |
| 378 | ZINC000095503625 | -9.9 | -7.4 | -2.5 |
| 379 | ZINC000237740654 | -9.9 | -7.4 | -2.5 |
| 380 | ZINC000238886413 | -9.9 | -7.4 | -2.5 |
| 381 | ZINC000409428381 | -9.9 | -7.4 | -2.5 |
| 382 | ZINC000000353960 | -9.9 | -7.3 | -2.6 |
| 383 | ZINC000000389450 | -9.9 | -7.3 | -2.6 |
| 384 | ZINC000002630701 | -9.9 | -7.3 | -2.6 |
| 385 | ZINC000005202436 | -9.9 | -7.3 | -2.6 |
| 386 | ZINC000057731402 | -9.9 | -7.3 | -2.6 |
| 387 | ZINC000072235028 | -9.9 | -7.3 | -2.6 |
| 388 | ZINC000095989182 | -9.9 | -7.3 | -2.6 |
| 389 | ZINC000169680567 | -9.9 | -7.3 | -2.6 |
| 390 | ZINC000019328346 | -9.9 | -7.2 | -2.7 |
| **391** | **ZINC000015078577** | **-9.9** | **-7.1** | **-2.8** |
| 392 | ZINC000006490389 | -9.9 | -7.0 | -2.9 |
| 393 | ZINC000096251709 | -9.9 | -7.0 | -2.9 |
| 394 | ZINC000216315534 | -9.9 | -6.7 | -3.2 |
| 395 | ZINC000005202682 | -9.8 | -7.3 | -2.5 |
| 396 | ZINC000029054164 | -9.8 | -7.3 | -2.5 |
| 397 | ZINC000069595091 | -9.8 | -7.3 | -2.5 |

| # | ZINC ID | VINA score (kcal/mol) | | |
|---|---|---|---|---|
| | | IRAP | ERAP1 | *Delta* |
| 398 | ZINC000072234903 | -9.8 | -7.3 | -2.5 |
| 399 | ZINC000075140034 | -9.8 | -7.3 | -2.5 |
| 400 | ZINC000091663451 | -9.8 | -7.3 | -2.5 |
| 401 | ZINC000096069697 | -9.8 | -7.3 | -2.5 |
| 402 | ZINC000107713021 | -9.8 | -7.3 | -2.5 |
| 403 | ZINC000092065700 | -9.8 | -7.2 | -2.6 |
| 404 | ZINC000096069207 | -9.8 | -7.2 | -2.6 |
| 405 | ZINC000096198984 | -9.8 | -7.2 | -2.6 |
| 406 | ZINC000257317306 | -9.8 | -7.2 | -2.6 |
| 407 | ZINC000328587242 | -9.8 | -7.2 | -2.6 |
| **408** | **ZINC000069737923** | **-9.8** | **-7.1** | **-2.7** |
| 409 | ZINC000238872716 | -9.8 | -7.1 | -2.7 |
| 410 | ZINC000003641308 | -9.7 | -7.2 | -2.5 |
| 411 | ZINC000040060202 | -9.7 | -7.2 | -2.5 |
| 412 | ZINC000040385901 | -9.7 | -7.2 | -2.5 |
| 413 | ZINC000096081777 | -9.7 | -7.2 | -2.5 |
| 414 | ZINC000585280336 | -9.7 | -7.2 | -2.5 |
| 415 | ZINC000002750765 | -9.7 | -7.1 | -2.6 |
| 416 | ZINC000096080454 | -9.7 | -7.1 | -2.6 |
| 417 | ZINC000096198954 | -9.7 | -7.1 | -2.6 |
| 418 | ZINC000097201269 | -9.7 | -7.1 | -2.6 |
| 419 | ZINC000121632943 | -9.7 | -7.0 | -2.7 |
| 420 | ZINC000257222272 | -9.7 | -7.0 | -2.7 |
| 421 | ZINC000071754931 | -9.7 | -6.9 | -2.8 |
| 422 | ZINC000091768990 | -9.7 | -6.9 | -2.8 |
| 423 | ZINC000257270100 | -9.7 | -6.8 | -2.9 |

**Table S3.** Energy decomposition of the MM/PBSA results for the 305 compounds using the *PB-1* parameter set (for more details see the Methods section). All energy terms are given in kcal/mol and are sorted by the binding free energy ($\Delta G_{bind}$), followed by the molecular mechanics van der Waals and electrostatic interactions terms ($\Delta E_{vdW}$ and $\Delta E_{el}$), the polar contribution to the solvation free energy ($\Delta G_{pol}$), the cavity formation and the van der Waals (dispersion) free energy terms that comprise the non-polar solvation free energy ($\Delta G_{np}$ and $\Delta G_{disp}$). Compounds marked in boldface were finally selected for experimental screening.

| # | ZINC ID | $\Delta G_{bind}$ | $\Delta E_{vdW}$ | $\Delta E_{el}$ | $\Delta G_{pol}$ | $\Delta G_{np}$ | $\Delta G_{disp}$ |
|---:|---|---:|---:|---:|---:|---:|---:|
| **1** | **ZINC000010256259** | **-15.7** | **-52.7** | **-41.4** | **55.5** | **-35.0** | **58.0** |
| **2** | **ZINC000035869910** | **-14.1** | **-54.6** | **-34.9** | **50.1** | **-36.8** | **62.1** |
| **3** | **ZINC000084578820** | **-13.9** | **-47.8** | **-35.2** | **44.3** | **-32.1** | **56.9** |
| 4 | ZINC000257317306 | -13.0 | -50.1 | -41.2 | 53.5 | -36.6 | 61.4 |
| 5 | ZINC000012888207 | -11.0 | -40.8 | -27.0 | 35.4 | -27.9 | 49.2 |
| **6** | **ZINC000015078577** | **-10.8** | **-49.4** | **-29.1** | **42.5** | **-34.7** | **59.9** |
| **7** | **ZINC000079491038** | **-10.5** | **-51.3** | **-27.7** | **45.5** | **-35.9** | **59.0** |
| **8** | **ZINC000084598177** | **-10.5** | **-49.0** | **-30.6** | **43.6** | **-32.1** | **57.6** |
| **9** | **ZINC000069562405** | **-9.8** | **-47.4** | **-25.3** | **37.8** | **-32.5** | **57.5** |
| **10** | **ZINC000005360885** | **-9.7** | **-48.1** | **-36.4** | **51.7** | **-31.8** | **55.0** |
| **11** | **ZINC000018148904** | **-9.5** | **-50.1** | **-32.7** | **50.6** | **-33.7** | **56.3** |
| **12** | **ZINC000022191233** | **-9.2** | **-53.6** | **-30.9** | **51.0** | **-36.6** | **61.0** |
| 13 | ZINC000096199614 | -9.1 | -51.3 | -27.5 | 46.5 | -32.0 | 55.1 |
| **14** | **ZINC000004511530** | **-8.6** | **-49.2** | **-22.6** | **42.0** | **-33.4** | **54.6** |
| 15 | ZINC000216315534 | -8.3 | -41.9 | -27.2 | 37.8 | -30.4 | 53.3 |
| **16** | **ZINC000000333210** | **-8.3** | **-50.6** | **-32.9** | **51.4** | **-33.1** | **56.9** |
| **17** | **ZINC000244816835** | **-8.1** | **-43.8** | **-34.1** | **47.4** | **-33.4** | **55.7** |
| **18** | **ZINC000044919578** | **-8.0** | **-43.0** | **-29.0** | **40.4** | **-29.6** | **53.2** |
| **19** | **ZINC000051467674** | **-8.0** | **-46.6** | **-40.6** | **55.1** | **-32.5** | **56.6** |
| **20** | **ZINC000091270543** | **-7.8** | **-53.6** | **-21.4** | **39.3** | **-36.1** | **64.0** |
| **21** | **ZINC000069737923** | **-7.7** | **-46.3** | **-29.6** | **42.9** | **-33.1** | **58.4** |
| **22** | **ZINC000223692657** | **-7.5** | **-50.8** | **-27.8** | **47.9** | **-33.1** | **56.3** |
| **23** | **ZINC000095410774** | **-7.4** | **-50.5** | **-22.8** | **42.7** | **-32.4** | **55.7** |
| **24** | **ZINC000003463362** | **-7.3** | **-50.0** | **-30.0** | **43.6** | **-34.3** | **63.4** |
| **25** | **ZINC000240454699** | **-7.0** | **-42.5** | **-24.0** | **35.5** | **-30.9** | **54.9** |
| 26 | ZINC000096058349 | -6.6 | -54.1 | -18.0 | 40.5 | -34.1 | 59.2 |
| **27** | **ZINC000095415912** | **-6.5** | **-53.2** | **-29.2** | **50.0** | **-34.5** | **60.4** |
| 28 | ZINC000067460397 | -6.4 | -50.1 | -35.5 | 55.7 | -31.9 | 55.4 |
| 29 | ZINC000013973188 | -6.2 | -47.2 | -29.6 | 47.3 | -31.7 | 54.9 |
| 30 | ZINC000014791001 | -6.0 | -47.0 | -21.3 | 39.9 | -32.3 | 54.6 |
| 31 | ZINC000084686490 | -5.9 | -46.1 | -33.1 | 50.5 | -33.3 | 56.1 |
| 32 | ZINC000334160460 | -5.9 | -49.1 | -22.2 | 40.1 | -33.4 | 58.7 |
| **33** | **ZINC000071915224** | **-5.8** | **-49.3** | **-26.0** | **43.6** | **-32.7** | **58.7** |
| 34 | ZINC000074014922 | -5.8 | -47.0 | -9.6 | 29.3 | -31.1 | 52.6 |
| 35 | ZINC000257299555 | -5.7 | -46.7 | -36.0 | 51.2 | -31.6 | 57.4 |
| 36 | ZINC000091270544 | -5.6 | -52.4 | -22.1 | 41.6 | -35.4 | 62.7 |
| 37 | ZINC000575618196 | -5.6 | -54.5 | -27.7 | 50.3 | -34.9 | 61.2 |

| # | ZINC ID | $\Delta G_{bind}$ | $\Delta E_{vdW}$ | $\Delta E_{el}$ | $\Delta G_{pol}$ | $\Delta G_{np}$ | $\Delta G_{disp}$ |
|---|---|---|---|---|---|---|---|
| 38 | ZINC000154455008 | -5.5 | -44.9 | -15.2 | 33.2 | -32.7 | 54.2 |
| 39 | ZINC000194368147 | -5.4 | -48.3 | -30.0 | 49.2 | -32.7 | 56.3 |
| 40 | ZINC000071754931 | -5.2 | -40.8 | -12.2 | 24.4 | -29.3 | 52.7 |
| **41** | **ZINC000043550595** | **-5.1** | **-48.3** | **-29.1** | **46.5** | **-33.6** | **59.4** |
| **42** | **ZINC000008595944** | **-5.0** | **-53.1** | **-13.9** | **37.5** | **-34.0** | **58.4** |
| **43** | **ZINC000069778215** | **-5.0** | **-47.6** | **-42.2** | **60.1** | **-33.8** | **58.5** |
| 44 | ZINC000100193430 | -5.0 | -47.9 | -13.6 | 33.4 | -30.3 | 53.4 |
| 45 | ZINC000003438521 | -4.9 | -45.4 | -22.7 | 39.2 | -30.9 | 54.8 |
| 46 | ZINC000075530913 | -4.8 | -49.2 | -20.2 | 37.5 | -34.1 | 61.2 |
| 47 | ZINC000225746732 | -4.8 | -47.5 | -20.8 | 38.7 | -30.9 | 55.6 |
| 48 | ZINC000003641308 | -4.7 | -45.0 | -25.7 | 44.0 | -31.2 | 53.1 |
| 49 | ZINC000018053795 | -4.7 | -47.5 | -22.6 | 42.2 | -31.9 | 55.1 |
| **50** | **ZINC000008913866** | **-4.7** | **-50.7** | **-28.3** | **48.7** | **-33.7** | **59.4** |
| **51** | **ZINC000057563200** | **-4.5** | **-49.9** | **-17.6** | **37.5** | **-33.9** | **59.3** |
| 52 | ZINC000044874685 | -4.4 | -44.2 | -22.4 | 37.9 | -32.2 | 56.5 |
| **53** | **ZINC000002216953** | **-4.4** | **-48.7** | **-24.8** | **43.1** | **-33.0** | **59.0** |
| 54 | ZINC000095469884 | -4.4 | -40.0 | -35.7 | 49.2 | -28.5 | 50.7 |
| 55 | ZINC000195547314 | -4.3 | -50.9 | -13.5 | 34.8 | -34.5 | 59.7 |
| 56 | ZINC000100193536 | -4.3 | -48.7 | -15.8 | 35.4 | -31.6 | 56.4 |
| 57 | ZINC000021835963 | -4.3 | -48.7 | -26.2 | 46.3 | -33.7 | 58.0 |
| 58 | ZINC000092078469 | -4.3 | -45.0 | -23.4 | 37.6 | -31.0 | 57.5 |
| 59 | ZINC000230113696 | -4.3 | -52.9 | -22.8 | 44.8 | -34.9 | 61.4 |
| 60 | ZINC000057731402 | -4.3 | -51.2 | -25.2 | 44.7 | -34.1 | 61.5 |
| 61 | ZINC000012142783 | -4.2 | -51.0 | -21.5 | 41.3 | -34.4 | 61.4 |
| 62 | ZINC000091768990 | -4.1 | -52.4 | -13.3 | 35.4 | -33.6 | 59.9 |
| 63 | ZINC000092065700 | -4.0 | -52.6 | -19.8 | 39.9 | -34.9 | 63.4 |
| 64 | ZINC000006669352 | -4.0 | -47.6 | -23.0 | 45.1 | -33.4 | 54.9 |
| 65 | ZINC000238121810 | -4.0 | -37.2 | -88.8 | 99.9 | -31.3 | 53.5 |
| 66 | ZINC000016543876 | -3.9 | -46.0 | -16.9 | 35.1 | -31.4 | 55.3 |
| 67 | ZINC000306141880 | -3.9 | -46.5 | -29.1 | 45.9 | -32.0 | 57.8 |
| **68** | **ZINC000004998502** | **-3.9** | **-53.5** | **-40.0** | **65.4** | **-35.7** | **59.9** |
| 69 | ZINC000095950700 | -3.9 | -48.8 | -19.4 | 37.5 | -32.0 | 58.9 |
| 70 | ZINC000096003964 | -3.9 | -49.1 | -26.2 | 49.3 | -32.8 | 55.0 |
| 71 | ZINC000009338249 | -3.8 | -47.0 | -27.4 | 46.6 | -30.6 | 54.7 |
| 72 | ZINC000585093122 | -3.2 | -45.2 | -16.8 | 34.3 | -30.8 | 55.2 |
| 73 | ZINC000091943530 | -3.1 | -44.1 | -16.2 | 33.7 | -31.4 | 55.0 |
| 74 | ZINC000092022499 | -3.1 | -53.4 | -23.1 | 45.3 | -35.3 | 63.3 |
| **75** | **ZINC000025032259** | **-3.0** | **-54.4** | **-21.9** | **48.0** | **-37.3** | **62.6** |
| 76 | ZINC000064871452 | -2.9 | -49.9 | -27.1 | 48.2 | -34.0 | 59.9 |
| 77 | ZINC000007030648 | -2.9 | -43.3 | -20.2 | 37.0 | -28.2 | 51.8 |
| 78 | ZINC000012323932 | -2.8 | -42.7 | -38.0 | 53.3 | -30.6 | 55.2 |
| 79 | ZINC000100119126 | -2.7 | -47.3 | -19.9 | 41.2 | -32.3 | 55.7 |
| 80 | ZINC000008738218 | -2.6 | -41.0 | -40.6 | 57.0 | -30.3 | 52.3 |
| 81 | ZINC000057526426 | -2.6 | -44.4 | -27.1 | 44.7 | -31.7 | 56.0 |
| 82 | ZINC000095951555 | -2.5 | -42.9 | -19.3 | 36.6 | -29.4 | 52.4 |
| 83 | ZINC000100191495 | -2.4 | -46.6 | -14.2 | 35.4 | -30.0 | 53.0 |

| # | ZINC ID | $\Delta G_{bind}$ | $\Delta E_{vdW}$ | $\Delta E_{el}$ | $\Delta G_{pol}$ | $\Delta G_{np}$ | $\Delta G_{disp}$ |
|---|---|---|---|---|---|---|---|
| 84 | ZINC000257211885 | -2.4 | -50.3 | -20.9 | 42.3 | -32.5 | 59.1 |
| 85 | ZINC000007030710 | -2.4 | -47.3 | -24.4 | 43.8 | -30.7 | 56.3 |
| 86 | ZINC000003313076 | -2.2 | -41.7 | -21.1 | 37.8 | -28.0 | 50.8 |
| 87 | ZINC000065248686 | -2.2 | -45.7 | -14.1 | 33.4 | -31.5 | 55.7 |
| 88 | ZINC000077503523 | -2.1 | -45.1 | -34.1 | 52.5 | -30.9 | 55.5 |
| 89 | ZINC000075140034 | -2.1 | -41.8 | -19.3 | 35.3 | -30.9 | 54.7 |
| 90 | ZINC000002630701 | -2.1 | -49.0 | -6.8 | 30.1 | -31.6 | 55.0 |
| 91 | ZINC000089827813 | -2.1 | -46.6 | -28.8 | 49.3 | -32.4 | 56.3 |
| 92 | ZINC000001381807 | -2.1 | -37.1 | -14.9 | 29.6 | -26.7 | 47.1 |
| 93 | ZINC000075118384 | -2.0 | -46.9 | -33.8 | 55.2 | -33.0 | 56.5 |
| 94 | ZINC000257192562 | -2.0 | -45.9 | -16.4 | 34.9 | -32.6 | 58.1 |
| 95 | ZINC000092760184 | -1.9 | -47.5 | -20.1 | 39.5 | -33.6 | 59.8 |
| 96 | ZINC000040473994 | -1.9 | -49.6 | -26.3 | 48.1 | -32.8 | 58.7 |
| 97 | ZINC000003292603 | -1.8 | -47.2 | -19.3 | 38.6 | -32.5 | 58.6 |
| 98 | ZINC000011781217 | -1.6 | -52.0 | -13.2 | 39.2 | -35.2 | 59.6 |
| 99 | ZINC000257206611 | -1.5 | -55.3 | -11.4 | 39.7 | -35.5 | 61.0 |
| 100 | ZINC000100737122 | -1.5 | -52.3 | -22.5 | 48.3 | -34.3 | 59.3 |
| 101 | ZINC000225489282 | -1.5 | -49.8 | -16.6 | 40.0 | -32.6 | 57.5 |
| 102 | ZINC000004722089 | -1.4 | -48.1 | -14.4 | 35.5 | -30.2 | 55.8 |
| 103 | ZINC000092021781 | -1.4 | -49.3 | -20.5 | 42.5 | -33.7 | 59.7 |
| 104 | ZINC000067643674 | -1.4 | -44.8 | -25.8 | 45.4 | -30.2 | 54.0 |
| **105** | **ZINC000091486133** | **-1.3** | **-52.7** | **-25.9** | **47.8** | **-34.7** | **64.2** |
| 106 | ZINC000008316344 | -1.2 | -51.0 | -4.6 | 28.5 | -33.0 | 58.9 |
| 107 | ZINC000069846908 | -1.2 | -50.0 | -33.8 | 58.0 | -34.1 | 58.8 |
| 108 | ZINC000019327176 | -1.1 | -40.2 | -26.8 | 42.1 | -27.8 | 51.5 |
| 109 | ZINC000035398834 | -1.1 | -51.5 | -9.7 | 35.6 | -33.1 | 57.5 |
| 110 | ZINC000095989182 | -1.0 | -43.2 | -18.7 | 38.4 | -28.9 | 51.4 |
| 111 | ZINC000584887110 | -1.0 | -46.9 | -14.2 | 35.2 | -32.1 | 56.9 |
| 112 | ZINC000097105546 | -1.0 | -44.7 | -22.4 | 44.6 | -30.0 | 51.5 |
| 113 | ZINC000096251711 | -0.9 | -50.9 | -15.0 | 39.6 | -33.2 | 58.7 |
| 114 | ZINC000229623928 | -0.9 | -50.6 | -34.1 | 58.3 | -33.9 | 59.4 |
| 115 | ZINC000096238948 | -0.9 | -53.8 | -13.0 | 38.8 | -34.3 | 61.4 |
| 116 | ZINC000171342968 | -0.7 | -48.4 | -39.2 | 62.0 | -33.5 | 58.5 |
| 117 | ZINC000096024249 | -0.7 | -47.4 | -18.1 | 41.7 | -29.3 | 52.5 |
| 118 | ZINC000050859891 | -0.6 | -48.4 | -29.5 | 53.8 | -31.1 | 54.4 |
| 119 | ZINC000072234903 | -0.6 | -44.1 | -12.5 | 34.2 | -30.6 | 52.5 |
| 120 | ZINC000257311417 | -0.5 | -40.7 | -23.4 | 40.3 | -29.5 | 52.8 |
| 121 | ZINC000014240188 | -0.5 | -45.7 | -17.2 | 39.1 | -31.1 | 54.4 |
| 122 | ZINC000278659999 | -0.4 | -47.8 | -23.7 | 46.2 | -33.0 | 57.9 |
| 123 | ZINC000257218603 | -0.4 | -50.2 | -24.7 | 45.8 | -35.0 | 63.8 |
| 124 | ZINC000022241454 | -0.3 | -39.1 | -38.4 | 51.9 | -26.7 | 51.9 |
| 125 | ZINC000002465084 | -0.3 | -49.8 | -23.7 | 49.9 | -32.8 | 56.2 |
| 126 | ZINC000095447759 | -0.3 | -46.9 | -24.8 | 47.3 | -30.8 | 54.9 |
| 127 | ZINC000069492530 | -0.1 | -47.5 | -19.0 | 43.1 | -31.1 | 54.4 |
| 128 | ZINC000119429896 | -0.1 | -49.5 | -9.9 | 33.6 | -33.8 | 59.6 |
| 129 | ZINC000000326909 | -0.1 | -42.9 | -17.0 | 37.1 | -27.4 | 50.1 |

| # | ZINC ID | $\Delta G_{bind}$ | $\Delta E_{vdW}$ | $\Delta E_{el}$ | $\Delta G_{pol}$ | $\Delta G_{np}$ | $\Delta G_{disp}$ |
|---|---|---|---|---|---|---|---|
| 130 | ZINC000257310083 | 0.0 | -47.2 | -16.6 | 37.1 | -31.2 | 57.8 |
| 131 | ZINC000096010936 | 0.0 | -41.4 | -26.7 | 45.4 | -31.7 | 54.3 |
| 132 | ZINC000010218408 | 0.0 | -47.3 | -19.2 | 42.9 | -29.7 | 53.2 |
| 133 | ZINC000077353582 | 0.0 | -48.6 | -15.4 | 40.5 | -30.7 | 54.4 |
| 134 | ZINC000091486946 | 0.1 | -52.4 | -21.3 | 44.0 | -34.8 | 64.5 |
| 135 | ZINC000002750765 | 0.1 | -45.0 | -24.4 | 44.7 | -31.5 | 56.2 |
| 136 | ZINC000135810445 | 0.1 | -44.5 | -31.9 | 52.9 | -30.9 | 54.5 |
| 137 | ZINC000069740630 | 0.1 | -38.0 | -20.4 | 36.1 | -28.9 | 51.4 |
| 138 | ZINC000177842371 | 0.1 | -45.3 | -26.9 | 48.9 | -28.9 | 52.3 |
| 139 | ZINC000096158052 | 0.2 | -49.9 | -20.8 | 43.2 | -34.2 | 61.9 |
| 140 | ZINC000040060202 | 0.2 | -46.1 | -17.7 | 39.3 | -33.7 | 58.4 |
| 141 | ZINC000253433259 | 0.3 | -49.1 | -19.7 | 44.2 | -31.4 | 56.3 |
| **142** | **ZINC000023642159** | **0.3** | **-47.6** | **-27.3** | **48.4** | **-32.0** | **58.8** |
| 143 | ZINC000095393415 | 0.4 | -54.8 | -14.5 | 42.4 | -32.4 | 59.7 |
| 144 | ZINC000257222272 | 0.5 | -51.4 | -7.3 | 33.9 | -34.8 | 60.1 |
| 145 | ZINC000328587242 | 0.5 | -45.2 | -23.7 | 42.3 | -34.1 | 61.2 |
| 146 | ZINC000002646979 | 0.5 | -51.5 | -22.3 | 47.4 | -32.2 | 59.1 |
| 147 | ZINC000238886413 | 0.5 | -49.4 | -11.6 | 37.1 | -32.3 | 56.7 |
| 148 | ZINC000097201269 | 0.7 | -49.4 | -16.9 | 41.4 | -35.5 | 61.2 |
| 149 | ZINC000065395709 | 0.7 | -51.6 | -22.3 | 50.7 | -35.3 | 59.1 |
| 150 | ZINC000006117573 | 0.8 | -42.2 | -28.0 | 47.0 | -30.2 | 54.3 |
| 151 | ZINC000016228943 | 0.8 | -43.6 | -26.3 | 45.9 | -29.4 | 54.3 |
| 152 | ZINC000040548686 | 0.9 | -51.5 | -23.2 | 49.8 | -32.4 | 58.2 |
| 153 | ZINC000042025678 | 1.0 | -43.6 | -12.3 | 33.3 | -29.1 | 52.8 |
| 154 | ZINC000192753414 | 1.1 | -46.9 | -32.1 | 56.3 | -31.0 | 54.8 |
| 155 | ZINC000069349055 | 1.1 | -48.3 | -24.2 | 47.9 | -34.0 | 59.7 |
| 156 | ZINC000058099587 | 1.2 | -46.8 | -18.8 | 42.1 | -31.9 | 56.6 |
| 157 | ZINC000004535480 | 1.2 | -45.6 | -15.4 | 38.6 | -28.7 | 52.3 |
| 158 | ZINC000005426253 | 1.2 | -49.8 | -11.6 | 36.2 | -34.6 | 61.0 |
| 159 | ZINC000050857093 | 1.2 | -48.7 | -25.4 | 51.1 | -32.3 | 56.6 |
| 160 | ZINC000178511392 | 1.3 | -40.2 | -33.5 | 52.3 | -29.5 | 52.1 |
| 161 | ZINC000005026046 | 1.3 | -45.8 | -14.0 | 35.2 | -30.4 | 56.4 |
| 162 | ZINC000095503625 | 1.4 | -50.6 | -24.3 | 51.5 | -34.5 | 59.2 |
| 163 | ZINC000035671101 | 1.4 | -46.7 | -8.2 | 30.9 | -29.8 | 55.1 |
| 164 | ZINC000069775761 | 1.5 | -48.1 | -21.8 | 45.0 | -32.2 | 58.6 |
| 165 | ZINC000097413355 | 1.6 | -46.7 | -27.5 | 47.9 | -30.7 | 58.6 |
| 166 | ZINC000013691850 | 1.6 | -49.9 | -15.3 | 42.3 | -31.2 | 55.6 |
| 167 | ZINC000585280336 | 1.6 | -51.7 | -30.1 | 57.6 | -32.2 | 58.1 |
| 168 | ZINC000050584912 | 1.6 | -50.3 | -6.3 | 32.9 | -33.4 | 58.7 |
| 169 | ZINC000069595091 | 1.8 | -45.8 | -22.4 | 46.0 | -32.7 | 56.6 |
| 170 | ZINC000022549345 | 1.8 | -53.9 | -18.2 | 47.5 | -34.7 | 61.0 |
| 171 | ZINC000409428381 | 1.8 | -51.5 | -12.2 | 39.2 | -35.9 | 62.2 |
| 172 | ZINC000072096437 | 1.8 | -50.9 | -18.3 | 45.9 | -35.2 | 60.3 |
| 173 | ZINC000065463577 | 1.8 | -46.6 | -32.2 | 55.7 | -31.1 | 56.0 |
| 174 | ZINC000072277544 | 1.8 | -45.7 | -15.0 | 37.7 | -29.9 | 54.8 |
| 175 | ZINC000072300733 | 1.9 | -51.3 | -12.8 | 40.6 | -33.3 | 58.7 |

| # | ZINC ID | $\Delta G_{bind}$ | $\Delta E_{vdW}$ | $\Delta E_{el}$ | $\Delta G_{pol}$ | $\Delta G_{np}$ | $\Delta G_{disp}$ |
|---|---|---|---|---|---|---|---|
| 176 | ZINC000052955795 | 2.0 | -46.7 | -27.1 | 49.1 | -33.1 | 59.8 |
| 177 | ZINC000012550491 | 2.2 | -46.4 | -23.5 | 47.0 | -31.1 | 56.3 |
| 178 | ZINC000003852421 | 2.2 | -44.3 | -12.3 | 35.0 | -28.4 | 52.1 |
| 179 | ZINC000001810957 | 2.2 | -45.8 | -17.2 | 41.9 | -31.0 | 54.3 |
| 180 | ZINC000091304527 | 2.2 | -50.9 | -14.0 | 40.6 | -34.3 | 60.8 |
| 181 | ZINC000040386093 | 2.3 | -45.0 | -13.8 | 38.2 | -31.4 | 54.3 |
| 182 | ZINC000072290753 | 2.3 | -52.3 | -26.0 | 53.7 | -32.4 | 59.4 |
| 183 | ZINC000057771728 | 2.3 | -48.2 | -24.0 | 49.7 | -33.0 | 57.7 |
| 184 | ZINC000005303591 | 2.5 | -44.2 | -20.2 | 42.9 | -31.2 | 55.1 |
| 185 | ZINC000034895992 | 2.5 | -49.7 | -29.9 | 56.3 | -35.0 | 60.9 |
| 186 | ZINC000072290711 | 2.5 | -46.7 | -16.6 | 39.1 | -29.4 | 56.2 |
| 187 | ZINC000040386098 | 2.6 | -42.7 | -13.8 | 36.9 | -30.0 | 52.0 |
| 188 | ZINC000014741626 | 2.6 | -49.6 | -6.8 | 33.0 | -33.4 | 59.5 |
| 189 | ZINC000306138347 | 2.7 | -49.7 | -17.2 | 44.5 | -32.0 | 57.1 |
| 190 | ZINC000257256038 | 2.7 | -45.9 | -24.2 | 47.3 | -32.2 | 57.7 |
| 191 | ZINC000013691849 | 2.8 | -44.2 | -33.9 | 56.2 | -31.2 | 55.9 |
| 192 | ZINC000079001413 | 2.8 | -52.9 | -9.1 | 38.0 | -33.8 | 60.6 |
| 193 | ZINC000002922856 | 2.8 | -52.4 | -15.5 | 46.1 | -33.4 | 58.1 |
| 194 | ZINC000023764785 | 2.8 | -45.9 | -15.6 | 40.0 | -32.1 | 56.5 |
| 195 | ZINC000013692677 | 3.2 | -51.9 | -30.8 | 59.8 | -34.6 | 60.6 |
| 196 | ZINC000029134571 | 3.2 | -52.1 | -19.6 | 50.3 | -33.8 | 58.4 |
| 197 | ZINC000040385901 | 3.2 | -41.1 | -13.0 | 36.0 | -28.8 | 50.1 |
| 198 | ZINC000096122792 | 3.3 | -48.6 | -9.2 | 37.6 | -31.0 | 54.4 |
| 199 | ZINC000008776545 | 3.3 | -47.3 | -15.4 | 40.4 | -31.0 | 56.6 |
| 200 | ZINC000072290626 | 3.3 | -48.7 | -19.4 | 45.1 | -31.5 | 57.8 |
| 201 | ZINC000096251709 | 3.4 | -48.0 | -18.9 | 44.7 | -32.0 | 57.6 |
| 202 | ZINC000028790813 | 3.4 | -51.7 | -24.1 | 52.5 | -33.4 | 60.1 |
| 203 | ZINC000096038482 | 3.4 | -39.5 | -24.6 | 43.4 | -29.8 | 53.9 |
| 204 | ZINC000044908085 | 3.4 | -51.1 | -21.0 | 48.6 | -31.9 | 58.9 |
| 205 | ZINC000170800517 | 3.5 | -49.4 | -18.5 | 44.9 | -34.0 | 60.6 |
| 206 | ZINC000091664087 | 3.6 | -54.4 | -7.8 | 39.1 | -34.2 | 60.9 |
| 207 | ZINC000085879141 | 3.6 | -41.7 | -10.6 | 32.2 | -29.1 | 52.8 |
| 208 | ZINC000257257831 | 3.7 | -45.8 | -12.3 | 38.3 | -30.3 | 53.8 |
| 209 | ZINC000072437791 | 3.8 | -46.5 | -12.5 | 38.1 | -30.8 | 55.4 |
| 210 | ZINC000095402766 | 3.9 | -44.9 | -16.8 | 40.7 | -32.2 | 57.0 |
| 211 | ZINC000096837115 | 3.9 | -45.6 | -27.4 | 50.7 | -31.5 | 57.8 |
| 212 | ZINC000003327891 | 4.0 | -45.9 | -11.8 | 36.6 | -31.1 | 56.3 |
| 213 | ZINC000019973951 | 4.1 | -40.3 | -29.4 | 48.2 | -28.2 | 53.8 |
| 214 | ZINC000006782318 | 4.1 | -47.7 | -18.1 | 43.9 | -31.1 | 57.1 |
| 215 | ZINC000096080454 | 4.2 | -44.4 | -19.6 | 44.1 | -29.5 | 53.6 |
| 216 | ZINC000005088834 | 4.3 | -42.9 | -7.6 | 31.3 | -30.3 | 53.7 |
| 217 | ZINC000245171655 | 4.3 | -48.7 | -10.2 | 39.1 | -32.6 | 56.7 |
| 218 | ZINC000097050589 | 4.4 | -48.8 | -13.2 | 42.6 | -32.2 | 55.9 |
| 219 | ZINC000012508838 | 4.4 | -49.0 | -24.8 | 54.5 | -33.3 | 57.0 |
| 220 | ZINC000065167646 | 4.4 | -47.8 | -21.8 | 49.2 | -32.1 | 56.9 |
| 221 | ZINC000253407066 | 4.5 | -46.7 | -13.8 | 41.2 | -31.7 | 55.4 |

| # | ZINC ID | $\Delta G_{bind}$ | $\Delta E_{vdW}$ | $\Delta E_{el}$ | $\Delta G_{pol}$ | $\Delta G_{np}$ | $\Delta G_{disp}$ |
|---|---|---|---|---|---|---|---|
| 222 | ZINC000071746202 | 4.6 | -47.6 | -15.1 | 39.6 | -31.9 | 59.6 |
| 223 | ZINC000257270100 | 4.6 | -46.1 | -10.6 | 37.3 | -31.8 | 55.8 |
| 224 | ZINC000071282106 | 4.7 | -47.7 | -15.4 | 40.9 | -33.0 | 59.9 |
| 225 | ZINC000008771448 | 4.7 | -48.5 | -23.5 | 51.6 | -32.9 | 58.1 |
| 226 | ZINC000000489138 | 4.8 | -50.3 | -20.6 | 51.6 | -32.3 | 56.3 |
| 227 | ZINC000153353600 | 4.9 | -46.2 | -8.9 | 35.1 | -31.7 | 56.7 |
| 228 | ZINC000003875380 | 4.9 | -46.6 | -15.5 | 39.6 | -28.6 | 56.1 |
| 229 | ZINC000065591272 | 5.3 | -44.9 | -9.7 | 35.0 | -31.5 | 56.3 |
| 230 | ZINC000019328346 | 5.4 | -46.7 | -22.0 | 48.9 | -31.6 | 56.9 |
| 231 | ZINC000072896859 | 5.4 | -48.3 | -6.8 | 34.3 | -32.6 | 58.9 |
| 232 | ZINC000057511709 | 5.4 | -45.5 | -22.5 | 49.1 | -30.8 | 55.1 |
| 233 | ZINC000034407910 | 5.5 | -38.4 | -15.1 | 36.8 | -26.9 | 49.1 |
| 234 | ZINC000062639858 | 5.6 | -52.0 | -3.0 | 33.8 | -34.7 | 61.5 |
| 235 | ZINC000299793701 | 5.8 | -42.4 | -24.9 | 47.1 | -30.1 | 56.1 |
| 236 | ZINC000091321614 | 5.9 | -49.4 | -18.7 | 47.5 | -33.2 | 59.7 |
| 237 | ZINC000030875068 | 6.0 | -45.9 | -10.0 | 37.4 | -30.4 | 54.8 |
| 238 | ZINC000071497775 | 6.0 | -51.3 | -25.4 | 56.4 | -34.5 | 60.9 |
| 239 | ZINC000072807915 | 6.0 | -50.6 | -7.2 | 36.8 | -33.9 | 60.9 |
| 240 | ZINC000012407997 | 6.3 | -48.0 | -11.5 | 40.0 | -33.1 | 59.0 |
| 241 | ZINC000006887927 | 6.3 | -48.4 | -23.8 | 52.3 | -33.9 | 60.0 |
| 242 | ZINC000584897275 | 6.4 | -44.7 | -36.7 | 60.9 | -32.1 | 59.0 |
| 243 | ZINC000253473237 | 6.5 | -44.9 | -8.3 | 35.6 | -31.5 | 55.7 |
| 244 | ZINC000096069207 | 6.6 | -52.2 | -20.5 | 51.6 | -33.3 | 60.9 |
| 245 | ZINC000027755553 | 6.7 | -46.7 | -15.5 | 44.1 | -31.2 | 56.1 |
| 246 | ZINC000048249797 | 6.7 | -50.8 | -10.3 | 42.1 | -33.8 | 59.5 |
| 247 | ZINC000330033487 | 6.8 | -50.1 | -15.9 | 47.2 | -33.3 | 58.9 |
| 248 | ZINC000002390471 | 6.8 | -43.9 | -12.4 | 37.7 | -29.4 | 54.8 |
| 249 | ZINC000084563579 | 6.9 | -45.4 | -20.3 | 49.7 | -33.4 | 56.3 |
| 250 | ZINC000047209496 | 6.9 | -44.4 | -13.3 | 40.4 | -31.5 | 55.7 |
| 251 | ZINC000255187536 | 6.9 | -42.3 | -21.3 | 46.6 | -29.4 | 53.4 |
| 252 | ZINC000072235028 | 7.0 | -52.8 | -12.4 | 45.9 | -34.1 | 60.4 |
| 253 | ZINC000004722011 | 7.2 | -44.0 | -7.1 | 33.8 | -29.5 | 54.0 |
| 254 | ZINC000096069697 | 7.7 | -45.5 | -9.3 | 36.2 | -30.7 | 56.9 |
| 255 | ZINC000005348603 | 7.8 | -46.3 | -8.7 | 39.0 | -31.3 | 55.1 |
| 256 | ZINC000239762559 | 8.1 | -48.8 | -21.2 | 52.5 | -32.5 | 58.1 |
| 257 | ZINC000022067509 | 8.1 | -49.1 | -30.2 | 62.4 | -34.0 | 59.0 |
| 258 | ZINC000018240833 | 8.1 | -47.5 | -37.8 | 67.0 | -32.9 | 59.2 |
| 259 | ZINC000192929211 | 8.4 | -44.2 | -24.0 | 52.8 | -31.7 | 55.6 |
| 260 | ZINC000040266307 | 8.7 | -44.5 | -22.2 | 50.7 | -31.2 | 55.9 |
| 261 | ZINC000077522487 | 8.8 | -48.6 | -28.5 | 58.3 | -33.0 | 60.4 |
| 262 | ZINC000017888288 | 9.0 | -44.1 | -16.9 | 45.5 | -28.4 | 52.9 |
| 263 | ZINC000170016387 | 9.1 | -42.6 | -20.3 | 47.9 | -31.4 | 55.6 |
| 264 | ZINC000068269627 | 9.3 | -50.4 | -27.6 | 60.1 | -33.1 | 60.3 |
| 265 | ZINC000004732939 | 9.3 | -43.8 | -12.5 | 42.8 | -31.5 | 54.4 |
| 266 | ZINC000006556210 | 9.3 | -41.4 | -11.3 | 39.4 | -28.3 | 50.9 |
| 267 | ZINC000136345745 | 9.4 | -51.8 | -19.5 | 55.0 | -33.3 | 59.0 |

| # | ZINC ID | $\Delta G_{bind}$ | $\Delta E_{vdW}$ | $\Delta E_{el}$ | $\Delta G_{pol}$ | $\Delta G_{np}$ | $\Delta G_{disp}$ |
|---|---|---|---|---|---|---|---|
| 268 | ZINC000000079035 | 9.6 | -47.2 | -19.1 | 50.2 | -32.7 | 58.5 |
| 269 | ZINC000091487017 | 9.7 | -49.3 | -11.4 | 43.6 | -33.4 | 60.3 |
| 270 | ZINC000238831563 | 9.8 | -47.8 | -11.4 | 45.2 | -31.1 | 55.0 |
| 271 | ZINC000002837442 | 9.8 | -42.9 | -16.1 | 43.4 | -30.1 | 55.5 |
| 272 | ZINC000237740654 | 10.0 | -45.0 | -3.6 | 32.9 | -31.7 | 57.5 |
| 273 | ZINC000000353960 | 10.1 | -35.6 | -38.0 | 65.4 | -27.4 | 45.6 |
| 274 | ZINC000253433605 | 10.1 | -47.3 | -14.5 | 47.9 | -32.7 | 56.6 |
| 275 | ZINC000008609997 | 10.1 | -50.6 | -13.5 | 48.4 | -32.9 | 58.7 |
| 276 | ZINC000000201272 | 10.3 | -46.5 | -7.2 | 39.9 | -30.2 | 54.3 |
| 277 | ZINC000061067795 | 10.6 | -48.3 | -13.8 | 47.4 | -32.7 | 58.0 |
| 278 | ZINC000004495846 | 10.7 | -46.3 | -26.6 | 56.9 | -31.0 | 57.6 |
| 279 | ZINC000107713021 | 11.0 | -44.7 | -2.8 | 32.5 | -31.7 | 57.7 |
| 280 | ZINC000019796838 | 11.1 | -46.3 | -11.2 | 42.9 | -30.7 | 56.3 |
| 281 | ZINC000089857747 | 11.1 | -50.7 | -7.6 | 44.1 | -32.6 | 57.9 |
| 282 | ZINC000001620500 | 11.5 | -42.1 | -26.7 | 56.1 | -29.8 | 53.9 |
| 283 | ZINC000009318781 | 11.6 | -46.3 | -25.9 | 60.2 | -30.0 | 53.6 |
| 284 | ZINC000268943077 | 11.6 | -41.8 | 6.5 | 22.8 | -29.3 | 53.4 |
| 285 | ZINC000012557330 | 12.3 | -43.0 | -10.6 | 40.7 | -27.7 | 52.8 |
| 286 | ZINC000006918427 | 12.3 | -45.0 | -13.2 | 45.8 | -30.0 | 54.7 |
| 287 | ZINC000012142779 | 12.9 | -50.2 | -15.4 | 52.2 | -34.2 | 60.5 |
| 288 | ZINC000215185695 | 13.1 | -47.4 | -16.7 | 51.7 | -34.3 | 59.9 |
| 289 | ZINC000072432742 | 13.3 | -49.1 | 1.8 | 35.2 | -33.3 | 58.6 |
| 290 | ZINC000005244779 | 13.5 | -49.8 | -9.7 | 47.4 | -33.1 | 58.6 |
| 291 | ZINC000284798034 | 13.6 | -50.9 | -22.8 | 62.0 | -32.4 | 57.7 |
| 292 | ZINC000012239509 | 13.9 | -50.5 | -7.5 | 45.9 | -33.8 | 59.8 |
| 293 | ZINC000012122382 | 14.2 | -42.6 | -9.7 | 40.2 | -30.1 | 56.5 |
| 294 | ZINC000013278475 | 14.4 | -48.6 | -19.5 | 59.8 | -32.5 | 55.2 |
| 295 | ZINC000575617533 | 14.4 | -44.8 | -10.3 | 42.4 | -31.4 | 58.4 |
| 296 | ZINC000004807849 | 14.7 | -36.5 | -47.4 | 74.5 | -26.7 | 50.9 |
| 297 | ZINC000237955843 | 16.3 | -40.6 | -42.8 | 73.6 | -30.5 | 56.6 |
| 298 | ZINC000018177963 | 16.7 | -32.7 | -55.9 | 82.0 | -25.9 | 49.2 |
| 299 | ZINC000006576136 | 17.2 | -34.5 | -6.9 | 37.6 | -26.5 | 47.5 |
| 300 | ZINC000069895275 | 18.4 | -37.0 | -10.2 | 43.9 | -27.3 | 49.0 |
| 301 | ZINC000004806959 | 18.7 | -39.4 | -29.2 | 63.2 | -29.0 | 53.1 |
| 302 | ZINC000237699092 | 21.8 | -40.8 | -8.7 | 45.0 | -29.6 | 55.9 |
| 303 | ZINC000008848147 | 21.9 | -38.7 | -0.8 | 39.0 | -27.4 | 49.9 |
| 304 | ZINC000003947438 | 22.2 | -41.0 | -21.6 | 59.8 | -29.4 | 54.5 |
| 305 | ZINC000096198984 | 24.0 | -45.9 | -8.9 | 52.1 | -32.0 | 58.7 |

**Table S4.** Energy decomposition of the MM/PBSA results for the 305 compounds using the *PB-4* parameter set (for more details see the Methods section). All energy terms are given in kcal/mol with their standard deviation (*std*) and are sorted by the binding free energy ($\Delta G_{bind}$) followed by the molecular mechanics van der Waals and electrostatic interactions terms ($\Delta E_{vdW}$ and $\Delta E_{el}$) and the polar and non-polar contributions to the solvation free energy ($\Delta G_{pol}$ and $\Delta G_{np}$). Compounds marked in boldface were finally selected for experimental screening.

| # | ZINC ID | $\Delta G_{bind}$ | *std* | $\Delta E_{vdW}$ | *std* | $\Delta E_{el}$ | *std* | $\Delta G_{pol}$ | *std* | $\Delta G_{np}$ | *std* |
|---|---|---|---|---|---|---|---|---|---|---|---|
| **1** | **ZINC000035869910** | **-38.7** | **4.4** | **-54.6** | **2.6** | **-35.5** | **5.1** | **57.7** | **3.7** | **-6.3** | **0.1** |
| **2** | **ZINC000003463362** | **-37.7** | **3.3** | **-50.0** | **2.8** | **-30.9** | **4.2** | **49.5** | **4.1** | **-6.3** | **0.2** |
| **3** | **ZINC000091270543** | **-36.9** | **3.4** | **-53.6** | **3.0** | **-23.0** | **3.8** | **46.0** | **3.0** | **-6.2** | **0.1** |
| **4** | **ZINC000084578820** | **-36.3** | **4.2** | **-47.8** | **2.6** | **-35.8** | **7.0** | **53.2** | **4.0** | **-5.8** | **0.1** |
| 5 | ZINC000257317306 | -36.2 | 4.5 | -50.1 | 2.8 | -41.5 | 3.3 | 62.1 | 4.2 | -6.7 | 0.1 |
| **6** | **ZINC000084598177** | **-36.2** | **3.5** | **-49.0** | **2.5** | **-29.0** | **3.2** | **47.6** | **3.7** | **-5.8** | **0.1** |
| **7** | **ZINC000005360885** | **-36.0** | **4.2** | **-48.1** | **2.7** | **-36.1** | **4.9** | **53.9** | **2.7** | **-5.7** | **0.1** |
| **8** | **ZINC000095415912** | **-35.4** | **3.9** | **-53.2** | **2.7** | **-28.4** | **4.2** | **52.1** | **3.0** | **-6.0** | **0.1** |
| **9** | **ZINC000057563200** | **-35.4** | **4.4** | **-49.9** | **2.7** | **-17.6** | **4.6** | **38.4** | **3.2** | **-6.4** | **0.1** |
| **10** | **ZINC000022191233** | **-35.1** | **3.5** | **-53.6** | **2.9** | **-31.4** | **3.7** | **56.0** | **3.1** | **-6.1** | **0.1** |
| 11 | ZINC000092065700 | -34.4 | 3.7 | -52.6 | 2.5 | -19.7 | 2.9 | 44.5 | 3.6 | -6.5 | 0.1 |
| **12** | **ZINC000079491038** | **-34.4** | **4.3** | **-51.3** | **2.7** | **-28.2** | **5.1** | **51.2** | **4.7** | **-6.1** | **0.2** |
| **13** | **ZINC000010256259** | **-34.3** | **3.8** | **-52.7** | **2.7** | **-42.1** | **3.7** | **66.5** | **3.7** | **-6.0** | **0.1** |
| **14** | **ZINC000069562405** | **-33.7** | **3.1** | **-47.4** | **2.6** | **-27.1** | **2.9** | **46.6** | **2.4** | **-5.8** | **0.1** |
| **15** | **ZINC000000333210** | **-33.6** | **3.2** | **-50.6** | **3.0** | **-32.6** | **3.7** | **55.3** | **3.0** | **-5.8** | **0.1** |
| 16 | ZINC000084686490 | -33.6 | 3.6 | -46.1 | 2.4 | -33.4 | 3.0 | 51.8 | 3.1 | -6.0 | 0.1 |
| **17** | **ZINC000223692657** | **-33.5** | **3.3** | **-50.8** | **2.6** | **-28.2** | **3.9** | **51.2** | **2.7** | **-5.7** | **0.1** |
| **18** | **ZINC000018148904** | **-33.4** | **3.5** | **-50.1** | **2.6** | **-32.4** | **3.5** | **54.7** | **2.7** | **-5.7** | **0.1** |
| 19 | ZINC000096199614 | -33.3 | 3.7 | -51.3 | 2.5 | -28.0 | 3.7 | 51.5 | 2.7 | -5.5 | 0.1 |
| **20** | **ZINC000069737923** | **-33.2** | **3.3** | **-46.3** | **2.3** | **-30.1** | **4.0** | **49.5** | **3.1** | **-6.3** | **0.1** |
| 21 | ZINC000096058349 | -33.1 | 3.8 | -54.1 | 2.4 | -18.0 | 2.3 | 44.9 | 2.7 | -5.8 | 0.1 |
| **22** | **ZINC000043550595** | **-33.1** | **4.0** | **-48.3** | **2.5** | **-29.1** | **3.4** | **50.3** | **3.0** | **-6.1** | **0.1** |
| 23 | ZINC000092078469 | -33.0 | 3.9 | -45.0 | 3.2 | -24.1 | 3.6 | 42.2 | 3.6 | -6.2 | 0.2 |
| 24 | ZINC000092022499 | -33.0 | 3.5 | -53.4 | 2.6 | -23.3 | 2.6 | 50.0 | 3.4 | -6.3 | 0.1 |
| **25** | **ZINC000071915224** | **-32.9** | **3.7** | **-49.3** | **2.3** | **-26.5** | **3.5** | **48.7** | **3.4** | **-5.9** | **0.1** |
| 26 | ZINC000095950700 | -32.9 | 3.2 | -48.8 | 2.7 | -19.6 | 2.1 | 41.3 | 2.9 | -5.8 | 0.1 |
| 27 | ZINC000100193536 | -32.8 | 3.6 | -48.7 | 2.9 | -15.8 | 2.9 | 37.6 | 2.7 | -5.9 | 0.1 |
| 28 | ZINC000091270544 | -32.7 | 3.8 | -52.4 | 2.7 | -22.2 | 3.8 | 48.2 | 3.5 | -6.3 | 0.1 |
| **29** | **ZINC000015078577** | **-32.6** | **3.8** | **-49.4** | **2.8** | **-29.5** | **4.6** | **52.5** | **4.6** | **-6.2** | **0.1** |
| **30** | **ZINC000051467674** | **-32.6** | **3.6** | **-46.6** | **2.7** | **-40.1** | **3.8** | **60.0** | **3.3** | **-5.9** | **0.1** |
| **31** | **ZINC000095410774** | **-32.5** | **3.2** | **-50.5** | **2.4** | **-24.2** | **3.1** | **47.7** | **2.7** | **-5.5** | **0.1** |
| **32** | **ZINC000240454699** | **-32.2** | **3.2** | **-42.5** | **2.5** | **-25.1** | **4.6** | **41.3** | **3.5** | **-5.9** | **0.1** |
| 33 | ZINC000216315534 | -32.1 | 2.7 | -41.9 | 2.5 | -28.6 | 4.7 | 44.1 | 3.7 | -5.7 | 0.1 |
| 34 | ZINC000089827813 | -32.1 | 4.1 | -46.6 | 3.2 | -29.5 | 4.7 | 49.7 | 2.6 | -5.6 | 0.1 |
| 35 | ZINC000225746732 | -32.0 | 2.6 | -47.5 | 2.3 | -21.3 | 2.3 | 42.2 | 2.3 | -5.5 | 0.1 |
| 36 | ZINC000004722089 | -32.0 | 3.1 | -48.1 | 2.5 | -14.7 | 2.7 | 36.5 | 2.6 | -5.7 | 0.1 |
| 37 | ZINC000195547314 | -31.9 | 3.2 | -50.9 | 2.8 | -14.0 | 3.2 | 39.2 | 2.7 | -6.1 | 0.1 |
| 38 | ZINC000334160460 | -31.8 | 3.3 | -49.1 | 2.5 | -22.9 | 2.9 | 46.2 | 2.3 | -5.9 | 0.1 |

| # | ZINC ID | $\Delta G_{bind}$ | *std* | $\Delta E_{vdW}$ | *std* | $\Delta E_{el}$ | *std* | $\Delta G_{pol}$ | *std* | $\Delta G_{np}$ | *std* |
|---|---|---|---|---|---|---|---|---|---|---|---|
| 39 | ZINC000257299555 | -31.5 | 3.6 | -46.7 | 3.1 | -38.1 | 4.3 | 58.9 | 3.7 | -5.6 | 0.1 |
| 40 | ZINC000075530913 | -31.5 | 3.4 | -49.2 | 2.3 | -20.1 | 2.3 | 43.9 | 2.6 | -6.1 | 0.1 |
| **41** | **ZINC000244816835** | **-31.5** | **3.3** | **-43.8** | **3.0** | **-34.4** | **3.6** | **52.7** | **2.9** | **-6.0** | **0.2** |
| 42 | ZINC000057731402 | -31.4 | 3.6 | -51.2 | 2.6 | -25.6 | 3.7 | 51.4 | 3.0 | -6.1 | 0.1 |
| 43 | ZINC000012888207 | -31.4 | 2.6 | -40.8 | 2.2 | -27.7 | 3.4 | 42.3 | 2.8 | -5.2 | 0.1 |
| 44 | ZINC000091486946 | -31.3 | 3.8 | -52.4 | 2.6 | -21.0 | 2.8 | 48.4 | 4.0 | -6.3 | 0.1 |
| 45 | ZINC000229623928 | -31.2 | 4.0 | -50.6 | 2.5 | -33.6 | 3.7 | 59.2 | 3.9 | -6.1 | 0.1 |
| **46** | **ZINC000025032259** | **-31.0** | **4.1** | **-54.4** | **2.7** | **-22.2** | **3.2** | **52.2** | **4.5** | **-6.7** | **0.1** |
| 47 | ZINC000306141880 | -31.0 | 3.2 | -46.5 | 2.8 | -29.4 | 4.2 | 50.8 | 2.9 | -6.0 | 0.1 |
| 48 | ZINC000021835963 | -31.0 | 3.2 | -48.7 | 2.2 | -25.8 | 3.3 | 49.5 | 2.8 | -6.0 | 0.1 |
| 49 | ZINC000100191495 | -31.0 | 3.6 | -46.6 | 2.6 | -14.5 | 2.6 | 35.6 | 2.8 | -5.4 | 0.1 |
| **50** | **ZINC000002216953** | **-31.0** | **3.3** | **-48.7** | **2.4** | **-24.8** | **3.2** | **48.5** | **3.0** | **-6.0** | **0.1** |
| 51 | ZINC000022241454 | -31.0 | 3.6 | -39.1 | 2.9 | -39.2 | 6.6 | 52.9 | 6.4 | -5.6 | 0.1 |
| 52 | ZINC000100193430 | -30.9 | 2.9 | -47.9 | 2.4 | -14.3 | 2.6 | 36.7 | 2.6 | -5.4 | 0.1 |
| **53** | **ZINC000004998502** | **-30.8** | **4.8** | **-53.5** | **3.3** | **-39.4** | **4.2** | **68.1** | **4.5** | **-6.1** | **0.1** |
| 54 | ZINC000154455008 | -30.6 | 3.7 | -44.9 | 2.8 | -15.4 | 6.2 | 35.8 | 4.2 | -6.0 | 0.1 |
| 55 | ZINC000008316344 | -30.5 | 3.9 | -51.0 | 2.4 | -5.6 | 2.6 | 32.0 | 4.0 | -5.9 | 0.1 |
| 56 | ZINC000095469884 | -30.5 | 3.1 | -40.0 | 2.8 | -35.7 | 3.7 | 50.4 | 2.5 | -5.2 | 0.1 |
| 57 | ZINC000040473994 | -30.5 | 3.1 | -49.6 | 2.8 | -26.5 | 4.0 | 51.3 | 2.7 | -5.8 | 0.1 |
| 58 | ZINC000044874685 | -30.4 | 4.1 | -44.2 | 2.8 | -24.3 | 4.6 | 44.0 | 3.9 | -5.9 | 0.1 |
| **59** | **ZINC000091486133** | **-30.3** | **3.5** | **-52.7** | **2.9** | **-25.7** | **3.2** | **54.4** | **3.2** | **-6.2** | **0.1** |
| 60 | ZINC000194368147 | -30.3 | 3.4 | -48.3 | 2.8 | -31.3 | 3.5 | 54.9 | 2.5 | -5.6 | 0.1 |
| 61 | ZINC000022549345 | -30.2 | 3.5 | -53.9 | 2.1 | -16.6 | 3.8 | 46.7 | 5.3 | -6.5 | 0.1 |
| 62 | ZINC000096238948 | -30.1 | 4.0 | -53.8 | 2.7 | -14.2 | 5.3 | 44.2 | 6.2 | -6.3 | 0.1 |
| 63 | ZINC000091768990 | -30.1 | 3.7 | -52.4 | 2.8 | -14.9 | 2.6 | 43.1 | 2.6 | -5.8 | 0.1 |
| 64 | ZINC000069846908 | -29.9 | 5.0 | -50.0 | 3.0 | -33.3 | 7.7 | 59.4 | 4.2 | -6.0 | 0.1 |
| 65 | ZINC000011781217 | -29.8 | 3.6 | -52.0 | 2.2 | -13.7 | 2.4 | 42.4 | 3.6 | -6.5 | 0.1 |
| 66 | ZINC000257206611 | -29.8 | 5.2 | -55.3 | 3.1 | -11.5 | 5.7 | 43.2 | 7.0 | -6.2 | 0.1 |
| 67 | ZINC000097201269 | -29.7 | 5.8 | -49.4 | 2.6 | -17.3 | 4.2 | 43.5 | 5.6 | -6.4 | 0.1 |
| 68 | ZINC000230113696 | -29.6 | 3.9 | -52.9 | 2.8 | -23.6 | 4.3 | 52.9 | 4.2 | -6.1 | 0.1 |
| 69 | ZINC000012142783 | -29.6 | 5.4 | -51.0 | 2.5 | -22.0 | 5.0 | 49.8 | 5.8 | -6.4 | 0.1 |
| 70 | ZINC000575618196 | -29.6 | 3.9 | -54.5 | 2.7 | -27.3 | 3.9 | 58.5 | 3.6 | -6.2 | 0.1 |
| 71 | ZINC000257222272 | -29.5 | 3.1 | -51.4 | 2.2 | -7.4 | 2.0 | 35.6 | 2.8 | -6.3 | 0.1 |
| **72** | **ZINC000044919578** | **-29.5** | **2.9** | **-43.0** | **2.1** | **-29.3** | **3.4** | **48.4** | **3.1** | **-5.6** | **0.1** |
| 73 | ZINC000257211885 | -29.4 | 5.5 | -50.3 | 3.9 | -20.9 | 3.8 | 47.7 | 4.0 | -5.9 | 0.1 |
| 74 | ZINC000014791001 | -29.4 | 3.4 | -47.0 | 2.3 | -21.6 | 3.5 | 44.7 | 3.0 | -5.5 | 0.1 |
| **75** | **ZINC000008913866** | **-29.3** | **4.4** | **-50.7** | **2.5** | **-29.7** | **6.1** | **56.9** | **5.2** | **-5.9** | **0.1** |
| 76 | ZINC000034895992 | -29.2 | 5.0 | -49.7 | 2.7 | -29.8 | 3.4 | 56.7 | 4.3 | -6.4 | 0.1 |
| 77 | ZINC000171342968 | -29.2 | 4.9 | -48.4 | 2.7 | -40.1 | 3.9 | 65.5 | 3.9 | -6.2 | 0.2 |
| **78** | **ZINC000008595944** | **-29.1** | **4.0** | **-53.1** | **2.3** | **-13.8** | **4.1** | **43.5** | **4.1** | **-5.9** | **0.1** |
| 79 | ZINC000091943530 | -29.0 | 3.9 | -44.1 | 2.7 | -17.5 | 4.4 | 38.7 | 3.3 | -6.0 | 0.1 |
| 80 | ZINC000071746202 | -29.0 | 3.7 | -47.6 | 2.6 | -14.6 | 3.6 | 39.6 | 6.1 | -6.3 | 0.1 |
| 81 | ZINC000257310083 | -28.9 | 3.3 | -47.2 | 2.6 | -17.0 | 2.4 | 41.0 | 2.4 | -5.8 | 0.1 |
| 82 | ZINC000328587242 | -28.9 | 3.2 | -45.2 | 2.7 | -24.8 | 2.6 | 47.4 | 2.6 | -6.4 | 0.1 |
| 83 | ZINC000012323932 | -28.9 | 5.3 | -42.7 | 3.2 | -38.4 | 7.2 | 57.7 | 3.8 | -5.5 | 0.2 |
| 84 | ZINC000075118384 | -28.8 | 4.8 | -46.9 | 2.9 | -33.1 | 7.3 | 57.3 | 4.9 | -6.1 | 0.1 |

| # | ZINC ID | Δ$G_{bind}$ | *std* | Δ$E_{vdW}$ | *std* | Δ$E_{el}$ | *std* | Δ$G_{pol}$ | *std* | Δ$G_{np}$ | *std* |
|---|---|---|---|---|---|---|---|---|---|---|---|
| 85 | ZINC000585280336 | -28.8 | 5.1 | -51.7 | 2.3 | -29.6 | 5.8 | 58.2 | 4.5 | -5.7 | 0.1 |
| 86 | ZINC000096251711 | -28.8 | 3.6 | -50.9 | 3.0 | -15.0 | 3.5 | 43.0 | 3.3 | -5.9 | 0.1 |
| 87 | ZINC000097413355 | -28.8 | 2.9 | -46.7 | 2.6 | -27.2 | 3.2 | 50.9 | 3.4 | -5.7 | 0.1 |
| 88 | ZINC000257192562 | -28.7 | 3.1 | -45.9 | 2.2 | -17.6 | 2.3 | 40.7 | 2.5 | -5.9 | 0.1 |
| 89 | ZINC000095951555 | -28.7 | 2.9 | -42.9 | 2.3 | -19.4 | 3.3 | 38.9 | 2.7 | -5.4 | 0.1 |
| 90 | ZINC000007030648 | -28.7 | 5.4 | -43.3 | 2.9 | -21.2 | 7.0 | 41.2 | 3.4 | -5.3 | 0.1 |
| 91 | ZINC000096003964 | -28.7 | 4.9 | -49.1 | 2.6 | -26.9 | 4.8 | 52.8 | 3.3 | -5.5 | 0.1 |
| **92** | **ZINC000004511530** | **-28.5** | **3.3** | **-49.2** | **2.1** | **-22.4** | **3.0** | **48.9** | **3.0** | **-5.7** | **0.1** |
| 93 | ZINC000409428381 | -28.4 | 3.8 | -51.5 | 2.4 | -12.1 | 4.9 | 41.9 | 4.8 | -6.6 | 0.1 |
| 94 | ZINC000096158052 | -28.4 | 3.6 | -49.9 | 2.5 | -20.8 | 3.8 | 48.9 | 4.1 | -6.6 | 0.1 |
| 95 | ZINC000057526426 | -28.3 | 3.0 | -44.4 | 2.2 | -28.2 | 3.1 | 50.3 | 3.0 | -5.9 | 0.1 |
| 96 | ZINC000014741626 | -28.3 | 3.7 | -49.6 | 2.2 | -7.1 | 2.5 | 34.8 | 4.5 | -6.3 | 0.1 |
| 97 | ZINC000072290626 | -28.2 | 4.5 | -48.7 | 2.2 | -18.8 | 5.2 | 45.2 | 5.8 | -6.0 | 0.1 |
| 98 | ZINC000067643674 | -28.2 | 3.4 | -44.8 | 2.5 | -25.4 | 4.2 | 47.5 | 3.2 | -5.6 | 0.1 |
| 99 | ZINC000077503523 | -28.2 | 3.7 | -45.1 | 2.9 | -34.1 | 6.6 | 56.3 | 4.6 | -5.4 | 0.1 |
| 100 | ZINC000100737122 | -28.1 | 3.3 | -52.3 | 2.5 | -22.6 | 3.1 | 52.8 | 4.1 | -6.0 | 0.1 |
| 101 | ZINC000013973188 | -28.1 | 4.1 | -47.2 | 2.5 | -29.0 | 3.6 | 53.8 | 3.7 | -5.7 | 0.1 |
| 102 | ZINC000069775761 | -28.1 | 4.2 | -48.1 | 2.6 | -21.4 | 4.1 | 47.3 | 3.1 | -6.0 | 0.2 |
| 103 | ZINC000225489282 | -28.0 | 3.7 | -49.8 | 2.4 | -17.0 | 4.8 | 44.6 | 4.4 | -5.8 | 0.1 |
| 104 | ZINC000008738218 | -27.9 | 3.8 | -41.0 | 2.9 | -41.3 | 5.6 | 59.7 | 3.9 | -5.3 | 0.1 |
| 105 | ZINC000016228943 | -27.9 | 3.1 | -43.6 | 2.6 | -26.4 | 3.3 | 47.8 | 3.1 | -5.7 | 0.1 |
| 106 | ZINC000035671101 | -27.9 | 3.4 | -46.7 | 2.0 | -8.1 | 1.6 | 32.5 | 3.3 | -5.7 | 0.1 |
| 107 | ZINC000009338249 | -27.6 | 4.9 | -47.0 | 2.5 | -27.3 | 4.7 | 52.1 | 4.0 | -5.4 | 0.1 |
| 108 | ZINC000069492530 | -27.6 | 4.4 | -47.5 | 2.6 | -19.0 | 3.3 | 44.4 | 3.1 | -5.6 | 0.1 |
| 109 | ZINC000119429896 | -27.6 | 3.6 | -49.5 | 2.2 | -11.7 | 2.9 | 39.9 | 3.3 | -6.3 | 0.1 |
| 110 | ZINC000585093122 | -27.5 | 3.5 | -45.2 | 2.6 | -16.9 | 4.4 | 40.4 | 4.0 | -5.8 | 0.1 |
| 111 | ZINC000065248686 | -27.5 | 3.3 | -45.7 | 2.6 | -14.8 | 2.7 | 38.6 | 2.3 | -5.7 | 0.1 |
| 112 | ZINC000003438521 | -27.5 | 3.4 | -45.4 | 2.3 | -24.1 | 4.5 | 47.7 | 4.2 | -5.7 | 0.1 |
| 113 | ZINC000095393415 | -27.4 | 4.4 | -54.8 | 2.5 | -14.4 | 3.2 | 47.4 | 5.0 | -5.7 | 0.1 |
| 114 | ZINC000079001413 | -27.4 | 3.4 | -52.9 | 2.5 | -9.0 | 2.5 | 40.5 | 2.8 | -6.0 | 0.1 |
| 115 | ZINC000238121810 | -27.4 | 5.9 | -37.2 | 2.9 | -110.9 | 10.4 | 126.7 | 8.2 | -6.0 | 0.1 |
| 116 | ZINC000007030710 | -27.4 | 3.9 | -47.3 | 2.7 | -25.7 | 4.3 | 51.3 | 3.2 | -5.6 | 0.1 |
| 117 | ZINC000002750765 | -27.3 | 3.6 | -45.0 | 2.4 | -24.2 | 2.9 | 47.7 | 3.7 | -5.9 | 0.1 |
| 118 | ZINC000100119126 | -27.3 | 4.2 | -47.3 | 2.8 | -19.9 | 4.3 | 45.8 | 4.0 | -5.8 | 0.1 |
| **119** | **ZINC000069778215** | **-27.2** | **3.7** | **-47.6** | **2.7** | **-41.7** | **4.2** | **68.1** | **3.8** | **-6.1** | **0.1** |
| 120 | ZINC000092021781 | -27.2 | 3.8 | -49.3 | 2.9 | -21.9 | 5.0 | 50.3 | 3.8 | -6.2 | 0.1 |
| 121 | ZINC000035398834 | -27.2 | 3.3 | -51.5 | 2.3 | -8.9 | 2.5 | 38.9 | 3.3 | -5.7 | 0.1 |
| 122 | ZINC000013691850 | -27.2 | 2.9 | -49.9 | 2.5 | -15.3 | 3.3 | 43.5 | 2.5 | -5.4 | 0.1 |
| 123 | ZINC000050859891 | -27.2 | 4.1 | -48.4 | 2.6 | -28.7 | 4.7 | 55.6 | 5.3 | -5.7 | 0.1 |
| 124 | ZINC000003292603 | -27.1 | 3.3 | -47.2 | 2.4 | -19.2 | 3.7 | 45.4 | 4.2 | -6.1 | 0.1 |
| 125 | ZINC000058099587 | -27.1 | 4.3 | -46.8 | 2.8 | -18.8 | 3.4 | 44.6 | 4.2 | -6.0 | 0.1 |
| 126 | ZINC000050584912 | -27.1 | 4.2 | -50.3 | 2.8 | -6.7 | 3.9 | 36.0 | 5.2 | -6.2 | 0.1 |
| 127 | ZINC000071754931 | -26.9 | 3.2 | -40.8 | 2.3 | -12.7 | 3.3 | 32.0 | 3.0 | -5.5 | 0.1 |
| 128 | ZINC000075140034 | -26.8 | 3.1 | -41.8 | 2.4 | -19.7 | 2.9 | 40.8 | 3.1 | -6.1 | 0.1 |
| 129 | ZINC000069349055 | -26.8 | 5.7 | -48.3 | 3.0 | -24.4 | 8.4 | 52.3 | 6.0 | -6.4 | 0.1 |
| 130 | ZINC000064871452 | -26.8 | 4.5 | -49.9 | 2.5 | -26.3 | 7.2 | 55.5 | 6.7 | -6.1 | 0.1 |

| # | ZINC ID | $\Delta G_{bind}$ | *std* | $\Delta E_{vdW}$ | *std* | $\Delta E_{el}$ | *std* | $\Delta G_{pol}$ | *std* | $\Delta G_{np}$ | *std* |
|---|---|---|---|---|---|---|---|---|---|---|---|
| 131 | ZINC000238886413 | -26.8 | 4.6 | -49.4 | 2.7 | -11.3 | 3.3 | 39.7 | 3.1 | -5.7 | 0.1 |
| 132 | ZINC000040548686 | -26.7 | 3.9 | -51.5 | 2.2 | -22.0 | 4.2 | 52.6 | 3.9 | -5.8 | 0.1 |
| 133 | ZINC000019973951 | -26.7 | 4.1 | -40.3 | 3.0 | -29.7 | 6.5 | 49.1 | 5.4 | -5.7 | 0.1 |
| 134 | ZINC000002646979 | -26.6 | 3.8 | -51.5 | 2.3 | -22.4 | 3.2 | 52.9 | 3.5 | -5.7 | 0.1 |
| 135 | ZINC000257218603 | -26.6 | 4.1 | -50.2 | 2.5 | -24.7 | 5.1 | 54.9 | 4.9 | -6.6 | 0.1 |
| 136 | ZINC000044908085 | -26.4 | 4.2 | -51.1 | 2.7 | -21.0 | 3.4 | 51.5 | 3.5 | -5.9 | 0.1 |
| **137** | **ZINC000023642159** | **-26.4** | **3.8** | **-47.6** | **2.3** | **-27.5** | **4.0** | **54.8** | **4.0** | **-6.2** | **0.1** |
| 138 | ZINC000092760184 | -26.4 | 3.3 | -47.5 | 2.4 | -20.6 | 3.3 | 47.9 | 3.0 | -6.3 | 0.1 |
| 139 | ZINC000257256038 | -26.3 | 3.6 | -45.9 | 2.4 | -25.3 | 3.1 | 50.7 | 3.0 | -5.9 | 0.1 |
| 140 | ZINC000074014922 | -26.2 | 2.7 | -47.0 | 2.1 | -10.1 | 2.3 | 36.3 | 2.1 | -5.5 | 0.1 |
| 141 | ZINC000072234903 | -26.2 | 3.2 | -44.1 | 2.2 | -12.2 | 3.9 | 35.8 | 5.0 | -5.6 | 0.1 |
| 142 | ZINC000050857093 | -26.2 | 5.0 | -48.7 | 2.5 | -24.6 | 3.8 | 53.0 | 4.2 | -5.9 | 0.1 |
| 143 | ZINC000018240833 | -26.2 | 4.3 | -47.5 | 2.8 | -36.3 | 5.3 | 63.8 | 4.2 | -6.2 | 0.1 |
| 144 | ZINC000095447759 | -26.1 | 4.4 | -46.9 | 2.9 | -24.7 | 5.4 | 51.0 | 3.8 | -5.5 | 0.1 |
| 145 | ZINC000016543876 | -26.1 | 4.1 | -46.0 | 2.3 | -18.4 | 3.8 | 44.0 | 2.6 | -5.7 | 0.1 |
| 146 | ZINC000010218408 | -26.0 | 5.8 | -47.3 | 2.8 | -19.0 | 5.6 | 45.8 | 5.0 | -5.5 | 0.1 |
| 147 | ZINC000018053795 | -25.9 | 4.5 | -47.5 | 2.3 | -23.2 | 3.7 | 50.7 | 4.3 | -5.9 | 0.1 |
| 148 | ZINC000072896859 | -25.9 | 4.2 | -48.3 | 2.6 | -6.4 | 5.0 | 34.9 | 5.7 | -6.1 | 0.1 |
| 149 | ZINC000042025678 | -25.9 | 3.6 | -43.6 | 4.1 | -13.2 | 5.4 | 36.9 | 3.6 | -5.9 | 0.1 |
| 150 | ZINC000040060202 | -25.8 | 3.3 | -46.1 | 2.8 | -17.7 | 3.4 | 44.3 | 3.3 | -6.3 | 0.1 |
| 151 | ZINC000067460397 | -25.7 | 3.3 | -50.1 | 2.2 | -34.7 | 3.6 | 64.7 | 3.6 | -5.6 | 0.1 |
| 152 | ZINC000584887110 | -25.7 | 3.5 | -46.9 | 2.2 | -13.7 | 2.9 | 40.8 | 3.5 | -5.9 | 0.1 |
| 153 | ZINC000096837115 | -25.6 | 5.0 | -45.6 | 4.0 | -26.8 | 7.8 | 53.1 | 8.2 | -6.3 | 0.1 |
| 154 | ZINC000071282106 | -25.6 | 4.6 | -47.7 | 2.6 | -16.3 | 4.9 | 44.5 | 4.5 | -6.1 | 0.1 |
| 155 | ZINC000091304527 | -25.6 | 3.8 | -50.9 | 2.5 | -14.3 | 4.5 | 45.8 | 5.0 | -6.2 | 0.1 |
| 156 | ZINC000003875380 | -25.6 | 3.1 | -46.6 | 1.9 | -16.5 | 2.0 | 43.0 | 3.2 | -5.5 | 0.1 |
| 157 | ZINC000003313076 | -25.5 | 3.5 | -41.7 | 3.0 | -21.4 | 4.5 | 42.7 | 2.9 | -5.2 | 0.1 |
| 158 | ZINC000052955795 | -25.5 | 4.2 | -46.7 | 2.6 | -27.4 | 4.9 | 54.8 | 3.2 | -6.1 | 0.1 |
| 159 | ZINC000170800517 | -25.5 | 3.2 | -49.4 | 2.4 | -18.6 | 2.4 | 48.8 | 3.0 | -6.2 | 0.1 |
| 160 | ZINC000178511392 | -25.4 | 3.8 | -40.2 | 2.5 | -33.4 | 4.1 | 53.6 | 3.1 | -5.4 | 0.1 |
| 161 | ZINC000069595091 | -25.3 | 4.1 | -45.8 | 2.8 | -22.3 | 3.9 | 49.0 | 6.2 | -6.2 | 0.2 |
| 162 | ZINC000096251709 | -25.2 | 3.5 | -48.0 | 2.1 | -19.0 | 3.2 | 47.7 | 3.0 | -5.9 | 0.1 |
| 163 | ZINC000028790813 | -25.2 | 5.4 | -51.7 | 2.7 | -23.6 | 5.6 | 56.4 | 5.4 | -6.3 | 0.1 |
| 164 | ZINC000153353600 | -25.1 | 4.4 | -46.2 | 2.7 | -9.4 | 3.9 | 36.8 | 4.6 | -6.2 | 0.2 |
| 165 | ZINC000072096437 | -25.1 | 4.0 | -50.9 | 2.4 | -18.7 | 4.2 | 50.8 | 4.1 | -6.3 | 0.1 |
| 166 | ZINC000002922856 | -25.1 | 3.8 | -52.4 | 2.7 | -15.2 | 3.6 | 48.4 | 4.4 | -5.9 | 0.1 |
| 167 | ZINC000077353582 | -25.1 | 4.3 | -48.6 | 2.8 | -15.7 | 3.0 | 44.8 | 2.7 | -5.5 | 0.1 |
| 168 | ZINC000072300733 | -25.0 | 5.1 | -51.3 | 2.7 | -13.5 | 4.4 | 45.6 | 3.4 | -5.9 | 0.1 |
| 169 | ZINC000000326909 | -25.0 | 3.5 | -42.9 | 2.1 | -16.5 | 3.4 | 39.7 | 2.6 | -5.2 | 0.1 |
| 170 | ZINC000002630701 | -24.9 | 3.6 | -49.0 | 2.0 | -7.6 | 4.1 | 37.4 | 4.3 | -5.7 | 0.1 |
| 171 | ZINC000084563579 | -24.8 | 4.1 | -45.4 | 4.0 | -21.3 | 15.9 | 48.1 | 12.0 | -6.3 | 0.1 |
| 172 | ZINC000177842371 | -24.8 | 3.4 | -45.3 | 2.0 | -26.9 | 3.7 | 52.7 | 4.0 | -5.3 | 0.1 |
| 173 | ZINC000306138347 | -24.8 | 3.3 | -49.7 | 2.0 | -17.4 | 3.2 | 48.0 | 3.1 | -5.8 | 0.1 |
| 174 | ZINC000095503625 | -24.8 | 4.1 | -50.6 | 2.6 | -24.3 | 4.9 | 56.3 | 5.0 | -6.2 | 0.1 |
| 175 | ZINC000023764785 | -24.7 | 5.5 | -45.9 | 2.6 | -14.8 | 4.5 | 42.0 | 4.4 | -6.1 | 0.1 |
| 176 | ZINC000005426253 | -24.7 | 3.8 | -49.8 | 2.4 | -12.6 | 3.2 | 44.1 | 4.2 | -6.3 | 0.1 |

| # | ZINC ID | $\Delta G_{bind}$ | *std* | $\Delta E_{vdW}$ | *std* | $\Delta E_{el}$ | *std* | $\Delta G_{pol}$ | *std* | $\Delta G_{np}$ | *std* |
|---|---|---|---|---|---|---|---|---|---|---|---|
| 177 | ZINC000072290753 | -24.6 | 4.2 | -52.3 | 2.4 | -25.4 | 4.1 | 58.9 | 3.8 | -5.8 | 0.1 |
| 178 | ZINC000095402766 | -24.6 | 5.5 | -44.9 | 2.5 | -17.6 | 7.1 | 43.9 | 4.0 | -6.0 | 0.1 |
| 179 | ZINC000072290711 | -24.6 | 3.7 | -46.7 | 2.9 | -17.3 | 3.3 | 44.9 | 4.1 | -5.5 | 0.1 |
| 180 | ZINC000069740630 | -24.6 | 5.3 | -38.0 | 2.6 | -20.8 | 4.5 | 40.0 | 3.2 | -5.8 | 0.1 |
| 181 | ZINC000019327176 | -24.5 | 4.7 | -40.2 | 3.0 | -26.9 | 5.7 | 48.2 | 6.1 | -5.7 | 0.1 |
| 182 | ZINC000008776545 | -24.5 | 3.4 | -47.3 | 2.4 | -15.6 | 2.9 | 44.0 | 2.5 | -5.6 | 0.1 |
| 183 | ZINC000003641308 | -24.5 | 4.5 | -45.0 | 2.6 | -25.1 | 7.2 | 51.3 | 8.7 | -5.6 | 0.2 |
| 184 | ZINC000096024249 | -24.5 | 4.2 | -47.4 | 2.4 | -17.8 | 5.0 | 45.9 | 4.0 | -5.2 | 0.1 |
| 185 | ZINC000008771448 | -24.4 | 4.8 | -48.5 | 3.0 | -22.3 | 5.8 | 52.5 | 6.8 | -6.1 | 0.2 |
| 186 | ZINC000065167646 | -24.4 | 5.6 | -47.8 | 2.7 | -22.4 | 4.0 | 51.6 | 6.2 | -5.8 | 0.1 |
| 187 | ZINC000005026046 | -24.4 | 3.6 | -45.8 | 2.3 | -14.0 | 2.7 | 41.3 | 4.9 | -5.9 | 0.1 |
| 188 | ZINC000072235028 | -24.2 | 3.6 | -52.8 | 2.4 | -12.0 | 4.3 | 46.7 | 4.0 | -6.1 | 0.1 |
| 189 | ZINC000072277544 | -24.2 | 3.9 | -45.7 | 2.8 | -14.8 | 5.4 | 42.2 | 5.1 | -5.8 | 0.1 |
| 190 | ZINC000192753414 | -24.2 | 4.5 | -46.9 | 2.7 | -33.1 | 4.6 | 61.1 | 3.1 | -5.4 | 0.1 |
| 191 | ZINC000245171655 | -24.2 | 3.1 | -48.7 | 2.3 | -10.2 | 2.7 | 40.7 | 2.6 | -6.0 | 0.1 |
| 192 | ZINC000065463577 | -24.1 | 3.9 | -46.6 | 2.7 | -31.9 | 5.2 | 60.0 | 4.3 | -5.5 | 0.1 |
| 193 | ZINC000135810445 | -24.1 | 4.5 | -44.5 | 2.6 | -33.4 | 5.4 | 59.2 | 5.0 | -5.5 | 0.1 |
| 194 | ZINC000096010936 | -24.1 | 3.0 | -41.4 | 2.8 | -27.6 | 3.2 | 50.7 | 2.5 | -5.8 | 0.1 |
| 195 | ZINC000071497775 | -24.0 | 4.2 | -51.3 | 2.3 | -25.4 | 6.6 | 58.9 | 5.6 | -6.2 | 0.1 |
| 196 | ZINC000040386093 | -24.0 | 3.7 | -45.0 | 2.7 | -14.5 | 3.0 | 41.3 | 3.1 | -5.7 | 0.1 |
| 197 | ZINC000096122792 | -24.0 | 3.4 | -48.6 | 2.6 | -9.0 | 2.7 | 39.2 | 3.4 | -5.6 | 0.1 |
| 198 | ZINC000278659999 | -24.0 | 4.3 | -47.8 | 2.4 | -23.5 | 3.5 | 53.4 | 4.5 | -6.1 | 0.1 |
| 199 | ZINC000029134571 | -24.0 | 3.3 | -52.1 | 2.4 | -19.8 | 3.2 | 53.9 | 3.3 | -5.9 | 0.1 |
| 200 | ZINC000096038482 | -23.9 | 4.1 | -39.5 | 2.9 | -25.3 | 6.7 | 46.8 | 5.7 | -5.9 | 0.1 |
| 201 | ZINC000253433259 | -23.9 | 3.2 | -49.1 | 2.2 | -19.7 | 2.8 | 50.4 | 2.6 | -5.5 | 0.1 |
| 202 | ZINC000097105546 | -23.9 | 4.0 | -44.7 | 2.6 | -21.9 | 3.7 | 48.0 | 3.2 | -5.4 | 0.1 |
| 203 | ZINC000062639858 | -23.9 | 4.0 | -52.0 | 2.2 | -3.7 | 3.5 | 38.2 | 4.7 | -6.5 | 0.1 |
| 204 | ZINC000013691849 | -23.8 | 4.6 | -44.2 | 3.0 | -33.9 | 4.8 | 59.9 | 4.1 | -5.5 | 0.1 |
| 205 | ZINC000001381807 | -23.7 | 2.7 | -37.1 | 2.1 | -15.1 | 2.3 | 33.5 | 2.9 | -5.1 | 0.1 |
| 206 | ZINC000006669352 | -23.7 | 3.8 | -47.6 | 2.2 | -23.6 | 5.5 | 53.3 | 5.4 | -5.8 | 0.1 |
| 207 | ZINC000091664087 | -23.3 | 4.4 | -54.4 | 2.3 | -8.4 | 3.4 | 45.5 | 3.8 | -6.1 | 0.1 |
| 208 | ZINC000014240188 | -23.3 | 3.6 | -45.7 | 2.8 | -19.3 | 4.2 | 47.2 | 4.0 | -5.6 | 0.1 |
| 209 | ZINC000012550491 | -23.3 | 3.2 | -46.4 | 2.6 | -24.3 | 3.5 | 53.3 | 2.9 | -5.8 | 0.1 |
| 210 | ZINC000095989182 | -23.2 | 3.1 | -43.2 | 2.2 | -19.0 | 3.6 | 44.1 | 2.9 | -5.2 | 0.1 |
| 211 | ZINC000257311417 | -23.2 | 3.3 | -40.7 | 2.2 | -22.2 | 4.6 | 45.6 | 4.8 | -5.9 | 0.2 |
| 212 | ZINC000013692677 | -23.1 | 4.8 | -51.9 | 3.1 | -30.2 | 4.7 | 65.1 | 5.2 | -6.2 | 0.1 |
| 213 | ZINC000004535480 | -23.1 | 4.3 | -45.6 | 2.2 | -15.5 | 4.7 | 43.2 | 5.4 | -5.2 | 0.1 |
| 214 | ZINC000002465084 | -23.1 | 4.0 | -49.8 | 2.5 | -23.0 | 3.3 | 55.5 | 4.5 | -5.8 | 0.1 |
| 215 | ZINC000003327891 | -23.1 | 3.1 | -45.9 | 2.2 | -11.5 | 2.2 | 40.1 | 3.2 | -5.8 | 0.1 |
| 216 | ZINC000006782318 | -23.1 | 3.5 | -47.7 | 2.3 | -17.2 | 2.7 | 47.6 | 3.9 | -5.8 | 0.1 |
| 217 | ZINC000040386098 | -23.0 | 3.6 | -42.7 | 2.5 | -14.7 | 3.4 | 39.9 | 3.3 | -5.6 | 0.1 |
| 218 | ZINC000085879141 | -23.0 | 3.3 | -41.7 | 2.4 | -10.0 | 5.3 | 34.6 | 5.2 | -5.9 | 0.1 |
| 219 | ZINC000006117573 | -22.9 | 3.4 | -42.2 | 2.4 | -29.4 | 2.9 | 54.6 | 3.4 | -5.8 | 0.1 |
| 220 | ZINC000005088834 | -22.7 | 3.5 | -42.9 | 3.6 | -8.5 | 2.5 | 34.6 | 3.1 | -5.9 | 0.1 |
| 221 | ZINC000097050589 | -22.7 | 4.8 | -48.8 | 2.6 | -13.0 | 4.7 | 45.0 | 5.3 | -5.8 | 0.1 |
| 222 | ZINC000065395709 | -22.6 | 4.0 | -51.6 | 2.4 | -22.5 | 3.1 | 57.5 | 3.9 | -6.0 | 0.1 |

| # | ZINC ID | $\Delta G_{bind}$ | *std* | $\Delta E_{vdW}$ | *std* | $\Delta E_{el}$ | *std* | $\Delta G_{pol}$ | *std* | $\Delta G_{np}$ | *std* |
|---|---|---|---|---|---|---|---|---|---|---|---|
| 223 | ZINC000004495846 | -22.6 | 5.1 | -46.3 | 2.7 | -25.8 | 4.2 | 55.3 | 4.7 | -5.8 | 0.1 |
| 224 | ZINC000072437791 | -22.5 | 3.2 | -46.5 | 2.2 | -14.0 | 3.0 | 43.6 | 3.0 | -5.6 | 0.1 |
| 225 | ZINC000027755553 | -22.5 | 3.4 | -46.7 | 2.1 | -15.9 | 3.0 | 46.1 | 3.3 | -6.0 | 0.1 |
| 226 | ZINC000096069207 | -22.5 | 3.7 | -52.2 | 2.8 | -19.5 | 2.9 | 55.3 | 3.9 | -6.1 | 0.1 |
| 227 | ZINC000057771728 | -22.4 | 4.4 | -48.2 | 2.5 | -23.3 | 5.5 | 55.2 | 5.7 | -6.1 | 0.1 |
| 228 | ZINC000239762559 | -22.4 | 4.5 | -48.8 | 2.5 | -20.7 | 4.1 | 53.2 | 5.1 | -6.0 | 0.1 |
| 229 | ZINC000003852421 | -22.3 | 3.2 | -44.3 | 2.1 | -12.6 | 2.6 | 39.9 | 2.8 | -5.3 | 0.1 |
| 230 | ZINC000091321614 | -22.2 | 4.4 | -49.4 | 2.4 | -18.1 | 3.8 | 51.6 | 5.5 | -6.2 | 0.1 |
| 231 | ZINC000299793701 | -22.0 | 4.2 | -42.4 | 2.8 | -25.9 | 5.0 | 51.8 | 3.9 | -5.6 | 0.1 |
| 232 | ZINC000012407997 | -22.0 | 4.4 | -48.0 | 2.8 | -11.5 | 5.7 | 43.9 | 7.2 | -6.3 | 0.1 |
| 233 | ZINC000584897275 | -21.8 | 5.0 | -44.7 | 2.8 | -36.2 | 4.3 | 65.5 | 6.0 | -6.5 | 0.1 |
| 234 | ZINC000096069697 | -21.8 | 5.2 | -45.5 | 2.3 | -9.5 | 5.4 | 39.1 | 4.3 | -5.8 | 0.1 |
| 235 | ZINC000257257831 | -21.8 | 3.7 | -45.8 | 2.2 | -13.1 | 4.8 | 42.6 | 4.5 | -5.5 | 0.1 |
| 236 | ZINC000253407066 | -21.7 | 3.6 | -46.7 | 2.4 | -13.2 | 3.4 | 43.9 | 3.3 | -5.8 | 0.1 |
| 237 | ZINC000001810957 | -21.6 | 4.4 | -45.8 | 2.6 | -17.6 | 3.6 | 47.5 | 4.6 | -5.7 | 0.1 |
| 238 | ZINC000000489138 | -21.6 | 3.7 | -50.3 | 2.3 | -20.1 | 2.9 | 54.4 | 3.7 | -5.7 | 0.1 |
| 239 | ZINC000330033487 | -21.5 | 4.2 | -50.1 | 2.6 | -15.4 | 4.4 | 50.2 | 3.6 | -6.2 | 0.1 |
| 240 | ZINC000057511709 | -21.5 | 4.8 | -45.5 | 2.0 | -21.3 | 5.0 | 51.2 | 7.8 | -5.9 | 0.1 |
| 241 | ZINC000006887927 | -21.1 | 5.0 | -48.4 | 2.6 | -23.5 | 3.7 | 57.1 | 5.3 | -6.3 | 0.1 |
| 242 | ZINC000096080454 | -21.1 | 3.1 | -44.4 | 2.3 | -19.0 | 2.8 | 47.9 | 3.0 | -5.6 | 0.1 |
| 243 | ZINC000040385901 | -21.0 | 3.8 | -41.1 | 2.8 | -13.7 | 2.9 | 39.3 | 3.2 | -5.4 | 0.1 |
| 244 | ZINC000237955843 | -20.9 | 4.2 | -40.6 | 2.6 | -64.6 | 10.7 | 90.5 | 10.6 | -6.3 | 0.2 |
| 245 | ZINC000091487017 | -20.8 | 4.0 | -49.3 | 2.3 | -11.6 | 3.0 | 46.2 | 3.6 | -6.2 | 0.1 |
| 246 | ZINC000004722011 | -20.8 | 3.3 | -44.0 | 2.2 | -7.3 | 2.7 | 36.4 | 3.1 | -5.9 | 0.1 |
| 247 | ZINC000257270100 | -20.8 | 5.7 | -46.1 | 2.5 | -11.5 | 2.9 | 42.6 | 4.7 | -5.8 | 0.1 |
| 248 | ZINC000009318781 | -20.5 | 4.5 | -46.3 | 2.8 | -25.5 | 6.6 | 56.7 | 4.9 | -5.5 | 0.1 |
| 249 | ZINC000575617533 | -20.5 | 3.9 | -44.8 | 2.3 | -10.6 | 3.0 | 41.3 | 4.7 | -6.4 | 0.2 |
| 250 | ZINC000065591272 | -20.5 | 3.6 | -44.9 | 2.2 | -10.8 | 2.9 | 41.1 | 3.3 | -5.9 | 0.1 |
| 251 | ZINC000005303591 | -20.5 | 3.9 | -44.2 | 2.5 | -20.1 | 3.9 | 49.7 | 4.2 | -5.9 | 0.1 |
| 252 | ZINC000077522487 | -20.5 | 4.1 | -48.6 | 2.2 | -27.2 | 5.2 | 61.8 | 6.1 | -6.5 | 0.1 |
| 253 | ZINC000072807915 | -20.4 | 4.7 | -50.6 | 2.3 | -8.1 | 3.6 | 44.8 | 5.8 | -6.4 | 0.1 |
| 254 | ZINC000019328346 | -20.2 | 4.4 | -46.7 | 4.6 | -22.0 | 4.6 | 54.4 | 6.0 | -5.9 | 0.1 |
| 255 | ZINC000237740654 | -20.2 | 4.5 | -45.0 | 2.8 | -28.0 | 10.4 | 59.0 | 11.9 | -6.2 | 0.1 |
| 256 | ZINC000048249797 | -20.2 | 4.7 | -50.8 | 2.7 | -10.4 | 5.2 | 47.3 | 4.4 | -6.3 | 0.1 |
| 257 | ZINC000002390471 | -20.1 | 3.3 | -43.9 | 2.0 | -12.0 | 2.3 | 41.8 | 3.1 | -5.9 | 0.1 |
| 258 | ZINC000030875068 | -20.1 | 4.1 | -45.9 | 2.2 | -11.0 | 3.9 | 42.4 | 4.5 | -5.7 | 0.1 |
| 259 | ZINC000107713021 | -19.9 | 4.2 | -44.7 | 2.2 | -3.4 | 2.9 | 34.5 | 3.7 | -6.2 | 0.1 |
| 260 | ZINC000068269627 | -19.9 | 5.5 | -50.4 | 2.4 | -26.9 | 4.4 | 63.7 | 7.4 | -6.2 | 0.1 |
| 261 | ZINC000089857747 | -19.7 | 3.7 | -50.7 | 4.5 | -7.1 | 5.1 | 43.9 | 9.7 | -5.8 | 0.1 |
| 262 | ZINC000000079035 | -19.6 | 5.7 | -47.2 | 2.3 | -20.0 | 5.4 | 53.8 | 4.3 | -6.2 | 0.1 |
| 263 | ZINC000022067509 | -19.6 | 8.1 | -49.1 | 2.8 | -56.9 | 11.1 | 92.3 | 9.7 | -5.9 | 0.1 |
| 264 | ZINC000001620500 | -19.6 | 7.0 | -42.1 | 2.8 | -51.7 | 24.4 | 80.2 | 19.2 | -6.0 | 0.1 |
| 265 | ZINC000005244779 | -19.6 | 4.5 | -49.8 | 2.4 | -9.1 | 2.7 | 45.4 | 4.4 | -6.1 | 0.1 |
| 266 | ZINC000040266307 | -19.6 | 4.2 | -44.5 | 2.5 | -22.5 | 4.2 | 53.2 | 3.9 | -5.8 | 0.1 |
| 267 | ZINC000253473237 | -19.5 | 3.4 | -44.9 | 2.2 | -8.2 | 2.9 | 39.7 | 3.1 | -6.0 | 0.1 |
| 268 | ZINC000017888288 | -19.4 | 4.7 | -44.1 | 2.3 | -16.9 | 4.4 | 47.3 | 4.5 | -5.6 | 0.1 |

| # | ZINC ID | $\Delta G_{bind}$ | *std* | $\Delta E_{vdW}$ | *std* | $\Delta E_{el}$ | *std* | $\Delta G_{pol}$ | *std* | $\Delta G_{np}$ | *std* |
|---|---|---|---|---|---|---|---|---|---|---|---|
| 269 | ZINC000012122382 | -19.2 | 6.0 | -42.6 | 2.3 | -9.5 | 4.8 | 38.9 | 6.5 | -6.1 | 0.2 |
| 270 | ZINC000002837442 | -19.0 | 3.6 | -42.9 | 2.8 | -16.2 | 2.5 | 46.0 | 3.6 | -5.9 | 0.2 |
| 271 | ZINC000012239509 | -19.0 | 5.3 | -50.5 | 2.5 | -8.0 | 5.0 | 45.8 | 3.9 | -6.3 | 0.1 |
| 272 | ZINC000004732939 | -19.0 | 3.5 | -43.8 | 2.2 | -13.6 | 3.0 | 44.6 | 3.9 | -6.1 | 0.1 |
| 273 | ZINC000253433605 | -19.0 | 5.1 | -47.3 | 2.5 | -14.9 | 7.6 | 49.1 | 5.6 | -5.9 | 0.1 |
| 274 | ZINC000006918427 | -18.6 | 4.4 | -45.0 | 2.5 | -13.1 | 4.0 | 45.2 | 4.2 | -5.7 | 0.1 |
| 275 | ZINC000019796838 | -18.4 | 4.6 | -46.3 | 2.1 | -10.9 | 3.4 | 44.4 | 3.9 | -5.7 | 0.1 |
| 276 | ZINC000008609997 | -18.2 | 3.9 | -50.6 | 2.4 | -12.7 | 3.4 | 51.3 | 4.6 | -6.2 | 0.1 |
| 277 | ZINC000005348603 | -18.1 | 4.3 | -46.3 | 2.6 | -9.9 | 4.8 | 43.7 | 5.3 | -5.6 | 0.1 |
| 278 | ZINC000047209496 | -17.9 | 5.5 | -44.4 | 2.4 | -12.9 | 5.2 | 45.4 | 9.0 | -5.9 | 0.1 |
| 279 | ZINC000072432742 | -17.9 | 5.0 | -49.1 | 2.3 | -23.0 | 9.9 | 60.3 | 10.1 | -6.1 | 0.1 |
| 280 | ZINC000192929211 | -17.7 | 6.2 | -44.2 | 2.7 | -50.4 | 8.5 | 82.9 | 7.4 | -5.9 | 0.1 |
| 281 | ZINC000136345745 | -17.6 | 4.7 | -51.8 | 2.4 | -18.5 | 4.8 | 58.7 | 4.6 | -6.0 | 0.1 |
| 282 | ZINC000215185695 | -17.5 | 3.1 | -47.4 | 2.1 | -15.8 | 2.1 | 52.2 | 2.9 | -6.5 | 0.1 |
| 283 | ZINC000012508838 | -17.1 | 4.7 | -49.0 | 2.3 | -24.7 | 4.2 | 62.5 | 5.2 | -5.9 | 0.1 |
| 284 | ZINC000006556210 | -17.1 | 4.0 | -41.4 | 2.6 | -10.2 | 2.5 | 39.9 | 5.0 | -5.4 | 0.1 |
| 285 | ZINC000012142779 | -16.9 | 5.8 | -50.2 | 2.6 | -15.4 | 9.7 | 55.2 | 7.6 | -6.4 | 0.2 |
| 286 | ZINC000034407910 | -16.8 | 3.6 | -38.4 | 2.1 | -16.2 | 2.9 | 43.1 | 3.4 | -5.2 | 0.1 |
| 287 | ZINC000255187536 | -16.7 | 3.9 | -42.3 | 2.5 | -21.5 | 3.7 | 52.6 | 3.7 | -5.5 | 0.1 |
| 288 | ZINC000268943077 | -16.4 | 4.4 | -41.8 | 2.6 | -25.1 | 8.3 | 56.3 | 7.7 | -5.9 | 0.1 |
| 289 | ZINC000170016387 | -16.3 | 3.7 | -42.6 | 2.8 | -20.5 | 5.3 | 53.0 | 5.0 | -6.2 | 0.1 |
| 290 | ZINC000012557330 | -16.1 | 4.7 | -43.0 | 2.5 | -10.2 | 4.3 | 42.6 | 5.3 | -5.6 | 0.1 |
| 291 | ZINC000238831563 | -15.8 | 4.7 | -47.8 | 2.5 | -11.5 | 4.4 | 49.2 | 4.0 | -5.6 | 0.1 |
| 292 | ZINC000004807849 | -15.4 | 5.2 | -36.5 | 3.5 | -47.7 | 6.0 | 73.9 | 5.1 | -5.1 | 0.1 |
| 293 | ZINC000284798034 | -15.2 | 4.4 | -50.9 | 2.3 | -22.1 | 4.5 | 63.5 | 5.1 | -5.7 | 0.1 |
| 294 | ZINC000013278475 | -15.1 | 5.3 | -48.6 | 2.9 | -43.7 | 10.1 | 83.1 | 10.3 | -5.9 | 0.2 |
| 295 | ZINC000061067795 | -15.1 | 4.2 | -48.3 | 2.2 | -15.0 | 3.9 | 54.2 | 5.0 | -5.8 | 0.1 |
| 296 | ZINC000018177963 | -13.5 | 3.6 | -32.7 | 3.1 | -55.0 | 5.3 | 79.4 | 4.3 | -5.2 | 0.1 |
| 297 | ZINC000000201272 | -13.1 | 4.7 | -46.5 | 2.2 | -6.7 | 5.2 | 45.6 | 4.2 | -5.7 | 0.1 |
| 298 | ZINC000006576136 | -12.8 | 5.0 | -34.5 | 2.6 | -32.9 | 10.1 | 59.9 | 8.1 | -5.3 | 0.1 |
| 299 | ZINC000237699092 | -11.6 | 6.4 | -40.8 | 3.4 | -32.2 | 15.3 | 67.7 | 15.9 | -6.2 | 0.1 |
| 300 | ZINC000000353960 | -10.4 | 7.0 | -35.6 | 2.7 | -63.0 | 13.2 | 93.5 | 11.7 | -5.3 | 0.1 |
| 301 | ZINC000096198984 | -10.0 | 5.0 | -45.9 | 2.9 | -8.7 | 3.7 | 51.0 | 5.6 | -6.4 | 0.2 |
| 302 | ZINC000004806959 | -9.3 | 5.2 | -39.4 | 2.6 | -55.4 | 9.5 | 91.1 | 8.7 | -5.5 | 0.1 |
| 303 | ZINC000069895275 | -9.0 | 4.8 | -37.0 | 2.2 | -35.4 | 13.7 | 68.8 | 12.0 | -5.3 | 0.1 |
| 304 | ZINC000003947438 | -8.7 | 5.4 | -41.0 | 2.4 | -48.7 | 9.2 | 87.2 | 9.1 | -6.1 | 0.1 |
| 305 | ZINC000008848147 | -5.4 | 4.9 | -38.7 | 3.2 | -26.7 | 14.3 | 65.4 | 12.0 | -5.4 | 0.1 |

**Table S5.** Energy decomposition of the MM/GBSA results for the 305 compounds using the *GB-1* parameter set (for more details see the Methods section). All energy terms are given in kcal/mol with their standard deviation (*std*) and are sorted by the binding free energy ($\Delta G_{bind}$) followed by the molecular mechanics van der Waals and electrostatic interactions terms ($\Delta E_{vdW}$ and $\Delta E_{el}$) and the polar and non-polar contributions to the solvation free energy ($\Delta G_{pol}$ and $\Delta G_{np}$). Compounds marked in boldface were finally selected for experimental screening.

| # | ZINC ID | $\Delta G_{bind}$ | *std* | $\Delta E_{vdW}$ | *std* | $\Delta E_{el}$ | *std* | $\Delta G_{pol}$ | *std* | $\Delta G_{np}$ | *std* |
|---|---|---|---|---|---|---|---|---|---|---|---|
| 1 | ZINC000257317306 | -56.2 | 2.9 | -50.1 | 2.8 | -41.2 | 3.3 | 41.5 | 2.0 | -6.5 | 0.2 |
| **2** | **ZINC000035869910** | **-55.5** | **3.3** | **-54.6** | **2.6** | **-34.9** | **5.2** | **40.7** | **2.8** | **-6.7** | **0.1** |
| **3** | **ZINC000010256259** | **-53.6** | **2.8** | **-52.7** | **2.7** | **-41.4** | **3.7** | **46.9** | **2.5** | **-6.4** | **0.1** |
| **4** | **ZINC000069778215** | **-53.5** | **3.0** | **-47.6** | **2.7** | **-42.2** | **4.2** | **42.6** | **2.2** | **-6.3** | **0.1** |
| **5** | **ZINC000091270543** | **-53.3** | **2.7** | **-53.6** | **3.0** | **-21.4** | **3.7** | **28.5** | **2.4** | **-6.7** | **0.1** |
| **6** | **ZINC000022191233** | **-53.1** | **2.7** | **-53.6** | **2.9** | **-30.9** | **3.7** | **38.1** | **2.5** | **-6.6** | **0.1** |
| **7** | **ZINC000004998502** | **-52.8** | **3.3** | **-53.5** | **3.3** | **-40.0** | **4.2** | **47.1** | **2.7** | **-6.3** | **0.2** |
| **8** | **ZINC000003463362** | **-52.6** | **2.8** | **-50.0** | **2.8** | **-30.0** | **4.2** | **33.8** | **2.8** | **-6.4** | **0.2** |
| **9** | **ZINC000095415912** | **-52.6** | **3.3** | **-53.2** | **2.7** | **-29.2** | **4.2** | **36.1** | **2.6** | **-6.3** | **0.1** |
| 10 | ZINC000091486946 | -52.2 | 2.6 | -52.4 | 2.6 | -21.3 | 2.8 | 28.1 | 2.3 | -6.7 | 0.2 |
| 11 | ZINC000092022499 | -51.3 | 2.4 | -53.4 | 2.6 | -23.1 | 2.7 | 31.7 | 1.8 | -6.6 | 0.1 |
| **12** | **ZINC000084578820** | **-51.1** | **3.2** | **-47.8** | **2.6** | **-35.2** | **7.0** | **37.8** | **3.8** | **-6.0** | **0.1** |
| **13** | **ZINC000091486133** | **-51.0** | **2.8** | **-52.7** | **2.9** | **-25.9** | **3.2** | **34.3** | **2.2** | **-6.6** | **0.2** |
| 14 | ZINC000075530913 | -50.9 | 2.2 | -49.2 | 2.3 | -20.2 | 2.3 | 24.7 | 1.5 | -6.2 | 0.1 |
| 15 | ZINC000096058349 | -50.5 | 2.6 | -54.1 | 2.4 | -18.0 | 2.3 | 28.1 | 1.4 | -6.5 | 0.1 |
| **16** | **ZINC000023642159** | **-50.5** | **2.9** | **-47.6** | **2.3** | **-27.3** | **4.0** | **30.2** | **2.0** | **-5.8** | **0.2** |
| 17 | ZINC000091270544 | -50.0 | 2.7 | -52.4 | 2.7 | -22.1 | 3.8 | 31.0 | 2.4 | -6.5 | 0.2 |
| **18** | **ZINC000057563200** | **-50.0** | **3.1** | **-49.9** | **2.7** | **-17.6** | **4.6** | **23.4** | **2.1** | **-6.0** | **0.2** |
| **19** | **ZINC000000333210** | **-49.8** | **2.7** | **-50.6** | **3.0** | **-32.9** | **3.7** | **39.8** | **2.2** | **-6.1** | **0.1** |
| 20 | ZINC000057731402 | -49.7 | 2.9 | -51.2 | 2.6 | -25.2 | 3.7 | 33.0 | 2.1 | -6.3 | 0.1 |
| 21 | ZINC000092065700 | -49.7 | 2.6 | -52.6 | 2.5 | -19.8 | 2.9 | 28.9 | 2.1 | -6.1 | 0.2 |
| **22** | **ZINC000025032259** | **-49.1** | **2.9** | **-54.4** | **2.7** | **-21.9** | **3.2** | **33.6** | **2.3** | **-6.4** | **0.2** |
| **23** | **ZINC000008913866** | **-49.1** | **3.4** | **-50.7** | **2.5** | **-28.3** | **6.0** | **36.3** | **4.0** | **-6.4** | **0.1** |
| **24** | **ZINC000005360885** | **-49.0** | **3.3** | **-48.1** | **2.7** | **-36.4** | **4.9** | **41.1** | **2.4** | **-5.6** | **0.1** |
| **25** | **ZINC000002216953** | **-49.0** | **2.5** | **-48.7** | **2.4** | **-24.8** | **3.2** | **30.6** | **2.2** | **-6.2** | **0.1** |
| **26** | **ZINC000084598177** | **-49.0** | **2.5** | **-49.0** | **2.5** | **-30.6** | **3.3** | **36.3** | **2.4** | **-5.8** | **0.2** |
| **27** | **ZINC000015078577** | **-49.0** | **2.8** | **-49.4** | **2.8** | **-29.1** | **4.5** | **35.8** | **3.8** | **-6.3** | **0.2** |
| 28 | ZINC000230113696 | -48.9 | 2.8 | -52.9 | 2.8 | -22.8 | 4.3 | 33.0 | 3.3 | -6.2 | 0.1 |
| 29 | ZINC000334160460 | -48.7 | 2.5 | -49.1 | 2.5 | -22.2 | 2.9 | 28.7 | 1.6 | -6.1 | 0.1 |
| 30 | ZINC000012142783 | -48.6 | 3.1 | -51.0 | 2.5 | -21.5 | 5.1 | 30.1 | 4.3 | -6.2 | 0.2 |
| **31** | **ZINC000051467674** | **-48.6** | **2.6** | **-46.6** | **2.7** | **-40.6** | **3.8** | **45.0** | **2.3** | **-6.4** | **0.1** |
| **32** | **ZINC000079491038** | **-48.5** | **2.9** | **-51.3** | **2.7** | **-27.7** | **5.0** | **37.0** | **3.8** | **-6.5** | **0.1** |
| 33 | ZINC000257299555 | -48.4 | 2.7 | -46.7 | 3.1 | -36.0 | 4.2 | 40.5 | 3.0 | -6.2 | 0.1 |
| 34 | ZINC000095950700 | -48.4 | 2.4 | -48.8 | 2.7 | -19.4 | 2.1 | 25.9 | 1.8 | -6.0 | 0.2 |
| 35 | ZINC000096158052 | -48.3 | 2.7 | -49.9 | 2.5 | -20.8 | 3.9 | 28.6 | 2.8 | -6.2 | 0.2 |
| 36 | ZINC000091768990 | -48.2 | 2.6 | -52.4 | 2.8 | -13.3 | 2.6 | 23.8 | 2.0 | -6.3 | 0.1 |
| **37** | **ZINC000018148904** | **-48.2** | **2.9** | **-50.1** | **2.6** | **-32.7** | **3.6** | **40.4** | **2.3** | **-5.8** | **0.1** |
| **38** | **ZINC000095410774** | **-48.2** | **2.5** | **-50.5** | **2.4** | **-22.8** | **3.0** | **31.3** | **2.2** | **-6.1** | **0.1** |

| # | ZINC ID | $\Delta G_{bind}$ | *std* | $\Delta E_{vdW}$ | *std* | $\Delta E_{el}$ | *std* | $\Delta G_{pol}$ | *std* | $\Delta G_{np}$ | *std* |
|---|---|---|---|---|---|---|---|---|---|---|---|
| 39 | ZINC000575618196 | -48.1 | 2.8 | -54.5 | 2.7 | -27.7 | 3.9 | 40.6 | 2.5 | -6.4 | 0.1 |
| 40 | ZINC000195547314 | -47.9 | 2.8 | -50.9 | 2.8 | -13.5 | 3.3 | 22.8 | 2.0 | -6.3 | 0.2 |
| 41 | ZINC000072290753 | -47.8 | 3.1 | -52.3 | 2.4 | -26.0 | 4.2 | 36.6 | 2.7 | -6.1 | 0.1 |
| 42 | ZINC000229623928 | -47.7 | 2.5 | -50.6 | 2.5 | -34.1 | 3.8 | 43.3 | 3.0 | -6.3 | 0.1 |
| 43 | ZINC000065395709 | -47.7 | 2.6 | -51.6 | 2.4 | -22.3 | 3.1 | 32.8 | 2.0 | -6.6 | 0.1 |
| 44 | ZINC000034895992 | -47.2 | 3.0 | -49.7 | 2.7 | -29.9 | 3.5 | 38.7 | 2.4 | -6.2 | 0.2 |
| 45 | ZINC000002646979 | -47.2 | 2.6 | -51.5 | 2.3 | -22.3 | 3.2 | 32.8 | 2.4 | -6.3 | 0.1 |
| 46 | ZINC000096199614 | -47.0 | 2.9 | -51.3 | 2.5 | -27.5 | 3.7 | 37.5 | 2.2 | -5.7 | 0.1 |
| 47 | ZINC000100193430 | -46.9 | 2.1 | -47.9 | 2.4 | -13.6 | 2.7 | 20.0 | 1.8 | -5.3 | 0.1 |
| **48** | **ZINC000069562405** | **-46.8** | **2.5** | **-47.4** | **2.6** | **-25.3** | **2.9** | **31.8** | **2.0** | **-5.9** | **0.1** |
| 49 | ZINC000069846908 | -46.7 | 3.4 | -50.0 | 3.0 | -33.8 | 7.9 | 43.2 | 5.3 | -6.1 | 0.2 |
| **50** | **ZINC000008595944** | **-46.7** | **2.6** | **-53.1** | **2.3** | **-13.9** | **4.1** | **26.2** | **3.0** | **-5.9** | **0.1** |
| 51 | ZINC000100193536 | -46.6 | 2.7 | -48.7 | 2.9 | -15.8 | 2.9 | 23.4 | 2.1 | -5.5 | 0.2 |
| 52 | ZINC000089827813 | -46.6 | 3.3 | -46.6 | 3.2 | -28.8 | 4.7 | 34.5 | 2.5 | -5.8 | 0.2 |
| 53 | ZINC000064871452 | -46.5 | 3.2 | -49.9 | 2.5 | -27.1 | 7.3 | 36.8 | 5.2 | -6.2 | 0.2 |
| 54 | ZINC000585280336 | -46.5 | 3.8 | -51.7 | 2.3 | -30.1 | 5.9 | 41.7 | 3.5 | -6.2 | 0.1 |
| 55 | ZINC000075118384 | -46.4 | 3.4 | -46.9 | 2.9 | -33.8 | 7.2 | 40.3 | 4.1 | -5.9 | 0.2 |
| 56 | ZINC000003641308 | -46.3 | 5.2 | -45.0 | 2.6 | -25.7 | 7.4 | 30.1 | 4.1 | -5.7 | 0.2 |
| 57 | ZINC000257211885 | -46.3 | 4.2 | -50.3 | 3.9 | -20.9 | 3.8 | 30.5 | 2.4 | -5.6 | 0.2 |
| **58** | **ZINC000069737923** | **-46.2** | **2.8** | **-46.3** | **2.3** | **-29.6** | **4.0** | **35.8** | **2.5** | **-6.1** | **0.1** |
| 59 | ZINC000257218603 | -46.2 | 2.8 | -50.2 | 2.5 | -24.7 | 5.1 | 35.1 | 3.3 | -6.4 | 0.1 |
| **60** | **ZINC000071915224** | **-46.2** | **2.6** | **-49.3** | **2.3** | **-26.0** | **3.5** | **35.1** | **2.6** | **-5.9** | **0.1** |
| **61** | **ZINC000223692657** | **-46.0** | **2.6** | **-50.8** | **2.6** | **-27.8** | **4.0** | **38.5** | **2.4** | **-6.0** | **0.1** |
| 62 | ZINC000096238948 | -46.0 | 3.0 | -53.8 | 2.7 | -13.0 | 5.3 | 27.1 | 4.4 | -6.2 | 0.3 |
| 63 | ZINC000007030710 | -46.0 | 3.0 | -47.3 | 2.7 | -24.4 | 4.2 | 31.4 | 2.7 | -5.6 | 0.1 |
| **64** | **ZINC000043550595** | **-45.9** | **2.7** | **-48.3** | **2.5** | **-29.1** | **3.5** | **37.7** | **2.0** | **-6.2** | **0.2** |
| 65 | ZINC000096251711 | -45.8 | 3.3 | -50.9 | 3.0 | -15.0 | 3.5 | 26.4 | 2.1 | -6.2 | 0.2 |
| 66 | ZINC000013692677 | -45.8 | 3.8 | -51.9 | 3.1 | -30.8 | 4.7 | 43.5 | 3.3 | -6.6 | 0.2 |
| 67 | ZINC000257192562 | -45.7 | 2.1 | -45.9 | 2.2 | -16.4 | 2.3 | 22.5 | 1.6 | -5.9 | 0.2 |
| 68 | ZINC000171342968 | -45.7 | 3.2 | -48.4 | 2.7 | -39.2 | 3.9 | 48.3 | 2.8 | -6.4 | 0.1 |
| 69 | ZINC000194368147 | -45.7 | 2.7 | -48.3 | 2.8 | -30.0 | 3.5 | 38.9 | 2.1 | -6.4 | 0.1 |
| 70 | ZINC000225746732 | -45.7 | 2.2 | -47.5 | 2.3 | -20.8 | 2.3 | 28.0 | 1.9 | -5.4 | 0.2 |
| 71 | ZINC000100737122 | -45.6 | 2.5 | -52.3 | 2.5 | -22.5 | 3.1 | 35.4 | 2.5 | -6.2 | 0.1 |
| 72 | ZINC000278659999 | -45.5 | 2.5 | -47.8 | 2.4 | -23.7 | 3.5 | 31.9 | 2.0 | -6.0 | 0.2 |
| 73 | ZINC000225489282 | -45.5 | 2.8 | -49.8 | 2.4 | -16.6 | 4.6 | 26.9 | 3.3 | -6.1 | 0.1 |
| 74 | ZINC000257206611 | -45.4 | 2.9 | -55.3 | 3.1 | -11.4 | 5.7 | 27.7 | 4.5 | -6.3 | 0.2 |
| 75 | ZINC000095503625 | -45.4 | 3.4 | -50.6 | 2.6 | -24.3 | 4.9 | 36.1 | 3.4 | -6.6 | 0.2 |
| 76 | ZINC000067460397 | -45.4 | 2.3 | -50.1 | 2.2 | -35.5 | 3.5 | 46.1 | 2.6 | -5.8 | 0.1 |
| 77 | ZINC000004722089 | -45.3 | 2.2 | -48.1 | 2.5 | -14.4 | 2.7 | 22.5 | 1.7 | -5.3 | 0.1 |
| 78 | ZINC000071282106 | -45.3 | 3.2 | -47.7 | 2.6 | -15.4 | 4.9 | 24.0 | 3.0 | -6.2 | 0.2 |
| 79 | ZINC000097413355 | -45.3 | 2.1 | -46.7 | 2.6 | -27.5 | 3.2 | 34.5 | 2.2 | -5.7 | 0.1 |
| 80 | ZINC000409428381 | -45.3 | 3.1 | -51.5 | 2.4 | -12.2 | 4.9 | 25.0 | 3.4 | -6.6 | 0.1 |
| 81 | ZINC000092760184 | -45.2 | 2.5 | -47.5 | 2.4 | -20.1 | 3.4 | 28.6 | 2.3 | -6.2 | 0.2 |
| 82 | ZINC000002922856 | -45.2 | 3.1 | -52.4 | 2.7 | -15.5 | 3.6 | 29.0 | 2.6 | -6.2 | 0.1 |
| **83** | **ZINC000004511530** | **-45.2** | **2.4** | **-49.2** | **2.1** | **-22.6** | **3.0** | **32.9** | **2.1** | **-6.3** | **0.1** |
| 84 | ZINC000097201269 | -45.1 | 3.3 | -49.4 | 2.6 | -16.9 | 4.3 | 27.7 | 2.7 | -6.4 | 0.3 |

| # | ZINC ID | Δ$G_{bind}$ | *std* | Δ$E_{vdW}$ | *std* | Δ$E_{el}$ | *std* | Δ$G_{pol}$ | *std* | Δ$G_{np}$ | *std* |
|---|---|---|---|---|---|---|---|---|---|---|---|
| 85 | ZINC000005426253 | -45.1 | 2.4 | -49.8 | 2.4 | -11.6 | 3.2 | 22.9 | 2.9 | -6.5 | 0.1 |
| 86 | ZINC000003292603 | -45.1 | 2.6 | -47.2 | 2.4 | -19.3 | 3.6 | 27.5 | 2.6 | -6.1 | 0.2 |
| 87 | ZINC000014791001 | -45.1 | 2.6 | -47.0 | 2.3 | -21.3 | 3.4 | 29.3 | 2.8 | -6.1 | 0.1 |
| 88 | ZINC000306141880 | -45.0 | 2.6 | -46.5 | 2.8 | -29.1 | 4.2 | 36.6 | 3.0 | -6.0 | 0.1 |
| 89 | ZINC000096003964 | -45.0 | 3.5 | -49.1 | 2.6 | -26.2 | 4.8 | 36.3 | 2.3 | -5.9 | 0.1 |
| 90 | ZINC000170800517 | -44.9 | 2.3 | -49.4 | 2.4 | -18.5 | 2.4 | 29.5 | 1.8 | -6.5 | 0.2 |
| 91 | ZINC000009338249 | -44.9 | 3.9 | -47.0 | 2.5 | -27.4 | 4.8 | 35.2 | 3.0 | -5.6 | 0.1 |
| 92 | ZINC000257310083 | -44.8 | 2.5 | -47.2 | 2.6 | -16.6 | 2.4 | 24.4 | 1.6 | -5.4 | 0.1 |
| 93 | ZINC000000489138 | -44.7 | 2.7 | -50.3 | 2.3 | -20.6 | 3.0 | 32.2 | 2.0 | -6.1 | 0.1 |
| 94 | ZINC000091664087 | -44.7 | 2.9 | -54.4 | 2.3 | -7.8 | 3.5 | 23.9 | 2.3 | -6.5 | 0.1 |
| 95 | ZINC000095393415 | -44.7 | 2.7 | -54.8 | 2.5 | -14.5 | 3.2 | 30.3 | 2.7 | -5.8 | 0.1 |
| 96 | ZINC000084686490 | -44.7 | 2.4 | -46.1 | 2.4 | -33.1 | 3.1 | 40.6 | 2.0 | -6.1 | 0.1 |
| 97 | ZINC000019328346 | -44.7 | 7.4 | -46.7 | 4.6 | -22.0 | 4.5 | 30.0 | 2.2 | -5.9 | 0.2 |
| 98 | ZINC000013973188 | -44.7 | 2.5 | -47.2 | 2.5 | -29.6 | 3.6 | 38.1 | 2.5 | -6.0 | 0.1 |
| 99 | ZINC000040473994 | -44.6 | 2.5 | -49.6 | 2.8 | -26.3 | 4.0 | 37.1 | 2.4 | -5.9 | 0.1 |
| 100 | ZINC000012323932 | -44.6 | 3.3 | -42.7 | 3.2 | -38.0 | 7.3 | 41.7 | 4.2 | -5.6 | 0.2 |
| 101 | ZINC000079001413 | -44.5 | 2.5 | -52.9 | 2.5 | -9.1 | 2.5 | 23.7 | 1.9 | -6.2 | 0.2 |
| 102 | ZINC000238886413 | -44.3 | 3.1 | -49.4 | 2.7 | -11.6 | 3.3 | 22.6 | 1.5 | -5.9 | 0.2 |
| **103** | **ZINC000240454699** | **-44.3** | **2.5** | **-42.5** | **2.5** | **-24.0** | **4.4** | **28.0** | **3.1** | **-5.8** | **0.1** |
| 104 | ZINC000022549345 | -44.3 | 2.1 | -53.9 | 2.1 | -18.2 | 3.8 | 33.7 | 3.6 | -5.9 | 0.1 |
| 105 | ZINC000238121810 | -44.3 | 3.4 | -37.2 | 2.9 | -88.8 | 10.4 | 87.6 | 8.6 | -6.0 | 0.2 |
| 106 | ZINC000029134571 | -44.3 | 2.4 | -52.1 | 2.4 | -19.6 | 3.3 | 33.5 | 2.6 | -6.1 | 0.1 |
| 107 | ZINC000069349055 | -44.2 | 3.4 | -48.3 | 3.0 | -24.2 | 8.4 | 34.5 | 5.5 | -6.2 | 0.2 |
| 108 | ZINC000011781217 | -44.2 | 2.6 | -52.0 | 2.2 | -13.2 | 2.4 | 27.3 | 2.0 | -6.2 | 0.2 |
| 109 | ZINC000100191495 | -44.2 | 2.7 | -46.6 | 2.6 | -14.2 | 2.7 | 22.0 | 1.9 | -5.4 | 0.1 |
| 110 | ZINC000328587242 | -44.2 | 2.4 | -45.2 | 2.7 | -23.7 | 2.6 | 30.9 | 1.8 | -6.1 | 0.2 |
| 111 | ZINC000071497775 | -44.2 | 3.2 | -51.3 | 2.3 | -25.4 | 6.7 | 39.0 | 4.7 | -6.4 | 0.2 |
| 112 | ZINC000065248686 | -44.1 | 2.5 | -45.7 | 2.6 | -14.1 | 2.7 | 21.5 | 1.5 | -5.9 | 0.1 |
| 113 | ZINC000069775761 | -44.1 | 3.5 | -48.1 | 2.6 | -21.8 | 4.1 | 31.7 | 1.9 | -5.9 | 0.1 |
| 114 | ZINC000216315534 | -44.1 | 2.4 | -41.9 | 2.5 | -27.2 | 4.7 | 30.6 | 3.3 | -5.6 | 0.1 |
| 115 | ZINC000096069207 | -44.0 | 2.8 | -52.2 | 2.8 | -20.5 | 2.9 | 35.0 | 2.2 | -6.4 | 0.2 |
| 116 | ZINC000050584912 | -44.0 | 3.3 | -50.3 | 2.8 | -6.3 | 3.9 | 18.6 | 3.7 | -6.1 | 0.3 |
| 117 | ZINC000002465084 | -43.8 | 3.0 | -49.8 | 2.5 | -23.7 | 3.3 | 35.8 | 2.4 | -6.1 | 0.1 |
| 118 | ZINC000003438521 | -43.8 | 2.5 | -45.4 | 2.3 | -22.7 | 4.5 | 29.9 | 2.8 | -5.7 | 0.1 |
| 119 | ZINC000021835963 | -43.8 | 2.5 | -48.7 | 2.2 | -26.2 | 3.3 | 36.9 | 2.0 | -5.8 | 0.1 |
| 120 | ZINC000013691849 | -43.6 | 3.3 | -44.2 | 3.0 | -33.9 | 4.8 | 40.2 | 2.7 | -5.6 | 0.2 |
| 121 | ZINC000072290711 | -43.6 | 2.6 | -46.7 | 2.9 | -16.6 | 3.3 | 25.0 | 2.3 | -5.3 | 0.2 |
| 122 | ZINC000091304527 | -43.6 | 2.8 | -50.9 | 2.5 | -14.0 | 4.5 | 27.5 | 3.3 | -6.2 | 0.2 |
| 123 | ZINC000077503523 | -43.6 | 2.7 | -45.1 | 2.9 | -34.1 | 6.9 | 41.4 | 4.9 | -5.7 | 0.2 |
| 124 | ZINC000092078469 | -43.5 | 3.7 | -45.0 | 3.2 | -23.4 | 3.5 | 30.3 | 2.7 | -5.5 | 0.3 |
| 125 | ZINC000096251709 | -43.5 | 2.6 | -48.0 | 2.1 | -18.9 | 3.2 | 29.2 | 1.8 | -5.8 | 0.2 |
| 126 | ZINC000008316344 | -43.5 | 2.5 | -51.0 | 2.4 | -4.6 | 2.6 | 18.0 | 2.0 | -5.9 | 0.2 |
| **127** | **ZINC000244816835** | **-43.5** | **2.7** | **-43.8** | **3.0** | **-34.1** | **3.6** | **40.2** | **2.0** | **-5.8** | **0.1** |
| 128 | ZINC000253433259 | -43.4 | 2.3 | -49.1 | 2.2 | -19.7 | 2.9 | 31.2 | 2.3 | -5.8 | 0.1 |
| 129 | ZINC000072096437 | -43.4 | 2.5 | -50.9 | 2.4 | -18.3 | 4.2 | 32.5 | 3.0 | -6.7 | 0.1 |
| 130 | ZINC000069492530 | -43.4 | 2.9 | -47.5 | 2.6 | -19.0 | 3.3 | 28.6 | 1.9 | -5.5 | 0.2 |

| # | ZINC ID | Δ$G_{bind}$ | *std* | Δ$E_{vdW}$ | *std* | Δ$E_{el}$ | *std* | Δ$G_{pol}$ | *std* | Δ$G_{np}$ | *std* |
|---|---|---|---|---|---|---|---|---|---|---|---|
| 131 | ZINC000257222272 | -43.4 | 2.5 | -51.4 | 2.2 | -7.3 | 2.1 | 21.6 | 1.6 | -6.3 | 0.1 |
| 132 | ZINC000028790813 | -43.4 | 3.4 | -51.7 | 2.7 | -24.1 | 5.5 | 38.4 | 3.6 | -5.9 | 0.2 |
| 133 | ZINC000065167646 | -43.3 | 3.1 | -47.8 | 2.7 | -21.8 | 4.0 | 32.3 | 3.4 | -6.0 | 0.1 |
| 134 | ZINC000044908085 | -43.3 | 3.2 | -51.1 | 2.7 | -21.0 | 3.5 | 34.5 | 2.7 | -5.7 | 0.2 |
| **135** | **ZINC000044919578** | **-43.2** | **2.1** | **-43.0** | **2.1** | **-29.0** | **3.4** | **34.2** | **2.1** | **-5.5** | **0.1** |
| 136 | ZINC000257256038 | -43.2 | 2.3 | -45.9 | 2.4 | -24.2 | 3.1 | 33.2 | 1.8 | -6.2 | 0.1 |
| 137 | ZINC000072300733 | -43.0 | 3.7 | -51.3 | 2.7 | -12.8 | 4.4 | 27.3 | 2.9 | -6.2 | 0.2 |
| 138 | ZINC000092021781 | -43.0 | 2.9 | -49.3 | 2.9 | -20.5 | 5.0 | 33.0 | 3.5 | -6.1 | 0.1 |
| 139 | ZINC000006669352 | -42.9 | 2.5 | -47.6 | 2.2 | -23.0 | 5.5 | 34.0 | 4.2 | -6.3 | 0.1 |
| 140 | ZINC000004495846 | -42.9 | 2.7 | -46.3 | 2.7 | -26.6 | 4.2 | 35.6 | 3.5 | -5.8 | 0.2 |
| 141 | ZINC000584887110 | -42.9 | 2.3 | -46.9 | 2.2 | -14.2 | 2.8 | 23.8 | 2.0 | -5.7 | 0.1 |
| 142 | ZINC000044874685 | -42.8 | 2.9 | -44.2 | 2.8 | -22.4 | 4.6 | 29.5 | 3.6 | -5.7 | 0.2 |
| 143 | ZINC000052955795 | -42.8 | 2.8 | -46.7 | 2.6 | -27.1 | 4.8 | 37.0 | 2.9 | -6.0 | 0.1 |
| 144 | ZINC000072437791 | -42.7 | 2.1 | -46.5 | 2.2 | -12.5 | 3.0 | 22.2 | 2.0 | -5.9 | 0.1 |
| 145 | ZINC000012550491 | -42.7 | 2.6 | -46.4 | 2.6 | -23.5 | 3.5 | 33.0 | 2.2 | -5.7 | 0.1 |
| 146 | ZINC000035398834 | -42.5 | 2.3 | -51.5 | 2.3 | -9.7 | 2.6 | 24.6 | 2.1 | -6.0 | 0.1 |
| 147 | ZINC000135810445 | -42.5 | 2.9 | -44.5 | 2.6 | -31.9 | 5.3 | 39.8 | 3.6 | -5.9 | 0.2 |
| 148 | ZINC000002750765 | -42.5 | 2.4 | -45.0 | 2.4 | -24.4 | 3.0 | 32.8 | 2.3 | -6.0 | 0.1 |
| 149 | ZINC000065463577 | -42.5 | 2.6 | -46.6 | 2.7 | -32.2 | 5.4 | 42.3 | 4.2 | -5.9 | 0.1 |
| 150 | ZINC000584897275 | -42.3 | 2.6 | -44.7 | 2.8 | -36.7 | 4.3 | 44.8 | 3.0 | -5.7 | 0.1 |
| 151 | ZINC000057526426 | -42.3 | 2.5 | -44.4 | 2.2 | -27.1 | 3.1 | 35.0 | 2.4 | -5.7 | 0.1 |
| 152 | ZINC000040548686 | -42.2 | 2.5 | -51.5 | 2.2 | -23.2 | 4.2 | 38.5 | 2.7 | -6.0 | 0.1 |
| 153 | ZINC000072235028 | -42.1 | 2.6 | -52.8 | 2.4 | -12.4 | 4.3 | 29.4 | 3.0 | -6.3 | 0.1 |
| 154 | ZINC000095469884 | -42.1 | 2.6 | -40.0 | 2.8 | -35.7 | 3.7 | 38.6 | 1.9 | -5.0 | 0.1 |
| 155 | ZINC000100119126 | -42.0 | 3.1 | -47.3 | 2.8 | -19.9 | 4.4 | 31.2 | 3.0 | -6.0 | 0.2 |
| 156 | ZINC000006887927 | -42.0 | 2.5 | -48.4 | 2.6 | -23.8 | 3.7 | 36.4 | 2.4 | -6.3 | 0.1 |
| 157 | ZINC000072290626 | -42.0 | 2.6 | -48.7 | 2.2 | -19.4 | 5.3 | 31.8 | 4.4 | -5.7 | 0.1 |
| 158 | ZINC000050857093 | -42.0 | 3.6 | -48.7 | 2.5 | -25.4 | 3.8 | 38.4 | 2.7 | -6.2 | 0.1 |
| 159 | ZINC000119429896 | -41.9 | 2.4 | -49.5 | 2.2 | -9.9 | 2.9 | 23.8 | 2.2 | -6.3 | 0.2 |
| 160 | ZINC000069595091 | -41.9 | 2.9 | -45.8 | 2.8 | -22.4 | 3.9 | 32.2 | 3.1 | -6.0 | 0.2 |
| 161 | ZINC000096010936 | -41.9 | 2.6 | -41.4 | 2.8 | -26.7 | 3.3 | 32.2 | 1.9 | -5.9 | 0.1 |
| 162 | ZINC000040060202 | -41.8 | 2.6 | -46.1 | 2.8 | -17.7 | 3.4 | 27.9 | 2.4 | -6.0 | 0.2 |
| 163 | ZINC000095447759 | -41.8 | 3.5 | -46.9 | 2.9 | -24.8 | 5.5 | 35.6 | 3.3 | -5.8 | 0.1 |
| 164 | ZINC000057771728 | -41.8 | 3.3 | -48.2 | 2.5 | -24.0 | 5.5 | 36.6 | 3.4 | -6.2 | 0.3 |
| 165 | ZINC000068269627 | -41.7 | 2.5 | -50.4 | 2.4 | -27.6 | 4.4 | 42.2 | 4.0 | -5.9 | 0.2 |
| 166 | ZINC000012888207 | -41.7 | 2.1 | -40.8 | 2.2 | -27.0 | 3.4 | 31.2 | 2.2 | -5.1 | 0.1 |
| 167 | ZINC000050859891 | -41.6 | 3.3 | -48.4 | 2.6 | -29.5 | 4.7 | 42.1 | 3.6 | -5.9 | 0.2 |
| 168 | ZINC000074014922 | -41.6 | 2.2 | -47.0 | 2.1 | -9.6 | 2.2 | 20.6 | 1.9 | -5.7 | 0.1 |
| 169 | ZINC000040386093 | -41.6 | 2.8 | -45.0 | 2.7 | -13.8 | 2.9 | 22.7 | 2.4 | -5.4 | 0.2 |
| 170 | ZINC000071746202 | -41.5 | 2.8 | -47.6 | 2.6 | -15.1 | 3.6 | 27.3 | 2.6 | -6.1 | 0.2 |
| 171 | ZINC000012142779 | -41.5 | 4.4 | -50.2 | 2.6 | -15.4 | 9.4 | 30.5 | 6.4 | -6.4 | 0.2 |
| 172 | ZINC000005244779 | -41.4 | 2.4 | -49.8 | 2.4 | -9.7 | 2.7 | 24.1 | 2.4 | -6.1 | 0.1 |
| 173 | ZINC000096024249 | -41.4 | 2.9 | -47.4 | 2.4 | -18.1 | 5.1 | 29.4 | 3.4 | -5.3 | 0.2 |
| 174 | ZINC000016228943 | -41.4 | 2.5 | -43.6 | 2.6 | -26.3 | 3.4 | 33.9 | 2.1 | -5.4 | 0.2 |
| 175 | ZINC000245171655 | -41.4 | 2.4 | -48.7 | 2.3 | -10.2 | 2.7 | 23.3 | 1.8 | -5.8 | 0.2 |
| 176 | ZINC000022241454 | -41.4 | 3.2 | -39.1 | 2.9 | -38.4 | 6.7 | 41.0 | 5.3 | -5.0 | 0.2 |

| # | ZINC ID | $\Delta G_{bind}$ | *std* | $\Delta E_{vdW}$ | *std* | $\Delta E_{el}$ | *std* | $\Delta G_{pol}$ | *std* | $\Delta G_{np}$ | *std* |
|---|---|---|---|---|---|---|---|---|---|---|---|
| 177 | ZINC000062639858 | -41.2 | 2.4 | -52.0 | 2.2 | -3.0 | 3.4 | 20.1 | 2.8 | -6.4 | 0.2 |
| 178 | ZINC000008771448 | -41.2 | 4.1 | -48.5 | 3.0 | -23.5 | 6.0 | 36.6 | 4.8 | -5.8 | 0.4 |
| 179 | ZINC000009318781 | -41.2 | 3.1 | -46.3 | 2.8 | -25.9 | 6.6 | 36.6 | 3.9 | -5.5 | 0.1 |
| 180 | ZINC000058099587 | -41.2 | 2.8 | -46.8 | 2.8 | -18.8 | 3.4 | 29.8 | 2.4 | -5.3 | 0.2 |
| 181 | ZINC000178511392 | -41.2 | 2.7 | -40.2 | 2.5 | -33.5 | 4.1 | 37.7 | 2.6 | -5.2 | 0.1 |
| 182 | ZINC000005303591 | -41.1 | 2.3 | -44.2 | 2.5 | -20.2 | 3.9 | 28.9 | 2.4 | -5.7 | 0.1 |
| 183 | ZINC000010218408 | -41.1 | 3.9 | -47.3 | 2.8 | -19.2 | 5.7 | 30.7 | 3.4 | -5.3 | 0.2 |
| 184 | ZINC000003875380 | -41.1 | 2.1 | -46.6 | 1.9 | -15.5 | 2.0 | 25.9 | 1.7 | -4.9 | 0.1 |
| 185 | ZINC000072807915 | -41.1 | 2.4 | -50.6 | 2.3 | -7.2 | 3.6 | 22.8 | 3.3 | -6.1 | 0.2 |
| 186 | ZINC000018053795 | -41.0 | 2.9 | -47.5 | 2.3 | -22.6 | 3.8 | 34.8 | 2.6 | -5.8 | 0.1 |
| 187 | ZINC000014240188 | -41.0 | 2.7 | -45.7 | 2.8 | -17.2 | 4.2 | 27.7 | 3.3 | -5.8 | 0.1 |
| 188 | ZINC000077353582 | -41.0 | 3.2 | -48.6 | 2.8 | -15.4 | 3.1 | 28.6 | 2.1 | -5.5 | 0.1 |
| 189 | ZINC000239762559 | -40.9 | 2.7 | -48.8 | 2.5 | -21.2 | 4.1 | 34.8 | 2.9 | -5.7 | 0.2 |
| 190 | ZINC000067643674 | -40.9 | 2.4 | -44.8 | 2.5 | -25.8 | 4.2 | 35.1 | 2.3 | -5.4 | 0.1 |
| 191 | ZINC000089857747 | -40.8 | 3.9 | -50.7 | 4.5 | -7.6 | 5.0 | 23.4 | 5.5 | -5.9 | 0.3 |
| 192 | ZINC000154455008 | -40.7 | 3.4 | -44.9 | 2.8 | -15.2 | 6.0 | 25.2 | 2.8 | -5.8 | 0.2 |
| 193 | ZINC000306138347 | -40.7 | 2.2 | -49.7 | 2.0 | -17.2 | 3.2 | 32.1 | 2.5 | -5.9 | 0.2 |
| 194 | ZINC000585093122 | -40.6 | 2.4 | -45.2 | 2.6 | -16.8 | 4.4 | 27.0 | 2.8 | -5.6 | 0.2 |
| 195 | ZINC000008738218 | -40.6 | 2.9 | -41.0 | 2.9 | -40.6 | 5.5 | 46.7 | 3.2 | -5.6 | 0.1 |
| 196 | ZINC000091487017 | -40.6 | 2.4 | -49.3 | 2.3 | -11.4 | 3.0 | 26.4 | 2.4 | -6.2 | 0.1 |
| 197 | ZINC000007030648 | -40.6 | 3.6 | -43.3 | 2.9 | -20.2 | 7.1 | 27.9 | 3.2 | -5.0 | 0.3 |
| 198 | ZINC000192753414 | -40.5 | 3.1 | -46.9 | 2.7 | -32.1 | 4.6 | 44.2 | 2.3 | -5.7 | 0.1 |
| 199 | ZINC000096837115 | -40.5 | 5.9 | -45.6 | 4.0 | -27.4 | 7.7 | 38.3 | 5.2 | -5.7 | 0.3 |
| 200 | ZINC000077522487 | -40.5 | 2.5 | -48.6 | 2.2 | -28.5 | 5.2 | 42.4 | 4.0 | -5.8 | 0.2 |
| 201 | ZINC000018240833 | -40.5 | 2.9 | -47.5 | 2.8 | -37.8 | 5.3 | 51.0 | 3.3 | -6.2 | 0.2 |
| 202 | ZINC000084563579 | -40.4 | 3.1 | -45.4 | 4.0 | -20.3 | 15.5 | 31.0 | 12.0 | -5.8 | 0.2 |
| 203 | ZINC000095402766 | -40.4 | 4.5 | -44.9 | 2.5 | -16.8 | 7.1 | 27.2 | 3.3 | -5.9 | 0.1 |
| 204 | ZINC000330033487 | -40.4 | 3.0 | -50.1 | 2.6 | -15.9 | 4.3 | 31.8 | 2.5 | -6.1 | 0.1 |
| 205 | ZINC000136345745 | -40.4 | 2.6 | -51.8 | 2.4 | -19.5 | 4.9 | 37.1 | 4.1 | -6.2 | 0.2 |
| 206 | ZINC000006117573 | -40.3 | 2.4 | -42.2 | 2.4 | -28.0 | 2.9 | 35.4 | 2.1 | -5.5 | 0.1 |
| 207 | ZINC000040266307 | -40.3 | 3.0 | -44.5 | 2.5 | -22.2 | 4.2 | 32.5 | 2.9 | -6.1 | 0.2 |
| 208 | ZINC000095989182 | -40.2 | 2.5 | -43.2 | 2.2 | -18.7 | 3.6 | 27.1 | 2.2 | -5.4 | 0.1 |
| 209 | ZINC000000079035 | -40.2 | 3.4 | -47.2 | 2.3 | -19.1 | 5.5 | 31.8 | 3.2 | -5.7 | 0.1 |
| 210 | ZINC000016543876 | -40.2 | 3.2 | -46.0 | 2.3 | -16.9 | 3.7 | 28.5 | 2.2 | -5.9 | 0.1 |
| 211 | ZINC000071754931 | -40.2 | 2.5 | -40.8 | 2.3 | -12.2 | 3.3 | 18.3 | 1.9 | -5.5 | 0.1 |
| 212 | ZINC000019327176 | -40.2 | 3.2 | -40.2 | 3.0 | -26.8 | 5.7 | 31.9 | 3.4 | -5.2 | 0.2 |
| 213 | ZINC000177842371 | -40.1 | 2.4 | -45.3 | 2.0 | -26.9 | 3.8 | 37.3 | 2.8 | -5.3 | 0.1 |
| 214 | ZINC000048249797 | -40.1 | 2.6 | -50.8 | 2.7 | -10.3 | 5.2 | 27.1 | 3.5 | -6.2 | 0.1 |
| 215 | ZINC000012508838 | -40.1 | 2.7 | -49.0 | 2.3 | -24.8 | 4.2 | 39.9 | 2.9 | -6.2 | 0.1 |
| 216 | ZINC000097105546 | -40.1 | 2.5 | -44.7 | 2.6 | -22.4 | 3.8 | 32.6 | 3.4 | -5.6 | 0.1 |
| 217 | ZINC000013691850 | -40.1 | 2.3 | -49.9 | 2.5 | -15.3 | 3.3 | 30.8 | 2.1 | -5.7 | 0.1 |
| 218 | ZINC000002630701 | -40.1 | 2.4 | -49.0 | 2.0 | -6.8 | 4.2 | 21.4 | 3.4 | -5.8 | 0.1 |
| 219 | ZINC000096122792 | -40.1 | 2.5 | -48.6 | 2.6 | -9.2 | 2.7 | 23.4 | 2.2 | -5.7 | 0.1 |
| 220 | ZINC000091321614 | -39.9 | 2.8 | -49.4 | 2.4 | -18.7 | 3.8 | 34.3 | 3.4 | -6.1 | 0.2 |
| 221 | ZINC000075140034 | -39.9 | 2.4 | -41.8 | 2.4 | -19.3 | 2.9 | 27.0 | 2.0 | -5.7 | 0.2 |
| 222 | ZINC000019973951 | -39.9 | 3.8 | -40.3 | 3.0 | -29.4 | 6.4 | 34.9 | 3.3 | -5.1 | 0.2 |

| # | ZINC ID | ΔG<sub>bind</sub> | std | ΔE<sub>vdW</sub> | std | ΔE<sub>el</sub> | std | ΔG<sub>pol</sub> | std | ΔG<sub>np</sub> | std |
|---|---|---|---|---|---|---|---|---|---|---|---|
| 223 | ZINC000014741626 | -39.8 | 2.2 | -49.6 | 2.2 | -6.8 | 2.5 | 22.4 | 2.1 | -5.8 | 0.2 |
| 224 | ZINC000095951555 | -39.8 | 2.4 | -42.9 | 2.3 | -19.3 | 3.3 | 27.8 | 1.8 | -5.5 | 0.1 |
| 225 | ZINC000096069697 | -39.7 | 4.2 | -45.5 | 2.3 | -9.3 | 5.4 | 20.8 | 2.9 | -5.7 | 0.2 |
| 226 | ZINC000022067509 | -39.7 | 3.0 | -49.1 | 2.8 | -30.2 | 10.7 | 45.7 | 8.9 | -6.1 | 0.1 |
| 227 | ZINC000023764785 | -39.6 | 3.3 | -45.9 | 2.6 | -15.6 | 4.6 | 27.8 | 3.0 | -5.9 | 0.2 |
| 228 | ZINC000035671101 | -39.6 | 2.1 | -46.7 | 2.0 | -8.2 | 1.6 | 20.4 | 1.5 | -5.1 | 0.2 |
| 229 | ZINC000097050589 | -39.5 | 2.6 | -48.8 | 2.6 | -13.2 | 4.8 | 28.0 | 3.3 | -5.6 | 0.1 |
| 230 | ZINC000065591272 | -39.4 | 2.3 | -44.9 | 2.2 | -9.7 | 2.8 | 21.1 | 1.9 | -6.0 | 0.2 |
| 231 | ZINC000284798034 | -39.4 | 2.8 | -50.9 | 2.3 | -22.8 | 4.5 | 40.3 | 3.0 | -6.1 | 0.2 |
| 232 | ZINC000004535480 | -39.4 | 2.5 | -45.6 | 2.2 | -15.4 | 4.8 | 26.7 | 3.9 | -5.1 | 0.2 |
| 233 | ZINC000006782318 | -39.3 | 2.3 | -47.7 | 2.3 | -18.1 | 2.7 | 32.2 | 2.3 | -5.7 | 0.2 |
| 234 | ZINC000012407997 | -39.2 | 3.4 | -48.0 | 2.8 | -11.5 | 5.8 | 26.3 | 4.7 | -6.1 | 0.4 |
| 235 | ZINC000005026046 | -39.2 | 2.6 | -45.8 | 2.3 | -14.0 | 2.7 | 26.3 | 1.9 | -5.7 | 0.2 |
| 236 | ZINC000061067795 | -39.2 | 2.3 | -48.3 | 2.2 | -13.8 | 3.9 | 29.1 | 3.0 | -6.2 | 0.2 |
| 237 | ZINC000040386098 | -39.2 | 2.5 | -42.7 | 2.5 | -13.8 | 3.3 | 22.5 | 2.3 | -5.2 | 0.2 |
| 238 | ZINC000253407066 | -39.1 | 2.5 | -46.7 | 2.4 | -13.8 | 3.4 | 27.6 | 2.6 | -6.1 | 0.2 |
| 239 | ZINC000299793701 | -38.9 | 2.4 | -42.4 | 2.8 | -24.9 | 5.0 | 33.9 | 3.3 | -5.5 | 0.1 |
| 240 | ZINC000005348603 | -38.9 | 2.6 | -46.3 | 2.6 | -8.7 | 4.7 | 22.0 | 3.6 | -5.8 | 0.1 |
| 241 | ZINC000091943530 | -38.8 | 2.5 | -44.1 | 2.7 | -16.2 | 4.1 | 27.0 | 2.4 | -5.5 | 0.2 |
| 242 | ZINC000030875068 | -38.8 | 2.4 | -45.9 | 2.2 | -10.0 | 4.0 | 22.8 | 2.5 | -5.7 | 0.1 |
| 243 | ZINC000003313076 | -38.7 | 2.6 | -41.7 | 3.0 | -21.1 | 4.5 | 29.7 | 2.3 | -5.5 | 0.1 |
| 244 | ZINC000238831563 | -38.6 | 3.2 | -47.8 | 2.5 | -11.4 | 4.4 | 26.7 | 2.5 | -6.0 | 0.1 |
| 245 | ZINC000153353600 | -38.6 | 3.8 | -46.2 | 2.7 | -8.9 | 3.8 | 22.2 | 2.8 | -5.7 | 0.2 |
| 246 | ZINC000012239509 | -38.6 | 2.9 | -50.5 | 2.5 | -7.5 | 4.9 | 25.5 | 3.2 | -6.1 | 0.2 |
| 247 | ZINC000096080454 | -38.6 | 2.3 | -44.4 | 2.3 | -19.6 | 2.8 | 31.1 | 2.1 | -5.7 | 0.1 |
| 248 | ZINC000192929211 | -38.5 | 2.7 | -44.2 | 2.7 | -24.0 | 8.6 | 35.6 | 7.1 | -5.8 | 0.1 |
| 249 | ZINC000072896859 | -38.5 | 2.5 | -48.3 | 2.6 | -6.8 | 5.0 | 22.6 | 4.0 | -6.0 | 0.2 |
| 250 | ZINC000257270100 | -38.5 | 3.4 | -46.1 | 2.5 | -10.6 | 2.9 | 24.0 | 2.5 | -5.9 | 0.1 |
| 251 | ZINC000257257831 | -38.4 | 3.0 | -45.8 | 2.2 | -12.3 | 4.7 | 25.3 | 3.3 | -5.7 | 0.2 |
| 252 | ZINC000002837442 | -38.3 | 2.8 | -42.9 | 2.8 | -16.1 | 2.5 | 26.4 | 1.6 | -5.7 | 0.2 |
| 253 | ZINC000008776545 | -38.2 | 2.5 | -47.3 | 2.4 | -15.4 | 2.9 | 30.5 | 2.0 | -6.0 | 0.1 |
| 254 | ZINC000003327891 | -38.1 | 2.3 | -45.9 | 2.2 | -11.8 | 2.2 | 25.3 | 1.8 | -5.7 | 0.2 |
| 255 | ZINC000001810957 | -38.1 | 2.4 | -45.8 | 2.6 | -17.2 | 3.6 | 30.8 | 2.9 | -5.9 | 0.2 |
| 256 | ZINC000040385901 | -38.0 | 2.9 | -41.1 | 2.8 | -13.0 | 2.9 | 21.0 | 2.2 | -4.9 | 0.2 |
| 257 | ZINC000008609997 | -37.6 | 2.9 | -50.6 | 2.4 | -13.5 | 3.5 | 32.2 | 2.7 | -5.7 | 0.2 |
| 258 | ZINC000047209496 | -37.3 | 3.1 | -44.4 | 2.4 | -13.3 | 5.2 | 26.2 | 5.1 | -5.8 | 0.2 |
| 259 | ZINC000253473237 | -37.3 | 2.1 | -44.9 | 2.2 | -8.3 | 2.8 | 21.9 | 1.9 | -5.9 | 0.2 |
| 260 | ZINC000072277544 | -37.2 | 3.7 | -45.7 | 2.8 | -15.0 | 5.4 | 29.2 | 3.9 | -5.6 | 0.2 |
| 261 | ZINC000215185695 | -37.2 | 2.2 | -47.4 | 2.1 | -16.7 | 2.1 | 33.2 | 1.8 | -6.3 | 0.2 |
| 262 | ZINC000003852421 | -37.0 | 2.0 | -44.3 | 2.1 | -12.3 | 2.6 | 24.8 | 2.0 | -5.3 | 0.1 |
| 263 | ZINC000057511709 | -36.9 | 2.4 | -45.5 | 2.0 | -22.5 | 5.0 | 36.7 | 4.5 | -5.5 | 0.2 |
| 264 | ZINC000072234903 | -36.8 | 2.7 | -44.1 | 2.2 | -12.5 | 3.9 | 25.6 | 3.2 | -5.8 | 0.1 |
| 265 | ZINC000027755553 | -36.8 | 2.5 | -46.7 | 2.1 | -15.5 | 3.0 | 31.1 | 2.3 | -5.5 | 0.1 |
| 266 | ZINC000072432742 | -36.6 | 2.5 | -49.1 | 2.3 | 1.8 | 9.9 | 17.0 | 9.4 | -6.3 | 0.1 |
| 267 | ZINC000253433605 | -36.5 | 3.4 | -47.3 | 2.5 | -14.5 | 7.6 | 31.1 | 5.9 | -5.8 | 0.2 |
| 268 | ZINC000107713021 | -36.4 | 2.5 | -44.7 | 2.2 | -2.8 | 2.9 | 16.8 | 2.6 | -5.7 | 0.2 |

| # | ZINC ID | $\Delta G_{bind}$ | *std* | $\Delta E_{vdW}$ | *std* | $\Delta E_{el}$ | *std* | $\Delta G_{pol}$ | *std* | $\Delta G_{np}$ | *std* |
|---|---|---|---|---|---|---|---|---|---|---|---|
| 269 | ZINC000042025678 | -36.4 | 2.7 | -43.6 | 4.1 | -12.3 | 5.3 | 24.6 | 2.6 | -5.1 | 0.2 |
| 270 | ZINC000000326909 | -36.3 | 2.7 | -42.9 | 2.1 | -17.0 | 3.4 | 28.3 | 1.9 | -4.7 | 0.2 |
| 271 | ZINC000004807849 | -36.3 | 2.6 | -36.5 | 3.5 | -47.4 | 6.0 | 52.9 | 4.0 | -5.3 | 0.2 |
| 272 | ZINC000019796838 | -36.1 | 2.6 | -46.3 | 2.1 | -11.2 | 3.5 | 26.7 | 2.6 | -5.4 | 0.2 |
| 273 | ZINC000575617533 | -36.1 | 2.3 | -44.8 | 2.3 | -10.3 | 2.9 | 24.7 | 2.8 | -5.7 | 0.2 |
| 274 | ZINC000096038482 | -36.0 | 3.0 | -39.5 | 2.9 | -24.6 | 6.7 | 33.4 | 4.4 | -5.3 | 0.2 |
| 275 | ZINC000013278475 | -36.0 | 3.3 | -48.6 | 2.9 | -19.5 | 10.1 | 38.2 | 9.3 | -6.0 | 0.2 |
| 276 | ZINC000170016387 | -35.9 | 2.4 | -42.6 | 2.8 | -20.3 | 5.4 | 32.8 | 4.0 | -5.7 | 0.2 |
| 277 | ZINC000257311417 | -35.5 | 2.5 | -40.7 | 2.2 | -23.4 | 4.5 | 34.1 | 3.5 | -5.5 | 0.2 |
| 278 | ZINC000069740630 | -35.5 | 5.0 | -38.0 | 2.6 | -20.4 | 4.3 | 28.2 | 2.2 | -5.3 | 0.3 |
| 279 | ZINC000018177963 | -35.3 | 2.2 | -32.7 | 3.1 | -55.9 | 5.3 | 58.4 | 3.2 | -5.1 | 0.1 |
| 280 | ZINC000017888288 | -35.1 | 2.7 | -44.1 | 2.3 | -16.9 | 4.5 | 31.0 | 3.1 | -5.1 | 0.2 |
| 281 | ZINC000002390471 | -35.1 | 2.1 | -43.9 | 2.0 | -12.4 | 2.2 | 26.7 | 1.9 | -5.5 | 0.2 |
| 282 | ZINC000004722011 | -35.0 | 2.5 | -44.0 | 2.2 | -7.1 | 2.8 | 21.3 | 2.2 | -5.2 | 0.2 |
| 283 | ZINC000005088834 | -35.0 | 3.2 | -42.9 | 3.6 | -7.6 | 2.4 | 21.1 | 1.8 | -5.5 | 0.2 |
| 284 | ZINC000004732939 | -34.8 | 2.6 | -43.8 | 2.2 | -12.5 | 3.0 | 27.0 | 2.4 | -5.5 | 0.1 |
| 285 | ZINC000012122382 | -34.7 | 2.7 | -42.6 | 2.3 | -9.7 | 4.8 | 23.0 | 3.5 | -5.4 | 0.2 |
| 286 | ZINC000237740654 | -34.7 | 3.1 | -45.0 | 2.8 | -3.6 | 10.2 | 19.7 | 10.0 | -5.8 | 0.3 |
| 287 | ZINC000001381807 | -34.6 | 2.0 | -37.1 | 2.1 | -14.9 | 2.3 | 22.4 | 1.5 | -5.0 | 0.1 |
| 288 | ZINC000000201272 | -34.5 | 3.0 | -46.5 | 2.2 | -7.2 | 5.3 | 24.9 | 3.8 | -5.8 | 0.1 |
| 289 | ZINC000085879141 | -34.0 | 2.4 | -41.7 | 2.4 | -10.6 | 5.3 | 23.3 | 3.5 | -5.1 | 0.1 |
| 290 | ZINC000001620500 | -33.9 | 6.5 | -42.1 | 2.8 | -26.7 | 24.3 | 40.3 | 18.3 | -5.4 | 0.2 |
| 291 | ZINC000237955843 | -33.8 | 3.9 | -40.6 | 2.6 | -42.8 | 10.6 | 55.2 | 9.6 | -5.6 | 0.2 |
| 292 | ZINC000255187536 | -33.7 | 2.4 | -42.3 | 2.5 | -21.3 | 3.7 | 35.5 | 2.6 | -5.6 | 0.2 |
| 293 | ZINC000012557330 | -33.7 | 2.4 | -43.0 | 2.5 | -10.6 | 4.3 | 25.1 | 3.4 | -5.2 | 0.2 |
| 294 | ZINC000006918427 | -33.6 | 3.2 | -45.0 | 2.5 | -13.2 | 4.0 | 30.2 | 2.8 | -5.6 | 0.2 |
| 295 | ZINC000034407910 | -33.3 | 1.9 | -38.4 | 2.1 | -15.1 | 2.8 | 25.1 | 1.9 | -4.9 | 0.1 |
| 296 | ZINC000006556210 | -33.2 | 2.5 | -41.4 | 2.6 | -11.3 | 2.5 | 24.8 | 2.5 | -5.2 | 0.2 |
| 297 | ZINC000268943077 | -32.5 | 2.7 | -41.8 | 2.6 | 6.5 | 8.3 | 8.2 | 7.7 | -5.4 | 0.2 |
| 298 | ZINC000004806959 | -32.3 | 2.7 | -39.4 | 2.6 | -29.2 | 9.6 | 42.0 | 8.3 | -5.7 | 0.1 |
| 299 | ZINC000096198984 | -32.0 | 2.9 | -45.9 | 2.9 | -8.9 | 3.7 | 28.7 | 3.4 | -5.9 | 0.3 |
| 300 | ZINC000003947438 | -30.6 | 2.7 | -41.0 | 2.4 | -21.6 | 9.2 | 37.3 | 7.9 | -5.3 | 0.2 |
| 301 | ZINC000000353960 | -30.5 | 3.7 | -35.6 | 2.7 | -38.0 | 13.3 | 48.0 | 10.7 | -4.9 | 0.2 |
| 302 | ZINC000237699092 | -28.0 | 3.5 | -40.8 | 3.4 | -8.7 | 15.4 | 26.6 | 14.8 | -5.1 | 0.3 |
| 303 | ZINC000069895275 | -25.4 | 3.5 | -37.0 | 2.2 | -10.2 | 13.6 | 26.9 | 11.3 | -5.0 | 0.2 |
| 304 | ZINC000006576136 | -22.8 | 2.3 | -34.5 | 2.6 | -6.9 | 10.0 | 23.3 | 8.6 | -4.7 | 0.1 |
| 305 | ZINC000008848147 | -22.4 | 2.6 | -38.7 | 3.2 | -0.8 | 14.1 | 22.1 | 12.5 | -5.0 | 0.1 |

**Table S6.** Energy decomposition of the MM/GBSA results for the 305 compounds using the *GB-5* parameter set (for more details see the Methods section). All energy terms are given in kcal/mol with their standard deviation (*std*) and are sorted by the binding free energy ($\Delta G_{bind}$) followed by the molecular mechanics van der Waals and electrostatic interactions terms ($\Delta E_{vdW}$ and $\Delta E_{el}$) and the polar and non-polar contributions to the solvation free energy ($\Delta G_{pol}$ and $\Delta G_{np}$). Compounds marked in boldface were finally selected for experimental screening.

| # | ZINC ID | $\Delta G_{bind}$ | *std* | $\Delta E_{vdW}$ | *std* | $\Delta E_{el}$ | *std* | $\Delta G_{pol}$ | *std* | $\Delta G_{np}$ | *std* |
|---|---|---|---|---|---|---|---|---|---|---|---|
| 1 | ZINC000257317306 | -61.7 | 3.4 | -50.1 | 2.8 | -41.5 | 3.3 | 34.4 | 2.5 | -4.5 | 0.1 |
| **2** | **ZINC000035869910** | **-59.9** | **3.9** | **-54.6** | **2.6** | **-35.5** | **5.1** | **34.9** | **2.9** | **-4.7** | **0.1** |
| **3** | **ZINC000069778215** | **-56.4** | **3.8** | **-47.6** | **2.7** | **-41.7** | **4.2** | **37.2** | **2.8** | **-4.4** | **0.1** |
| **4** | **ZINC000023642159** | **-56.4** | **3.4** | **-47.6** | **2.3** | **-27.5** | **4.0** | **22.8** | **2.5** | **-4.0** | **0.1** |
| 5 | ZINC000096058349 | -55.7 | 3.2 | -54.1 | 2.4 | -18.0 | 2.3 | 20.9 | 2.2 | -4.5 | 0.1 |
| 6 | ZINC000096158052 | -55.4 | 3.3 | -49.9 | 2.5 | -20.8 | 3.8 | 19.6 | 3.0 | -4.3 | 0.1 |
| **7** | **ZINC000091270543** | **-53.9** | **3.0** | **-53.6** | **3.0** | **-23.0** | **3.8** | **27.5** | **2.3** | **-4.7** | **0.1** |
| 8 | ZINC000257211885 | -53.7 | 5.5 | -50.3 | 3.9 | -20.9 | 3.8 | 21.5 | 3.1 | -3.9 | 0.1 |
| 9 | ZINC000278659999 | -52.4 | 3.4 | -47.8 | 2.4 | -23.5 | 3.5 | 23.1 | 2.4 | -4.2 | 0.1 |
| **10** | **ZINC000008595944** | **-52.4** | **3.2** | **-53.1** | **2.3** | **-13.8** | **4.1** | **18.6** | **3.6** | **-4.1** | **0.1** |
| 11 | ZINC000096251709 | -52.2 | 3.5 | -48.0 | 2.1 | -19.0 | 3.2 | 18.8 | 2.2 | -4.0 | 0.1 |
| 12 | ZINC000091486946 | -52.0 | 2.8 | -52.4 | 2.6 | -21.0 | 2.8 | 26.0 | 2.7 | -4.6 | 0.1 |
| 13 | ZINC000409428381 | -51.9 | 3.8 | -51.5 | 2.4 | -12.1 | 4.9 | 16.4 | 3.1 | -4.6 | 0.1 |
| 14 | ZINC000065395709 | -51.5 | 3.0 | -51.6 | 2.4 | -22.5 | 3.1 | 27.1 | 2.0 | -4.6 | 0.1 |
| **15** | **ZINC000003463362** | **-51.5** | **3.4** | **-50.0** | **2.8** | **-30.9** | **4.2** | **33.8** | **2.5** | **-4.4** | **0.1** |
| 16 | ZINC000075530913 | -51.0 | 2.5 | -49.2 | 2.3 | -20.1 | 2.3 | 22.5 | 1.8 | -4.3 | 0.1 |
| **17** | **ZINC000091486133** | **-50.6** | **3.1** | **-52.7** | **2.9** | **-25.7** | **3.2** | **32.4** | **2.2** | **-4.6** | **0.1** |
| 18 | ZINC000257218603 | -50.6 | 3.4 | -50.2 | 2.5 | -24.7 | 5.1 | 28.8 | 3.5 | -4.5 | 0.1 |
| **19** | **ZINC000008913866** | **-50.6** | **3.5** | **-50.7** | **2.5** | **-29.7** | **6.1** | **34.2** | **4.4** | **-4.4** | **0.1** |
| 20 | ZINC000003641308 | -50.2 | 6.8 | -45.0 | 2.6 | -25.1 | 7.2 | 23.9 | 3.5 | -4.0 | 0.1 |
| 21 | ZINC000057731402 | -50.0 | 3.3 | -51.2 | 2.6 | -25.6 | 3.7 | 31.1 | 2.1 | -4.4 | 0.1 |
| 22 | ZINC000096251711 | -49.9 | 4.0 | -50.9 | 3.0 | -15.0 | 3.5 | 20.4 | 2.4 | -4.3 | 0.1 |
| 23 | ZINC000092022499 | -49.8 | 2.6 | -53.4 | 2.6 | -23.3 | 2.6 | 31.4 | 2.0 | -4.6 | 0.1 |
| **24** | **ZINC000022191233** | **-49.4** | **3.0** | **-53.6** | **2.9** | **-31.4** | **3.7** | **40.2** | **2.8** | **-4.6** | **0.1** |
| 25 | ZINC000170800517 | -49.3 | 2.8 | -49.4 | 2.4 | -18.6 | 2.4 | 23.3 | 2.1 | -4.5 | 0.1 |
| 26 | ZINC000007030710 | -48.9 | 3.7 | -47.3 | 2.7 | -25.7 | 4.3 | 28.0 | 3.1 | -3.9 | 0.1 |
| 27 | ZINC000091270544 | -48.9 | 3.0 | -52.4 | 2.7 | -22.2 | 3.8 | 30.3 | 2.6 | -4.5 | 0.1 |
| **28** | **ZINC000084578820** | **-48.8** | **3.6** | **-47.8** | **2.6** | **-35.8** | **7.0** | **39.0** | **3.7** | **-4.1** | **0.1** |
| 29 | ZINC000072290753 | -48.8 | 4.0 | -52.3 | 2.4 | -25.4 | 4.1 | 33.2 | 3.3 | -4.2 | 0.1 |
| **30** | **ZINC000015078577** | **-48.7** | **3.4** | **-49.4** | **2.8** | **-29.5** | **4.6** | **34.7** | **3.2** | **-4.4** | **0.1** |
| **31** | **ZINC000010256259** | **-48.6** | **3.3** | **-52.7** | **2.7** | **-42.1** | **3.7** | **50.7** | **3.1** | **-4.5** | **0.1** |
| 32 | ZINC000077503523 | -48.4 | 3.3 | -45.1 | 2.9 | -34.1 | 6.6 | 34.7 | 4.8 | -4.0 | 0.1 |
| 33 | ZINC000019328346 | -48.3 | 8.8 | -46.7 | 4.6 | -22.0 | 4.6 | 24.5 | 3.0 | -4.1 | 0.1 |
| 34 | ZINC000003875380 | -48.2 | 2.6 | -46.6 | 1.9 | -16.5 | 2.0 | 18.2 | 2.2 | -3.4 | 0.1 |
| **35** | **ZINC000095415912** | **-48.2** | **3.5** | **-53.2** | **2.7** | **-28.4** | **4.2** | **37.7** | **2.7** | **-4.4** | **0.1** |
| 36 | ZINC000092065700 | -48.1 | 2.9 | -52.6 | 2.5 | -19.7 | 2.9 | 28.5 | 2.3 | -4.3 | 0.1 |
| 37 | ZINC000095950700 | -48.0 | 2.7 | -48.8 | 2.7 | -19.6 | 2.1 | 24.7 | 2.2 | -4.2 | 0.1 |
| 38 | ZINC000257222272 | -47.9 | 3.1 | -51.4 | 2.2 | -7.4 | 2.0 | 15.2 | 2.1 | -4.4 | 0.1 |

| # | ZINC ID | Δ$G_{bind}$ | *std* | Δ$E_{vdW}$ | *std* | Δ$E_{el}$ | *std* | Δ$G_{pol}$ | *std* | Δ$G_{np}$ | *std* |
|---|---|---|---|---|---|---|---|---|---|---|---|
| 39 | ZINC000096238948 | -47.8 | 4.3 | -53.8 | 2.7 | -14.2 | 5.3 | 24.6 | 3.2 | -4.3 | 0.2 |
| **40** | **ZINC000095410774** | **-47.5** | **2.9** | **-50.5** | **2.4** | **-24.2** | **3.1** | **31.4** | **2.6** | **-4.2** | **0.1** |
| 41 | ZINC000100737122 | -47.4 | 3.0 | -52.3 | 2.5 | -22.6 | 3.1 | 31.8 | 2.9 | -4.3 | 0.1 |
| 42 | ZINC000257299555 | -47.4 | 2.9 | -46.7 | 3.1 | -38.1 | 4.3 | 41.7 | 3.4 | -4.3 | 0.1 |
| 43 | ZINC000002465084 | -47.0 | 4.2 | -49.8 | 2.5 | -23.0 | 3.3 | 30.1 | 2.9 | -4.2 | 0.1 |
| **44** | **ZINC000004998502** | **-47.0** | **3.4** | **-53.5** | **3.3** | **-39.4** | **4.2** | **50.3** | **3.0** | **-4.4** | **0.1** |
| **45** | **ZINC000000333210** | **-46.9** | **3.0** | **-50.6** | **3.0** | **-32.6** | **3.7** | **40.5** | **2.5** | **-4.3** | **0.1** |
| 46 | ZINC000091768990 | -46.8 | 2.7 | -52.4 | 2.8 | -14.9 | 2.6 | 24.8 | 2.2 | -4.4 | 0.1 |
| 47 | ZINC000229623928 | -46.8 | 2.9 | -50.6 | 2.5 | -33.6 | 3.7 | 41.9 | 3.2 | -4.4 | 0.1 |
| **48** | **ZINC000057563200** | **-46.8** | **3.4** | **-49.9** | **2.7** | **-17.6** | **4.6** | **24.9** | **2.4** | **-4.1** | **0.1** |
| **49** | **ZINC000002216953** | **-46.7** | **2.8** | **-48.7** | **2.4** | **-24.8** | **3.2** | **31.0** | **2.7** | **-4.3** | **0.1** |
| 50 | ZINC000034895992 | -46.7 | 3.5 | -49.7 | 2.7 | -29.8 | 3.4 | 37.1 | 3.0 | -4.3 | 0.1 |
| 51 | ZINC000334160460 | -46.7 | 2.9 | -49.1 | 2.5 | -22.9 | 2.9 | 29.6 | 2.1 | -4.2 | 0.1 |
| 52 | ZINC000097201269 | -46.5 | 3.7 | -49.4 | 2.6 | -17.3 | 4.2 | 24.7 | 3.0 | -4.4 | 0.2 |
| 53 | ZINC000091304527 | -46.3 | 3.2 | -50.9 | 2.5 | -14.3 | 4.5 | 23.2 | 3.0 | -4.3 | 0.2 |
| 54 | ZINC000009338249 | -46.3 | 3.9 | -47.0 | 2.5 | -27.3 | 4.7 | 31.9 | 2.9 | -3.9 | 0.1 |
| 55 | ZINC000071746202 | -46.2 | 3.7 | -47.6 | 2.6 | -14.6 | 3.6 | 20.3 | 2.9 | -4.2 | 0.1 |
| 56 | ZINC000194368147 | -46.1 | 3.2 | -48.3 | 2.8 | -31.3 | 3.5 | 37.9 | 2.4 | -4.4 | 0.1 |
| 57 | ZINC000097413355 | -45.9 | 2.4 | -46.7 | 2.6 | -27.2 | 3.2 | 31.9 | 2.6 | -3.9 | 0.1 |
| **58** | **ZINC000084598177** | **-45.9** | **2.8** | **-49.0** | **2.5** | **-29.0** | **3.2** | **36.1** | **2.6** | **-4.0** | **0.1** |
| 59 | ZINC000012323932 | -45.8 | 4.1 | -42.7 | 3.2 | -38.4 | 7.2 | 39.1 | 3.4 | -3.9 | 0.1 |
| 60 | ZINC000096199614 | -45.8 | 3.2 | -51.3 | 2.5 | -28.0 | 3.7 | 37.5 | 2.3 | -3.9 | 0.1 |
| 61 | ZINC000225746732 | -45.7 | 2.4 | -47.5 | 2.3 | -21.3 | 2.3 | 26.9 | 2.2 | -3.8 | 0.1 |
| 62 | ZINC000257310083 | -45.7 | 3.3 | -47.2 | 2.6 | -17.0 | 2.4 | 22.2 | 2.0 | -3.7 | 0.1 |
| **63** | **ZINC000025032259** | **-45.7** | **3.2** | **-54.4** | **2.7** | **-22.2** | **3.2** | **35.4** | **2.6** | **-4.5** | **0.1** |
| **64** | **ZINC000069562405** | **-45.0** | **2.8** | **-47.4** | **2.6** | **-27.1** | **2.9** | **33.5** | **2.4** | **-4.1** | **0.1** |
| 65 | ZINC000306141880 | -45.0 | 2.8 | -46.5 | 2.8 | -29.4 | 4.2 | 35.0 | 2.7 | -4.2 | 0.1 |
| 66 | ZINC000096069207 | -45.0 | 3.5 | -52.2 | 2.8 | -19.5 | 2.9 | 31.1 | 2.5 | -4.5 | 0.1 |
| **67** | **ZINC000051467674** | **-44.9** | **3.0** | **-46.6** | **2.7** | **-40.1** | **3.8** | **46.2** | **2.6** | **-4.4** | **0.1** |
| 68 | ZINC000575618196 | -44.9 | 3.2 | -54.5 | 2.7 | -27.3 | 3.9 | 41.4 | 2.7 | -4.5 | 0.1 |
| 69 | ZINC000012142783 | -44.9 | 4.2 | -51.0 | 2.5 | -22.0 | 5.0 | 32.4 | 5.1 | -4.3 | 0.1 |
| 70 | ZINC000257206611 | -44.6 | 3.1 | -55.3 | 3.1 | -11.5 | 5.7 | 26.6 | 3.9 | -4.4 | 0.1 |
| 71 | ZINC000013692677 | -44.5 | 3.9 | -51.9 | 3.1 | -30.2 | 4.7 | 42.1 | 3.3 | -4.6 | 0.1 |
| 72 | ZINC000040473994 | -44.5 | 2.8 | -49.6 | 2.8 | -26.5 | 4.0 | 35.6 | 2.4 | -4.1 | 0.1 |
| 73 | ZINC000238121810 | -44.4 | 4.5 | -37.2 | 2.9 | -110.9 | 10.4 | 107.9 | 8.3 | -4.1 | 0.1 |
| 74 | ZINC000257192562 | -44.4 | 2.3 | -45.9 | 2.2 | -17.6 | 2.3 | 23.3 | 2.0 | -4.1 | 0.1 |
| 75 | ZINC000065248686 | -44.2 | 2.7 | -45.7 | 2.6 | -14.8 | 2.7 | 20.4 | 1.9 | -4.1 | 0.1 |
| 76 | ZINC000092078469 | -43.7 | 4.6 | -45.0 | 3.2 | -24.1 | 3.6 | 29.1 | 3.5 | -3.8 | 0.2 |
| **77** | **ZINC000079491038** | **-43.5** | **3.3** | **-51.3** | **2.7** | **-28.2** | **5.1** | **40.5** | **4.0** | **-4.5** | **0.1** |
| 78 | ZINC000089827813 | -43.5 | 3.4 | -46.6 | 3.2 | -29.5 | 4.7 | 36.6 | 2.9 | -4.0 | 0.1 |
| 79 | ZINC000225489282 | -43.4 | 2.9 | -49.8 | 2.4 | -17.0 | 4.8 | 27.6 | 3.9 | -4.2 | 0.1 |
| 80 | ZINC000330033487 | -43.4 | 4.3 | -50.1 | 2.6 | -15.4 | 4.4 | 26.4 | 2.8 | -4.3 | 0.1 |
| 81 | ZINC000195547314 | -43.2 | 3.1 | -50.9 | 2.8 | -14.0 | 3.2 | 26.1 | 2.1 | -4.3 | 0.1 |
| 82 | ZINC000328587242 | -43.1 | 2.5 | -45.2 | 2.7 | -24.8 | 2.6 | 31.1 | 2.0 | -4.2 | 0.1 |
| 83 | ZINC000000489138 | -43.0 | 3.1 | -50.3 | 2.3 | -20.1 | 2.9 | 31.6 | 2.0 | -4.2 | 0.1 |
| 84 | ZINC000092760184 | -42.9 | 2.8 | -47.5 | 2.4 | -20.6 | 3.3 | 29.5 | 2.6 | -4.3 | 0.1 |

| # | ZINC ID | Δ$G_{bind}$ | *std* | Δ$E_{vdW}$ | *std* | Δ$E_{el}$ | *std* | Δ$G_{pol}$ | *std* | Δ$G_{np}$ | *std* |
|---|---|---|---|---|---|---|---|---|---|---|---|
| 85 | ZINC000257256038 | -42.9 | 2.7 | -45.9 | 2.4 | -25.3 | 3.1 | 32.6 | 1.9 | -4.3 | 0.1 |
| 86 | ZINC000100193536 | -42.9 | 2.8 | -48.7 | 2.9 | -15.8 | 2.9 | 25.5 | 2.2 | -3.8 | 0.1 |
| **87** | **ZINC000071915224** | **-42.8** | **2.7** | **-49.3** | **2.3** | **-26.5** | **3.5** | **37.1** | **2.8** | **-4.1** | **0.1** |
| 88 | ZINC000230113696 | -42.7 | 3.0 | -52.9 | 2.8 | -23.6 | 4.3 | 38.1 | 3.8 | -4.3 | 0.1 |
| 89 | ZINC000072300733 | -42.7 | 4.6 | -51.3 | 2.7 | -13.5 | 4.4 | 26.4 | 3.2 | -4.3 | 0.1 |
| 90 | ZINC000071282106 | -42.6 | 4.0 | -47.7 | 2.6 | -16.3 | 4.9 | 25.7 | 2.4 | -4.3 | 0.1 |
| **91** | **ZINC000005360885** | **-42.6** | **3.7** | **-48.1** | **2.7** | **-36.1** | **4.9** | **45.4** | **2.3** | **-3.9** | **0.1** |
| 92 | ZINC000100193430 | -42.6 | 2.3 | -47.9 | 2.4 | -14.3 | 2.6 | 23.3 | 1.9 | -3.7 | 0.1 |
| 93 | ZINC000012550491 | -42.6 | 2.9 | -46.4 | 2.6 | -24.3 | 3.5 | 32.1 | 2.5 | -4.0 | 0.1 |
| 94 | ZINC000072096437 | -42.6 | 2.8 | -50.9 | 2.4 | -18.7 | 4.2 | 31.6 | 2.7 | -4.6 | 0.1 |
| **95** | **ZINC000244816835** | **-42.6** | **3.1** | **-43.8** | **3.0** | **-34.4** | **3.6** | **39.7** | **2.1** | **-4.1** | **0.1** |
| 96 | ZINC000065463577 | -42.5 | 2.8 | -46.6 | 2.7 | -31.9 | 5.2 | 40.2 | 4.8 | -4.1 | 0.1 |
| 97 | ZINC000002750765 | -42.5 | 2.8 | -45.0 | 2.4 | -24.2 | 2.9 | 30.9 | 2.7 | -4.2 | 0.1 |
| 98 | ZINC000075118384 | -42.3 | 3.9 | -46.9 | 2.9 | -33.1 | 7.3 | 41.8 | 4.1 | -4.1 | 0.1 |
| **99** | **ZINC000223692657** | **-42.2** | **2.9** | **-50.8** | **2.6** | **-28.2** | **3.9** | **40.9** | **2.7** | **-4.2** | **0.1** |
| 100 | ZINC000069846908 | -42.2 | 3.8 | -50.0 | 3.0 | -33.3 | 7.7 | 45.3 | 5.0 | -4.2 | 0.1 |
| **101** | **ZINC000069737923** | **-42.1** | **3.1** | **-46.3** | **2.3** | **-30.1** | **4.0** | **38.6** | **2.8** | **-4.3** | **0.1** |
| **102** | **ZINC000018148904** | **-42.1** | **2.9** | **-50.1** | **2.6** | **-32.4** | **3.5** | **44.5** | **2.6** | **-4.1** | **0.1** |
| 103 | ZINC000013691850 | -42.0 | 2.6 | -49.9 | 2.5 | -15.3 | 3.3 | 27.2 | 2.0 | -3.9 | 0.1 |
| 104 | ZINC000095393415 | -41.9 | 2.9 | -54.8 | 2.5 | -14.4 | 3.2 | 31.2 | 2.8 | -4.0 | 0.1 |
| 105 | ZINC000011781217 | -41.8 | 3.2 | -52.0 | 2.2 | -13.7 | 2.4 | 28.3 | 2.1 | -4.3 | 0.1 |
| 106 | ZINC000091664087 | -41.7 | 3.2 | -54.4 | 2.3 | -8.4 | 3.4 | 25.6 | 2.4 | -4.5 | 0.1 |
| 107 | ZINC000003292603 | -41.7 | 3.5 | -47.2 | 2.4 | -19.2 | 3.7 | 28.9 | 3.0 | -4.2 | 0.1 |
| 108 | ZINC000013691849 | -41.6 | 4.0 | -44.2 | 3.0 | -33.9 | 4.8 | 40.5 | 2.7 | -3.9 | 0.1 |
| 109 | ZINC000003438521 | -41.6 | 2.7 | -45.4 | 2.3 | -24.1 | 4.5 | 31.8 | 2.9 | -3.9 | 0.1 |
| 110 | ZINC000096024249 | -41.5 | 3.4 | -47.4 | 2.4 | -17.8 | 5.0 | 27.4 | 3.4 | -3.7 | 0.1 |
| 111 | ZINC000022067509 | -41.5 | 4.6 | -49.1 | 2.8 | -56.9 | 11.1 | 68.7 | 8.5 | -4.2 | 0.1 |
| 112 | ZINC000216315534 | -41.4 | 2.9 | -41.9 | 2.5 | -28.6 | 4.7 | 32.9 | 4.0 | -3.9 | 0.1 |
| 113 | ZINC000238886413 | -41.3 | 4.0 | -49.4 | 2.7 | -11.3 | 3.3 | 23.5 | 2.3 | -4.1 | 0.1 |
| 114 | ZINC000096003964 | -41.3 | 4.1 | -49.1 | 2.6 | -26.9 | 4.8 | 38.8 | 2.6 | -4.1 | 0.1 |
| 115 | ZINC000040060202 | -41.3 | 3.5 | -46.1 | 2.8 | -17.7 | 3.4 | 26.6 | 2.0 | -4.1 | 0.1 |
| 116 | ZINC000095503625 | -41.1 | 3.5 | -50.6 | 2.6 | -24.3 | 4.9 | 38.3 | 3.3 | -4.6 | 0.1 |
| 117 | ZINC000092021781 | -41.1 | 3.4 | -49.3 | 2.9 | -21.9 | 5.0 | 34.4 | 2.9 | -4.2 | 0.1 |
| **118** | **ZINC000043550595** | **-41.1** | **2.9** | **-48.3** | **2.5** | **-29.1** | **3.4** | **40.5** | **2.0** | **-4.3** | **0.1** |
| 119 | ZINC000107713021 | -41.0 | 3.0 | -44.7 | 2.2 | -3.4 | 2.9 | 11.1 | 3.1 | -4.0 | 0.1 |
| 120 | ZINC000257270100 | -41.0 | 4.5 | -46.1 | 2.5 | -11.5 | 2.9 | 20.6 | 3.4 | -4.1 | 0.1 |
| 121 | ZINC000005426253 | -40.9 | 2.9 | -49.8 | 2.4 | -12.6 | 3.2 | 26.1 | 3.7 | -4.5 | 0.1 |
| 122 | ZINC000044874685 | -40.9 | 3.1 | -44.2 | 2.8 | -24.3 | 4.6 | 31.6 | 3.6 | -4.0 | 0.1 |
| 123 | ZINC000004722089 | -40.7 | 2.3 | -48.1 | 2.5 | -14.7 | 2.7 | 25.8 | 1.8 | -3.7 | 0.1 |
| 124 | ZINC000014741626 | -40.6 | 2.6 | -49.6 | 2.2 | -7.1 | 2.5 | 20.1 | 2.5 | -4.0 | 0.1 |
| 125 | ZINC000069595091 | -40.6 | 3.4 | -45.8 | 2.8 | -22.3 | 3.9 | 31.6 | 3.0 | -4.2 | 0.1 |
| 126 | ZINC000003327891 | -40.5 | 2.6 | -45.9 | 2.2 | -11.5 | 2.2 | 20.8 | 2.0 | -4.0 | 0.1 |
| 127 | ZINC000100119126 | -40.5 | 3.4 | -47.3 | 2.8 | -19.9 | 4.3 | 30.9 | 3.4 | -4.2 | 0.1 |
| 128 | ZINC000071754931 | -40.4 | 2.8 | -40.8 | 2.3 | -12.7 | 3.3 | 16.9 | 2.0 | -3.8 | 0.1 |
| 129 | ZINC000069349055 | -40.4 | 3.5 | -48.3 | 3.0 | -24.4 | 8.4 | 36.7 | 5.7 | -4.3 | 0.1 |
| 130 | ZINC000096010936 | -40.4 | 3.1 | -41.4 | 2.8 | -27.6 | 3.2 | 32.7 | 2.3 | -4.1 | 0.1 |

| # | ZINC ID | $\Delta G_{bind}$ | *std* | $\Delta E_{vdW}$ | *std* | $\Delta E_{el}$ | *std* | $\Delta G_{pol}$ | *std* | $\Delta G_{np}$ | *std* |
|---|---|---|---|---|---|---|---|---|---|---|---|
| 131 | ZINC000014791001 | -40.3 | 2.9 | -47.0 | 2.3 | -21.6 | 3.5 | 32.5 | 3.1 | -4.3 | 0.1 |
| 132 | ZINC000100191495 | -40.3 | 2.8 | -46.6 | 2.6 | -14.5 | 2.6 | 24.6 | 2.0 | -3.7 | 0.1 |
| **133** | **ZINC000004511530** | **-40.3** | **2.6** | **-49.2** | **2.1** | **-22.4** | **3.0** | **35.7** | **2.3** | **-4.3** | **0.1** |
| 134 | ZINC000002646979 | -40.2 | 2.7 | -51.5 | 2.3 | -22.4 | 3.2 | 38.0 | 2.7 | -4.3 | 0.1 |
| 135 | ZINC000071497775 | -40.2 | 3.3 | -51.3 | 2.3 | -25.4 | 6.6 | 41.0 | 5.0 | -4.5 | 0.1 |
| 136 | ZINC000022549345 | -40.2 | 2.2 | -53.9 | 2.1 | -16.6 | 3.8 | 34.3 | 3.8 | -4.1 | 0.1 |
| 137 | ZINC000585280336 | -40.1 | 4.5 | -51.7 | 2.3 | -29.6 | 5.8 | 45.5 | 3.3 | -4.3 | 0.1 |
| 138 | ZINC000005026046 | -40.1 | 3.3 | -45.8 | 2.3 | -14.0 | 2.7 | 23.6 | 2.1 | -3.9 | 0.1 |
| 139 | ZINC000035398834 | -40.1 | 2.4 | -51.5 | 2.3 | -8.9 | 2.5 | 24.5 | 2.1 | -4.1 | 0.1 |
| 140 | ZINC000072896859 | -40.0 | 3.2 | -48.3 | 2.6 | -6.4 | 5.0 | 18.8 | 5.1 | -4.2 | 0.1 |
| 141 | ZINC000072290711 | -39.9 | 2.6 | -46.7 | 2.9 | -17.3 | 3.3 | 27.8 | 2.4 | -3.7 | 0.1 |
| 142 | ZINC000064871452 | -39.9 | 3.4 | -49.9 | 2.5 | -26.3 | 7.2 | 40.5 | 5.2 | -4.3 | 0.1 |
| 143 | ZINC000050584912 | -39.8 | 3.3 | -50.3 | 2.8 | -6.7 | 3.9 | 21.3 | 3.7 | -4.2 | 0.2 |
| 144 | ZINC000067460397 | -39.8 | 2.7 | -50.1 | 2.2 | -34.7 | 3.6 | 49.0 | 2.8 | -4.0 | 0.1 |
| 145 | ZINC000040386093 | -39.8 | 3.1 | -45.0 | 2.7 | -14.5 | 3.0 | 23.6 | 2.9 | -3.8 | 0.1 |
| 146 | ZINC000067643674 | -39.7 | 2.7 | -44.8 | 2.5 | -25.4 | 4.2 | 34.2 | 2.4 | -3.7 | 0.1 |
| 147 | ZINC000065167646 | -39.6 | 3.6 | -47.8 | 2.7 | -22.4 | 4.0 | 34.7 | 3.9 | -4.1 | 0.1 |
| 148 | ZINC000008738218 | -39.6 | 3.3 | -41.0 | 2.9 | -41.3 | 5.6 | 46.7 | 3.1 | -3.9 | 0.1 |
| 149 | ZINC000079001413 | -39.4 | 2.6 | -52.9 | 2.5 | -9.0 | 2.5 | 26.8 | 2.1 | -4.3 | 0.1 |
| **150** | **ZINC000044919578** | **-39.4** | **2.5** | **-43.0** | **2.1** | **-29.3** | **3.4** | **36.7** | **2.1** | **-3.8** | **0.1** |
| 151 | ZINC000013973188 | -39.3 | 2.7 | -47.2 | 2.5 | -29.0 | 3.6 | 41.1 | 2.8 | -4.2 | 0.1 |
| 152 | ZINC000007030648 | -39.3 | 4.1 | -43.3 | 2.9 | -21.2 | 7.0 | 28.7 | 3.2 | -3.5 | 0.2 |
| 153 | ZINC000135810445 | -39.1 | 3.3 | -44.5 | 2.6 | -33.4 | 5.4 | 42.9 | 3.5 | -4.1 | 0.1 |
| 154 | ZINC000002922856 | -39.1 | 3.2 | -52.4 | 2.7 | -15.2 | 3.6 | 32.8 | 2.9 | -4.3 | 0.1 |
| 155 | ZINC000096080454 | -39.1 | 2.8 | -44.4 | 2.3 | -19.0 | 2.8 | 28.2 | 2.4 | -3.9 | 0.1 |
| **156** | **ZINC000240454699** | **-39.0** | **2.7** | **-42.5** | **2.5** | **-25.1** | **4.6** | **32.7** | **3.4** | **-4.0** | **0.1** |
| 157 | ZINC000095402766 | -39.0 | 4.6 | -44.9 | 2.5 | -17.6 | 7.1 | 27.6 | 3.4 | -4.1 | 0.1 |
| 158 | ZINC000008316344 | -39.0 | 2.5 | -51.0 | 2.4 | -5.6 | 2.6 | 21.7 | 2.4 | -4.1 | 0.1 |
| 159 | ZINC000084686490 | -38.9 | 2.6 | -46.1 | 2.4 | -33.4 | 3.0 | 44.8 | 2.2 | -4.2 | 0.1 |
| 160 | ZINC000253433259 | -38.9 | 2.7 | -49.1 | 2.2 | -19.7 | 2.8 | 33.9 | 2.6 | -4.0 | 0.1 |
| 161 | ZINC000584887110 | -38.9 | 2.5 | -46.9 | 2.2 | -13.7 | 2.9 | 25.7 | 2.4 | -4.0 | 0.1 |
| 162 | ZINC000012888207 | -38.8 | 2.3 | -40.8 | 2.2 | -27.7 | 3.4 | 33.2 | 2.3 | -3.5 | 0.1 |
| 163 | ZINC000014240188 | -38.8 | 2.8 | -45.7 | 2.8 | -19.3 | 4.2 | 30.2 | 3.5 | -4.0 | 0.1 |
| 164 | ZINC000069775761 | -38.7 | 4.1 | -48.1 | 2.6 | -21.4 | 4.1 | 34.9 | 2.2 | -4.1 | 0.1 |
| 165 | ZINC000096069697 | -38.7 | 4.5 | -45.5 | 2.3 | -9.5 | 5.4 | 20.3 | 3.0 | -3.9 | 0.2 |
| 166 | ZINC000154455008 | -38.7 | 4.0 | -44.9 | 2.8 | -15.4 | 6.2 | 25.7 | 2.7 | -4.0 | 0.1 |
| 167 | ZINC000010218408 | -38.6 | 4.3 | -47.3 | 2.8 | -19.0 | 5.6 | 31.4 | 3.8 | -3.7 | 0.2 |
| 168 | ZINC000050859891 | -38.6 | 3.6 | -48.4 | 2.6 | -28.7 | 4.7 | 42.6 | 3.9 | -4.1 | 0.1 |
| 169 | ZINC000072290626 | -38.6 | 2.9 | -48.7 | 2.2 | -18.8 | 5.2 | 32.8 | 4.7 | -4.0 | 0.1 |
| 170 | ZINC000069492530 | -38.5 | 3.0 | -47.5 | 2.6 | -19.0 | 3.3 | 31.8 | 2.1 | -3.8 | 0.1 |
| 171 | ZINC000005348603 | -38.5 | 3.1 | -46.3 | 2.6 | -9.9 | 4.8 | 21.7 | 3.3 | -4.1 | 0.1 |
| 172 | ZINC000052955795 | -38.5 | 3.6 | -46.7 | 2.6 | -27.4 | 4.9 | 39.8 | 2.6 | -4.2 | 0.1 |
| 173 | ZINC000171342968 | -38.5 | 3.4 | -48.4 | 2.7 | -40.1 | 3.9 | 54.5 | 3.1 | -4.4 | 0.1 |
| 174 | ZINC000006887927 | -38.2 | 2.7 | -48.4 | 2.6 | -23.5 | 3.7 | 38.1 | 2.7 | -4.4 | 0.1 |
| 175 | ZINC000013278475 | -38.2 | 4.3 | -48.6 | 2.9 | -43.7 | 10.1 | 58.3 | 8.8 | -4.2 | 0.1 |
| 176 | ZINC000018053795 | -38.2 | 3.6 | -47.5 | 2.3 | -23.2 | 3.7 | 36.5 | 3.0 | -4.0 | 0.1 |

| # | ZINC ID | $\Delta G_{bind}$ | *std* | $\Delta E_{vdW}$ | *std* | $\Delta E_{el}$ | *std* | $\Delta G_{pol}$ | *std* | $\Delta G_{np}$ | *std* |
|---|---|---|---|---|---|---|---|---|---|---|---|
| 177 | ZINC000044908085 | -38.2 | 3.4 | -51.1 | 2.7 | -21.0 | 3.4 | 37.9 | 2.8 | -4.0 | 0.1 |
| 178 | ZINC000022241454 | -38.1 | 3.8 | -39.1 | 2.9 | -39.2 | 6.6 | 43.6 | 5.3 | -3.4 | 0.1 |
| 179 | ZINC000253407066 | -38.1 | 3.1 | -46.7 | 2.4 | -13.2 | 3.4 | 26.0 | 2.6 | -4.2 | 0.1 |
| 180 | ZINC000057526426 | -38.1 | 2.9 | -44.4 | 2.2 | -28.2 | 3.1 | 38.5 | 2.8 | -4.0 | 0.1 |
| 181 | ZINC000009318781 | -38.1 | 3.9 | -46.3 | 2.8 | -25.5 | 6.6 | 37.5 | 4.4 | -3.9 | 0.1 |
| 182 | ZINC000040548686 | -38.0 | 2.7 | -51.5 | 2.2 | -22.0 | 4.2 | 39.7 | 2.8 | -4.2 | 0.1 |
| 183 | ZINC000072235028 | -38.0 | 2.8 | -52.8 | 2.4 | -12.0 | 4.3 | 31.2 | 3.0 | -4.4 | 0.1 |
| 184 | ZINC000096122792 | -38.0 | 2.8 | -48.6 | 2.6 | -9.0 | 2.7 | 23.6 | 2.8 | -3.9 | 0.1 |
| 185 | ZINC000028790813 | -37.9 | 3.7 | -51.7 | 2.7 | -23.6 | 5.6 | 41.5 | 3.9 | -4.1 | 0.1 |
| 186 | ZINC000239762559 | -37.8 | 3.4 | -48.8 | 2.5 | -20.7 | 4.1 | 35.7 | 3.2 | -4.0 | 0.1 |
| 187 | ZINC000029134571 | -37.7 | 2.6 | -52.1 | 2.4 | -19.8 | 3.2 | 38.5 | 3.1 | -4.3 | 0.1 |
| 188 | ZINC000089857747 | -37.7 | 3.2 | -50.7 | 4.5 | -7.1 | 5.1 | 24.3 | 6.6 | -4.1 | 0.2 |
| 189 | ZINC000048249797 | -37.7 | 3.1 | -50.8 | 2.7 | -10.4 | 5.2 | 27.8 | 2.9 | -4.3 | 0.1 |
| 190 | ZINC000002630701 | -37.7 | 2.6 | -49.0 | 2.0 | -7.6 | 4.1 | 23.0 | 3.7 | -4.0 | 0.1 |
| 191 | ZINC000091487017 | -37.6 | 2.6 | -49.3 | 2.3 | -11.6 | 3.0 | 27.6 | 2.5 | -4.3 | 0.1 |
| 192 | ZINC000021835963 | -37.6 | 2.8 | -48.7 | 2.2 | -25.8 | 3.3 | 40.9 | 2.2 | -4.0 | 0.1 |
| 193 | ZINC000153353600 | -37.6 | 4.2 | -46.2 | 2.7 | -9.4 | 3.9 | 22.0 | 2.4 | -3.9 | 0.1 |
| 194 | ZINC000019327176 | -37.6 | 3.8 | -40.2 | 3.0 | -26.9 | 5.7 | 33.0 | 3.6 | -3.6 | 0.1 |
| 195 | ZINC000005303591 | -37.6 | 2.9 | -44.2 | 2.5 | -20.1 | 3.9 | 30.6 | 2.1 | -3.9 | 0.1 |
| 196 | ZINC000245171655 | -37.5 | 2.7 | -48.7 | 2.3 | -10.2 | 2.7 | 25.4 | 2.0 | -4.0 | 0.1 |
| 197 | ZINC000050857093 | -37.5 | 4.3 | -48.7 | 2.5 | -24.6 | 3.8 | 40.1 | 3.2 | -4.3 | 0.1 |
| 198 | ZINC000004495846 | -37.5 | 2.8 | -46.3 | 2.7 | -25.8 | 4.2 | 38.6 | 4.0 | -4.0 | 0.1 |
| 199 | ZINC000068269627 | -37.5 | 2.8 | -50.4 | 2.4 | -26.9 | 4.4 | 44.0 | 3.8 | -4.1 | 0.1 |
| 200 | ZINC000074014922 | -37.5 | 2.4 | -47.0 | 2.1 | -10.1 | 2.3 | 23.5 | 2.4 | -4.0 | 0.1 |
| 201 | ZINC000058099587 | -37.3 | 3.1 | -46.8 | 2.8 | -18.8 | 3.4 | 32.1 | 2.5 | -3.7 | 0.1 |
| 202 | ZINC000091321614 | -37.3 | 3.1 | -49.4 | 2.4 | -18.1 | 3.8 | 34.5 | 3.7 | -4.2 | 0.1 |
| 203 | ZINC000095447759 | -37.2 | 3.5 | -46.9 | 2.9 | -24.7 | 5.4 | 38.4 | 3.8 | -4.0 | 0.1 |
| 204 | ZINC000072437791 | -37.1 | 2.2 | -46.5 | 2.2 | -14.0 | 3.0 | 27.5 | 2.1 | -4.1 | 0.1 |
| 205 | ZINC000238831563 | -37.1 | 4.0 | -47.8 | 2.5 | -11.5 | 4.4 | 26.4 | 2.4 | -4.2 | 0.1 |
| 206 | ZINC000584897275 | -37.0 | 2.9 | -44.7 | 2.8 | -36.2 | 4.3 | 47.8 | 3.2 | -4.0 | 0.1 |
| 207 | ZINC000077353582 | -36.9 | 3.9 | -48.6 | 2.8 | -15.7 | 3.0 | 31.2 | 2.1 | -3.9 | 0.1 |
| 208 | ZINC000004807849 | -36.9 | 3.1 | -36.5 | 3.5 | -47.7 | 6.0 | 50.9 | 3.7 | -3.7 | 0.2 |
| 209 | ZINC000284798034 | -36.9 | 3.4 | -50.9 | 2.3 | -22.1 | 4.5 | 40.3 | 3.1 | -4.2 | 0.1 |
| 210 | ZINC000062639858 | -36.8 | 2.5 | -52.0 | 2.2 | -3.7 | 3.5 | 23.3 | 3.2 | -4.4 | 0.2 |
| 211 | ZINC000012142779 | -36.8 | 4.7 | -50.2 | 2.6 | -15.4 | 9.7 | 33.2 | 6.6 | -4.4 | 0.1 |
| 212 | ZINC000192929211 | -36.7 | 3.4 | -44.2 | 2.7 | -50.4 | 8.5 | 62.0 | 6.9 | -4.0 | 0.1 |
| 213 | ZINC000097105546 | -36.7 | 2.8 | -44.7 | 2.6 | -21.9 | 3.7 | 33.7 | 3.6 | -3.9 | 0.1 |
| 214 | ZINC000136345745 | -36.7 | 2.8 | -51.8 | 2.4 | -18.5 | 4.8 | 38.0 | 4.4 | -4.3 | 0.1 |
| 215 | ZINC000097050589 | -36.7 | 3.3 | -48.8 | 2.6 | -13.0 | 4.7 | 29.0 | 3.5 | -3.9 | 0.1 |
| 216 | ZINC000040386098 | -36.6 | 2.9 | -42.7 | 2.5 | -14.7 | 3.4 | 24.4 | 2.7 | -3.6 | 0.1 |
| 217 | ZINC000299793701 | -36.6 | 2.9 | -42.4 | 2.8 | -25.9 | 5.0 | 35.5 | 3.4 | -3.8 | 0.1 |
| 218 | ZINC000119429896 | -36.5 | 2.6 | -49.5 | 2.2 | -11.7 | 2.9 | 29.0 | 2.4 | -4.4 | 0.1 |
| 219 | ZINC000035671101 | -36.5 | 2.2 | -46.7 | 2.0 | -8.1 | 1.6 | 21.8 | 1.7 | -3.6 | 0.1 |
| 220 | ZINC000018240833 | -36.5 | 3.4 | -47.5 | 2.8 | -36.3 | 5.3 | 51.6 | 3.3 | -4.3 | 0.1 |
| 221 | ZINC000306138347 | -36.3 | 2.3 | -49.7 | 2.0 | -17.4 | 3.2 | 34.8 | 2.9 | -4.1 | 0.1 |
| 222 | ZINC000061067795 | -36.3 | 2.6 | -48.3 | 2.2 | -15.0 | 3.9 | 31.4 | 2.8 | -4.3 | 0.1 |

| # | ZINC ID | $\Delta G_{bind}$ | *std* | $\Delta E_{vdW}$ | *std* | $\Delta E_{el}$ | *std* | $\Delta G_{pol}$ | *std* | $\Delta G_{np}$ | *std* |
|---|---|---|---|---|---|---|---|---|---|---|---|
| 223 | ZINC000095469884 | -36.2 | 2.9 | -40.0 | 2.8 | -35.7 | 3.7 | 42.9 | 1.9 | -3.4 | 0.1 |
| 224 | ZINC000004535480 | -36.1 | 2.7 | -45.6 | 2.2 | -15.5 | 4.7 | 28.5 | 4.2 | -3.5 | 0.1 |
| 225 | ZINC000096837115 | -36.1 | 6.1 | -45.6 | 4.0 | -26.8 | 7.8 | 40.3 | 5.2 | -4.0 | 0.2 |
| 226 | ZINC000008771448 | -36.0 | 3.9 | -48.5 | 3.0 | -22.3 | 5.8 | 38.9 | 4.9 | -4.1 | 0.3 |
| 227 | ZINC000040385901 | -36.0 | 3.2 | -41.1 | 2.8 | -13.7 | 2.9 | 22.3 | 2.4 | -3.4 | 0.1 |
| 228 | ZINC000008776545 | -35.8 | 2.6 | -47.3 | 2.4 | -15.6 | 2.9 | 31.3 | 2.2 | -4.1 | 0.1 |
| 229 | ZINC000023764785 | -35.7 | 3.3 | -45.9 | 2.6 | -14.8 | 4.5 | 29.0 | 3.2 | -4.1 | 0.1 |
| 230 | ZINC000003947438 | -35.7 | 3.4 | -41.0 | 2.4 | -48.7 | 9.2 | 57.7 | 7.8 | -3.7 | 0.1 |
| 231 | ZINC000192753414 | -35.6 | 3.6 | -46.9 | 2.7 | -33.1 | 4.6 | 48.2 | 2.4 | -3.9 | 0.1 |
| 232 | ZINC000095989182 | -35.6 | 2.8 | -43.2 | 2.2 | -19.0 | 3.6 | 30.3 | 2.6 | -3.8 | 0.1 |
| 233 | ZINC000006669352 | -35.5 | 2.7 | -47.6 | 2.2 | -23.6 | 5.5 | 40.1 | 4.7 | -4.4 | 0.1 |
| 234 | ZINC000253473237 | -35.5 | 2.4 | -44.9 | 2.2 | -8.2 | 2.9 | 21.8 | 1.7 | -4.1 | 0.1 |
| 235 | ZINC000072807915 | -35.5 | 2.7 | -50.6 | 2.3 | -8.1 | 3.6 | 27.4 | 3.4 | -4.2 | 0.1 |
| 236 | ZINC000016228943 | -35.5 | 2.6 | -43.6 | 2.6 | -26.4 | 3.3 | 38.3 | 2.4 | -3.7 | 0.1 |
| 237 | ZINC000077522487 | -35.4 | 2.6 | -48.6 | 2.2 | -27.2 | 5.2 | 44.4 | 4.5 | -4.1 | 0.1 |
| 238 | ZINC000177842371 | -35.3 | 2.6 | -45.3 | 2.0 | -26.9 | 3.7 | 40.5 | 3.0 | -3.7 | 0.1 |
| 239 | ZINC000178511392 | -35.2 | 2.8 | -40.2 | 2.5 | -33.4 | 4.1 | 42.1 | 2.6 | -3.6 | 0.1 |
| 240 | ZINC000005244779 | -35.1 | 2.5 | -49.8 | 2.4 | -9.1 | 2.7 | 28.0 | 2.5 | -4.2 | 0.1 |
| 241 | ZINC000006782318 | -35.1 | 2.6 | -47.7 | 2.3 | -17.2 | 2.7 | 33.7 | 2.4 | -4.0 | 0.1 |
| 242 | ZINC000042025678 | -35.1 | 3.0 | -43.6 | 4.1 | -13.2 | 5.4 | 25.4 | 2.3 | -3.6 | 0.2 |
| 243 | ZINC000012239509 | -35.0 | 3.7 | -50.5 | 2.5 | -8.0 | 5.0 | 27.7 | 2.6 | -4.2 | 0.1 |
| 244 | ZINC000057771728 | -34.9 | 3.3 | -48.2 | 2.5 | -23.3 | 5.5 | 40.9 | 3.6 | -4.3 | 0.2 |
| 245 | ZINC000000079035 | -34.8 | 3.6 | -47.2 | 2.3 | -20.0 | 5.4 | 36.4 | 3.4 | -4.0 | 0.1 |
| 246 | ZINC000075140034 | -34.7 | 2.6 | -41.8 | 2.4 | -19.7 | 2.9 | 30.9 | 2.3 | -4.0 | 0.1 |
| 247 | ZINC000095951555 | -34.6 | 2.8 | -42.9 | 2.3 | -19.4 | 3.3 | 31.4 | 2.2 | -3.8 | 0.1 |
| 248 | ZINC000065591272 | -34.4 | 2.4 | -44.9 | 2.2 | -10.8 | 2.9 | 25.4 | 2.1 | -4.2 | 0.1 |
| 249 | ZINC000257257831 | -34.4 | 3.2 | -45.8 | 2.2 | -13.1 | 4.8 | 28.4 | 3.7 | -3.9 | 0.1 |
| 250 | ZINC000091943530 | -34.4 | 2.9 | -44.1 | 2.7 | -17.5 | 4.4 | 31.0 | 2.9 | -3.8 | 0.1 |
| 251 | ZINC000016543876 | -34.4 | 3.4 | -46.0 | 2.3 | -18.4 | 3.8 | 34.1 | 2.4 | -4.1 | 0.1 |
| 252 | ZINC000030875068 | -34.3 | 2.6 | -45.9 | 2.2 | -11.0 | 3.9 | 26.5 | 2.7 | -3.9 | 0.1 |
| 253 | ZINC000096038482 | -34.2 | 3.7 | -39.5 | 2.9 | -25.3 | 6.7 | 34.3 | 4.0 | -3.7 | 0.1 |
| 254 | ZINC000040266307 | -34.2 | 3.1 | -44.5 | 2.5 | -22.5 | 4.2 | 37.0 | 2.6 | -4.2 | 0.1 |
| 255 | ZINC000084563579 | -34.0 | 3.5 | -45.4 | 4.0 | -21.3 | 15.9 | 36.6 | 13.5 | -4.0 | 0.1 |
| 256 | ZINC000253433605 | -34.0 | 3.8 | -47.3 | 2.5 | -14.9 | 7.6 | 32.2 | 5.8 | -4.1 | 0.1 |
| 257 | ZINC000585093122 | -33.9 | 2.5 | -45.2 | 2.6 | -16.9 | 4.4 | 32.1 | 3.1 | -3.9 | 0.1 |
| 258 | ZINC000019796838 | -33.9 | 3.3 | -46.3 | 2.1 | -10.9 | 3.4 | 27.0 | 2.8 | -3.8 | 0.1 |
| 259 | ZINC000008609997 | -33.9 | 3.3 | -50.6 | 2.4 | -12.7 | 3.4 | 33.3 | 2.7 | -3.9 | 0.1 |
| 260 | ZINC000257311417 | -33.7 | 2.9 | -40.7 | 2.2 | -22.2 | 4.6 | 33.0 | 3.5 | -3.8 | 0.1 |
| 261 | ZINC000255187536 | -33.6 | 2.8 | -42.3 | 2.5 | -21.5 | 3.7 | 34.1 | 2.4 | -3.9 | 0.1 |
| 262 | ZINC000237955843 | -33.5 | 4.0 | -40.6 | 2.6 | -64.6 | 10.7 | 75.6 | 9.4 | -3.9 | 0.2 |
| 263 | ZINC000000326909 | -33.4 | 2.7 | -42.9 | 2.1 | -16.5 | 3.4 | 29.3 | 2.1 | -3.3 | 0.1 |
| 264 | ZINC000034407910 | -33.4 | 2.1 | -38.4 | 2.1 | -16.2 | 2.9 | 24.7 | 2.0 | -3.4 | 0.1 |
| 265 | ZINC000003313076 | -33.4 | 2.9 | -41.7 | 3.0 | -21.4 | 4.5 | 33.6 | 2.2 | -3.8 | 0.1 |
| 266 | ZINC000001381807 | -33.2 | 2.1 | -37.1 | 2.1 | -15.1 | 2.3 | 22.5 | 1.8 | -3.5 | 0.1 |
| 267 | ZINC000006117573 | -33.1 | 2.5 | -42.2 | 2.4 | -29.4 | 2.9 | 42.3 | 2.3 | -3.8 | 0.1 |
| 268 | ZINC000057511709 | -33.0 | 2.8 | -45.5 | 2.0 | -21.3 | 5.0 | 37.6 | 4.6 | -3.8 | 0.1 |

| # | ZINC ID | Δ$G_{bind}$ | *std* | Δ$E_{vdW}$ | *std* | Δ$E_{el}$ | *std* | Δ$G_{pol}$ | *std* | Δ$G_{np}$ | *std* |
|---|---|---|---|---|---|---|---|---|---|---|---|
| 269 | ZINC000072432742 | -33.0 | 2.9 | -49.1 | 2.3 | -23.0 | 9.9 | 43.4 | 9.5 | -4.3 | 0.1 |
| 270 | ZINC000018177963 | -33.0 | 2.7 | -32.7 | 3.1 | -55.0 | 5.3 | 58.3 | 3.2 | -3.6 | 0.1 |
| 271 | ZINC000072234903 | -32.5 | 3.0 | -44.1 | 2.2 | -12.2 | 3.9 | 27.9 | 3.9 | -4.0 | 0.1 |
| 272 | ZINC000001810957 | -32.5 | 2.5 | -45.8 | 2.6 | -17.6 | 3.6 | 35.0 | 2.9 | -4.1 | 0.1 |
| 273 | ZINC000215185695 | -32.3 | 2.4 | -47.4 | 2.1 | -15.8 | 2.1 | 35.3 | 1.9 | -4.4 | 0.1 |
| 274 | ZINC000047209496 | -32.2 | 3.4 | -44.4 | 2.4 | -12.9 | 5.2 | 29.2 | 6.0 | -4.0 | 0.1 |
| 275 | ZINC000002390471 | -32.2 | 2.3 | -43.9 | 2.0 | -12.0 | 2.3 | 27.6 | 2.1 | -3.8 | 0.1 |
| 276 | ZINC000268943077 | -32.1 | 2.7 | -41.8 | 2.6 | -25.1 | 8.3 | 38.5 | 8.3 | -3.7 | 0.1 |
| 277 | ZINC000019973951 | -32.0 | 4.5 | -40.3 | 3.0 | -29.7 | 6.5 | 41.6 | 3.2 | -3.5 | 0.2 |
| 278 | ZINC000002837442 | -31.8 | 2.9 | -42.9 | 2.8 | -16.2 | 2.5 | 31.4 | 1.7 | -4.0 | 0.1 |
| 279 | ZINC000012407997 | -31.8 | 3.3 | -48.0 | 2.8 | -11.5 | 5.7 | 32.0 | 4.9 | -4.2 | 0.2 |
| 280 | ZINC000012508838 | -31.7 | 3.0 | -49.0 | 2.3 | -24.7 | 4.2 | 46.3 | 3.3 | -4.3 | 0.1 |
| 281 | ZINC000004806959 | -31.7 | 2.8 | -39.4 | 2.6 | -55.4 | 9.5 | 67.1 | 8.3 | -4.0 | 0.1 |
| 282 | ZINC000575617533 | -31.6 | 2.4 | -44.8 | 2.3 | -10.6 | 3.0 | 27.7 | 3.2 | -3.9 | 0.2 |
| 283 | ZINC000072277544 | -31.6 | 3.8 | -45.7 | 2.8 | -14.8 | 5.4 | 32.9 | 4.0 | -3.9 | 0.1 |
| 284 | ZINC000237740654 | -31.6 | 3.2 | -45.0 | 2.8 | -28.0 | 10.4 | 45.5 | 10.2 | -4.0 | 0.2 |
| 285 | ZINC000012122382 | -31.4 | 2.8 | -42.6 | 2.3 | -9.5 | 4.8 | 24.4 | 3.2 | -3.8 | 0.2 |
| 286 | ZINC000001620500 | -31.1 | 6.8 | -42.1 | 2.8 | -51.7 | 24.4 | 66.5 | 18.4 | -3.8 | 0.1 |
| 287 | ZINC000069740630 | -30.6 | 4.8 | -38.0 | 2.6 | -20.8 | 4.5 | 31.8 | 2.5 | -3.7 | 0.2 |
| 288 | ZINC000004722011 | -30.6 | 2.6 | -44.0 | 2.2 | -7.3 | 2.7 | 24.4 | 2.4 | -3.6 | 0.1 |
| 289 | ZINC000005088834 | -30.3 | 3.1 | -42.9 | 3.6 | -8.5 | 2.5 | 25.0 | 2.0 | -3.8 | 0.1 |
| 290 | ZINC000027755553 | -30.2 | 2.7 | -46.7 | 2.1 | -15.9 | 3.0 | 36.2 | 2.4 | -3.8 | 0.1 |
| 291 | ZINC000170016387 | -29.8 | 2.7 | -42.6 | 2.8 | -20.5 | 5.3 | 37.3 | 4.4 | -4.0 | 0.1 |
| 292 | ZINC000012557330 | -29.6 | 2.7 | -43.0 | 2.5 | -10.2 | 4.3 | 27.1 | 3.4 | -3.6 | 0.2 |
| 293 | ZINC000004732939 | -29.5 | 3.0 | -43.8 | 2.2 | -13.6 | 3.0 | 31.7 | 2.8 | -3.8 | 0.1 |
| 294 | ZINC000006556210 | -29.3 | 2.5 | -41.4 | 2.6 | -10.2 | 2.5 | 25.9 | 3.2 | -3.6 | 0.1 |
| 295 | ZINC000003852421 | -29.3 | 2.1 | -44.3 | 2.1 | -12.6 | 2.6 | 31.2 | 2.2 | -3.7 | 0.1 |
| 296 | ZINC000017888288 | -29.3 | 3.1 | -44.1 | 2.3 | -16.9 | 4.4 | 35.3 | 3.0 | -3.6 | 0.1 |
| 297 | ZINC000096198984 | -28.3 | 3.3 | -45.9 | 2.9 | -8.7 | 3.7 | 30.4 | 3.7 | -4.1 | 0.2 |
| 298 | ZINC000085879141 | -28.3 | 2.5 | -41.7 | 2.4 | -10.0 | 5.3 | 27.0 | 3.8 | -3.5 | 0.1 |
| 299 | ZINC000006918427 | -28.1 | 3.6 | -45.0 | 2.5 | -13.1 | 4.0 | 33.9 | 2.8 | -3.9 | 0.2 |
| 300 | ZINC000000353960 | -27.6 | 4.1 | -35.6 | 2.7 | -63.0 | 13.2 | 74.4 | 10.6 | -3.4 | 0.2 |
| 301 | ZINC000000201272 | -26.5 | 3.2 | -46.5 | 2.2 | -6.7 | 5.2 | 30.6 | 4.1 | -4.0 | 0.1 |
| 302 | ZINC000237699092 | -26.3 | 3.7 | -40.8 | 3.4 | -32.2 | 15.3 | 50.2 | 14.6 | -3.6 | 0.2 |
| 303 | ZINC000069895275 | -24.7 | 3.8 | -37.0 | 2.2 | -35.4 | 13.7 | 51.2 | 11.2 | -3.5 | 0.1 |
| 304 | ZINC000006576136 | -24.3 | 3.1 | -34.5 | 2.6 | -32.9 | 10.1 | 46.4 | 7.8 | -3.2 | 0.1 |
| 305 | ZINC000008848147 | -21.5 | 3.5 | -38.7 | 3.2 | -26.7 | 14.3 | 47.4 | 11.6 | -3.5 | 0.1 |

**Table S7.** Summary of docking and free energy results obtained for the selected 33 compounds, indicated by compound ID (**Figure S2**), ZINC ID(s) and MolPort ID. The first ZINC ID (*) in multiple sets indicate the enantiomer or diastereomer identified through the virtual screening procedure, whereas the compounds obtained from MolPort were mixtures of the corresponding stereoisomers. The free energy results from MM/PBSA and MM/GBSA calculations are reported for 2 parameter sets employed in the PBSA and GBSA methods. Ranking in boldface (#) indicates highly ranked compound in each parameter set.

| ID | ZINC ID | MolPort ID | VINA score (kcal/mol) | | MM/PB(GB)SA $\Delta G_{bind}$ (kcal/mol) | | | |
|---|---|---|---|---|---|---|---|---|
| | | | *IRAP* | *ERAP1* | *PB-1 (rank)* | *PB-4 (rank)* | *GB-1 (rank)* | *GB-5 (rank)* |
| **1** | 000091486133*<br>000091486131 | MolPort-027-863-667 | -10.2 | -7.5 | -1.3 (#105) | -30.3 (#59) | -51.0 **(#13)** | -50.6 **(#17)** |
| **2** | 000244816835 | MolPort-039-039-060 | -11.0 | -9.2 | -8.1 **(#17)** | -31.5 (#41) | -43.5 (#127) | -42.6 (#95) |
| **3** | 000015078577*<br>000015078575 | MolPort-005-163-536 | -9.9 | -7.1 | -10.8 **(#6)** | -32.6 **(#29)** | -49.0 **(#27)** | -48.7 **(#30)** |
| **4** | 000091270543*<br>000091270544 | MolPort-027-857-260 | -9.9 | -7.4 | -7.8 **(#20)** | -36.9 **(#3)** | -53.3 **(#5)** | -53.9 **(#7)** |
| **5** | 000223692657*<br>000223692703 | MolPort-038-425-524 | -11.0 | -8.9 | -7.5 **(#22)** | -33.5 **(#17)** | -46.0 (#61) | -42.2 (#99) |
| **6** | 000035869910*<br>000035869911<br>000169680566<br>000169680567 | MolPort-006-484-933 | -9.9 | -7.4 | -14.1 **(#2)** | -38.7 **(#1)** | -55.5 **(#2)** | -59.9 **(#2)** |
| **7** | 000008595944 | MolPort-007-773-311 | -10.6 | -8.1 | -5.0 (#42) | -29.1 (#78) | -46.7 (#50) | -52.4 **(#10)** |
| **8** | 000095410774*<br>000095410775<br>000095410776<br>000095410777 | MolPort-027-906-298 | -10.2 | -7.4 | -7.4 **(#23)** | -32.5 **(#31)** | -48.2 (#38) | -47.5 (#40) |
| **9** | 000057563200 | MolPort-035-834-132 | -11.1 | -10.0 | -4.5 (#51) | -35.4 **(#9)** | -50.0 **(#18)** | -46.8 (#48) |
| **10** | 000069562405*<br>000069562406<br>000069562399<br>000069562402 | MolPort-020-152-989 | -10.4 | -7.9 | -9.8 **(#9)** | **-33.7 (#14)** | -46.8 (#48) | -45.0 (#64) |
| **11** | 000051467674*<br>000051467673 | MolPort-019-674-325 | -11.1 | -8.0 | -8.0 **(#19)** | -32.6 **(#30)** | -48.6 **(#31)** | -44.9 (#67) |
| **12** | 000069778215 | MolPort-020-162-189 | -10.9 | -9.5 | -5.0 (#43) | -27.2 (#119) | -53.5 **(#4)** | -56.4 **(#3)** |
| **13** | 000095415912*<br>000095415911<br>000095415910<br>000095415909 | MolPort-028-771-825 | -11.1 | -8.5 | -6.5 **(#27)** | -35.4 **(#8)** | -52.6 **(#9)** | -48.2 (#35) |
| **14** | 000044919578*<br>000044919582 | MolPort-009-543-745 | -10.9 | -8.5 | -8.0 **(#18)** | -29.5 (#72) | -43.2 (#135) | -39.4 (#150) |

| ID | ZINC ID | MolPort ID | VINA score (kcal/mol) | | MM/PB(GB)SA $\Delta G_{bind}$ (kcal/mol) | | | |
|---|---|---|---|---|---|---|---|---|
| | | | *IRAP* | *ERAP1* | *PB-1 (rank)* | *PB-4 (rank)* | *GB-1 (rank)* | *GB-5 (rank)* |
| **15** | 000023642159* 000023642157 | MolPort-004-188-947 | -10.1 | -7.1 | 0.3 (#142) | -26.4 (#137) | -50.5 **(#16)** | -56.4 **(#4)** |
| **16** | 000084598177* 000084598174 | MolPort-027-679-372 | -10.9 | -9.5 | -10.5 **(#8)** | -36.2 **(#6)** | -49.0 **(#26)** | -45.9 (#58) |
| **17** | 000005360885* 000005360891 | MolPort-005-610-771 | -10.9 | -9.6 | -9.7 **(#10)** | -36.0 **(#7)** | -49.0 **(#24)** | -42.6 (#91) |
| **18** | 000043550595 | MolPort-009-519-757 | -10.9 | -8.7 | -5.1 (#41) | -33.1 **(#22)** | -45.9 (#64) | -41.1 (#118) |
| **19** | 000240454699* 000240454698 | MolPort-039-259-003 | -10.9 | -8.6 | -7.0 **(#25)** | -32.2 **(#32)** | -44.3 (#103) | -39.0 (#156) |
| **20** | 000022191233 | MolPort-009-450-517 | -10.9 | -9.2 | -9.2 **(#12)** | -35.1 **(#10)** | -53.1 **(#6)** | -49.4 **(#24)** |
| **21** | 000003463362* 000003463364 000003463365 | MolPort-004-164-037 | -10.8 | -8.1 | -7.3 **(#24)** | -37.7 **(#2)** | -52.6 **(#8)** | -51.5 **(#15)** |
| **22** | 000084578820* 000084578821 000084578815 000084578818 | MolPort-027-704-758 | -10.5 | -8.0 | -13.9 **(#3)** | -36.3 **(#4)** | -51.1 **(#12)** | -48.8 **(#28)** |
| **23** | 000071915224* 000071915223 | MolPort-020-057-841 | -10.9 | -9.2 | -5.8 **(#33)** | -32.9 **(#25)** | -46.2 (#60) | -42.8 (#87) |
| **24** | 000025032259* 000025032263 | MolPort-009-027-892 | -10.7 | -8.1 | -3.0 (#75) | -31.0 (#46) | -49.1 **(#22)** | -45.7 (#63) |
| **25** | 000069737923* 000069737924 | MolPort-020-053-566 | -9.8 | -7.1 | -7.7 **(#21)** | -33.2 **(#20)** | -46.2 (#58) | -42.1 (#101) |
| **26** | 000010256259* 000010256257 | MolPort-003-173-337 | -11.0 | -9.7 | -15.7 **(#1)** | -34.3 **(#13)** | -53.6 **(#3)** | -48.6 **(#31)** |
| **27** | 000000333210* 000000333211 | MolPort-003-801-767 | -10.9 | -9.0 | -8.3 **(#16)** | -33.6 **(#15)** | -49.8 **(#19)** | -46.9 (#45) |
| **28** | 000004998502* 000004998501 | MolPort-000-832-181 | -10.9 | -9.3 | -3.9 (#68) | -30.8 (#53) | -52.8 **(#7)** | -47.0 (#44) |
| **29** | 000008913866* 000008913865 | MolPort-000-828-576 | -10.9 | -8.3 | -4.7 (#50) | -29.3 (#75) | -49.1 **(#23)** | -50.6 **(#19)** |
| **30** | 000018148904* 000100900762 | MolPort-000-694-123 | -10.2 | -7.7 | -9.5 **(#11)** | -33.4 **(#18)** | -48.2 (#37) | -42.1 (#102) |
| **31** | 000079491038 | MolPort-023-297-297 | -10.4 | -7.8 | -10.5 **(#7)** | -34.4 **(#12)** | -48.5 (#32) | -43.5 (#77) |
| **32** | 000004511530 | MolPort-000-407-697 | -10.1 | -7.6 | -8.6 **(#14)** | -28.5 (#92) | -45.2 (#83) | -40.3 (#133) |
| **33** | 000002216953 | MolPort-001-905-390 | -10.3 | -7.7 | -4.4 (#53) | -31.0 (#50) | -49.0 **(#25)** | -46.7 (#49) |

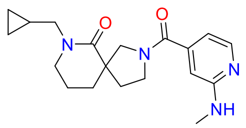

**1**

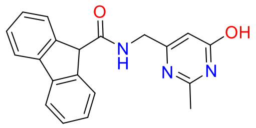

**2**

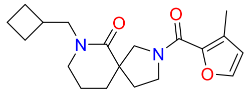

**3**

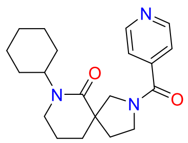

**4**

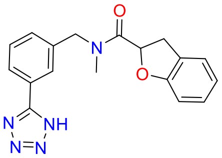

**5**

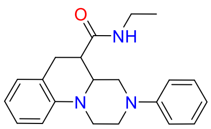

**6**

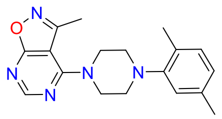

**7**

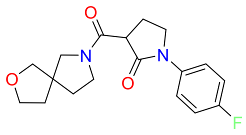

**8**

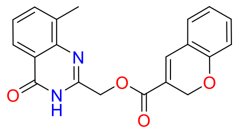

**9**

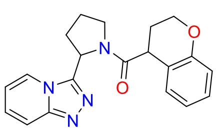

**10**

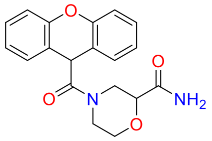

**11**

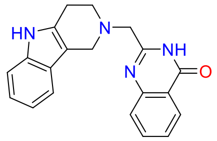

**12**

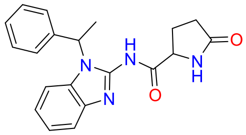

**13**

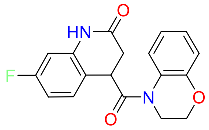

**14**

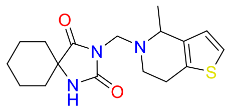

**15**

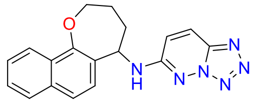

**16**

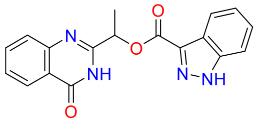

**17**

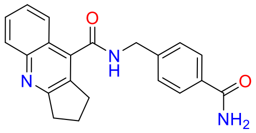

**18**

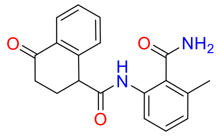

**19**

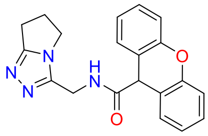

**20**

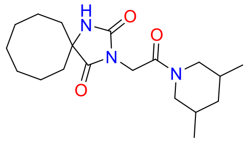

**21**

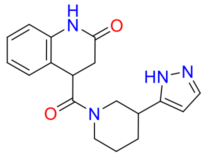

**22**

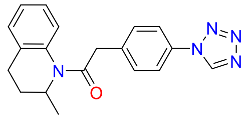

**23**

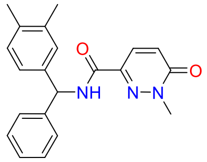

**24**

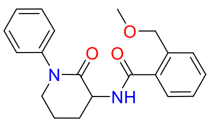

**25**

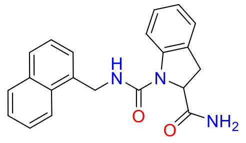

**26**

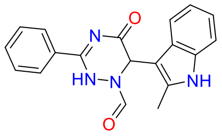

**27**

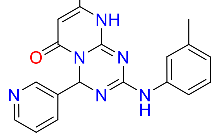

**28**

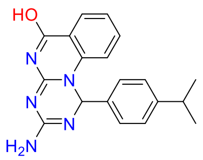

**29**

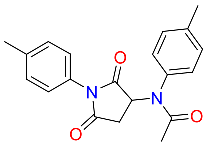

**30**

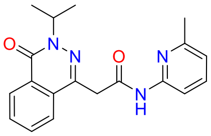

**31**

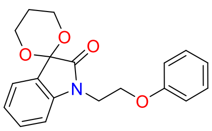

**32**

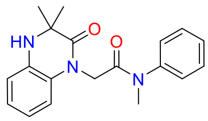

**33**

**Figure S2.** Structures of the 33 compounds selected for evaluation as inhibitors of IRAP. Where applicable, stereochemistry is not defined to indicate that mixture of enantiomers or diastereomers was obtained from MolPort and was used without further purification.

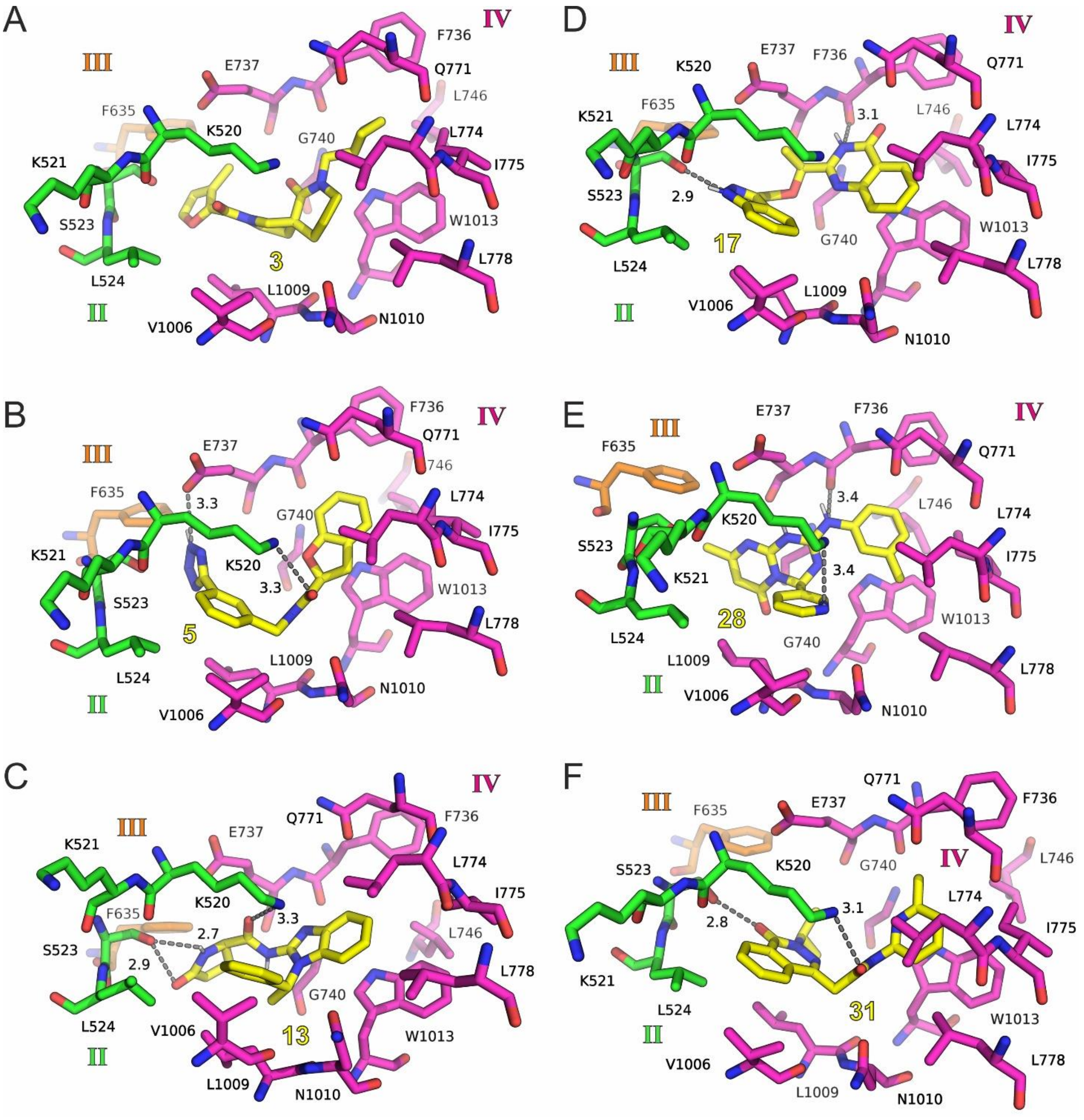


**Figure S3.** Bound conformations of the active compounds **3, 5, 13, 17, 28** and **31** inside the targeted pocket of IRAP. Surrounding residues are color-coded according to IRAP domain (II, III or IV) as indicated. Potential hydrogen-bonding interactions are shown with dashed lines and the corresponding heavy-atom distances (Å).

**Figure S4.** Computed physiochemical descriptors and radar plots as obtained using SwissADME server for compounds **1–33** (including ZINC ID). The colored zone in the radar plots indicates favorable physiochemical space for oral bioavailability, using the following descriptors: Lipophilicity ( –0.7 < xLog*P*3 < 5.0); Size (150 < MW < 500, g/mol); Polarity (20 < TPSA < 130, Å$^2$); Insolubility (0 < Log$S_{ESOL}$ < 6); Insaturation (0.25 < Fsp3 < 1); Flexibility (0 < rotatable torsions < 9). Detailed information about the descriptors can be found in reference [1] given below.

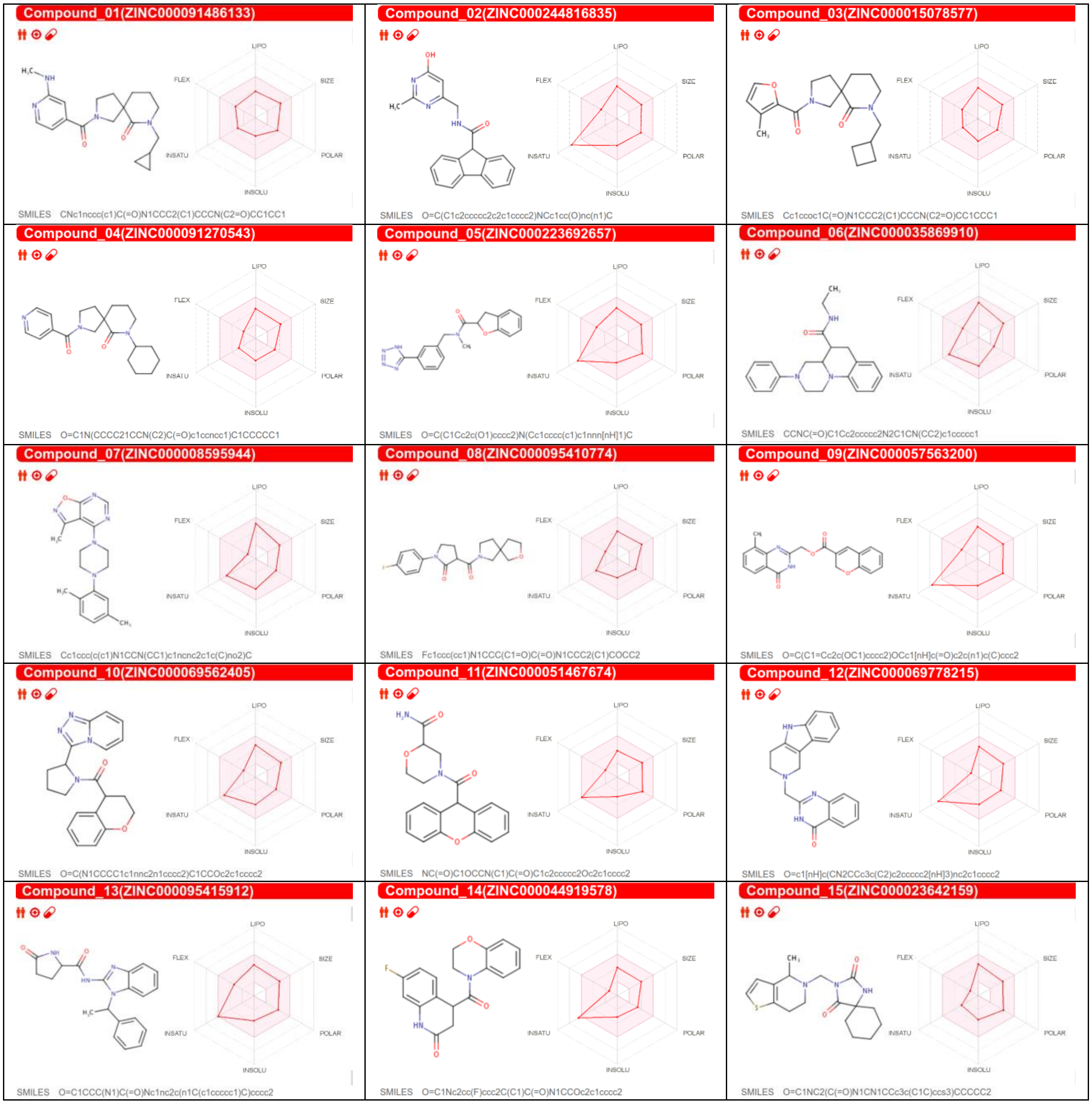


[1] Daina, A., Michielin, O. & Zoete, V. SwissADME: a free web tool to evaluate pharmacokinetics, drug-likeness and medicinal chemistry friendliness of small molecules. Sci Rep 7, 42717 (2017).

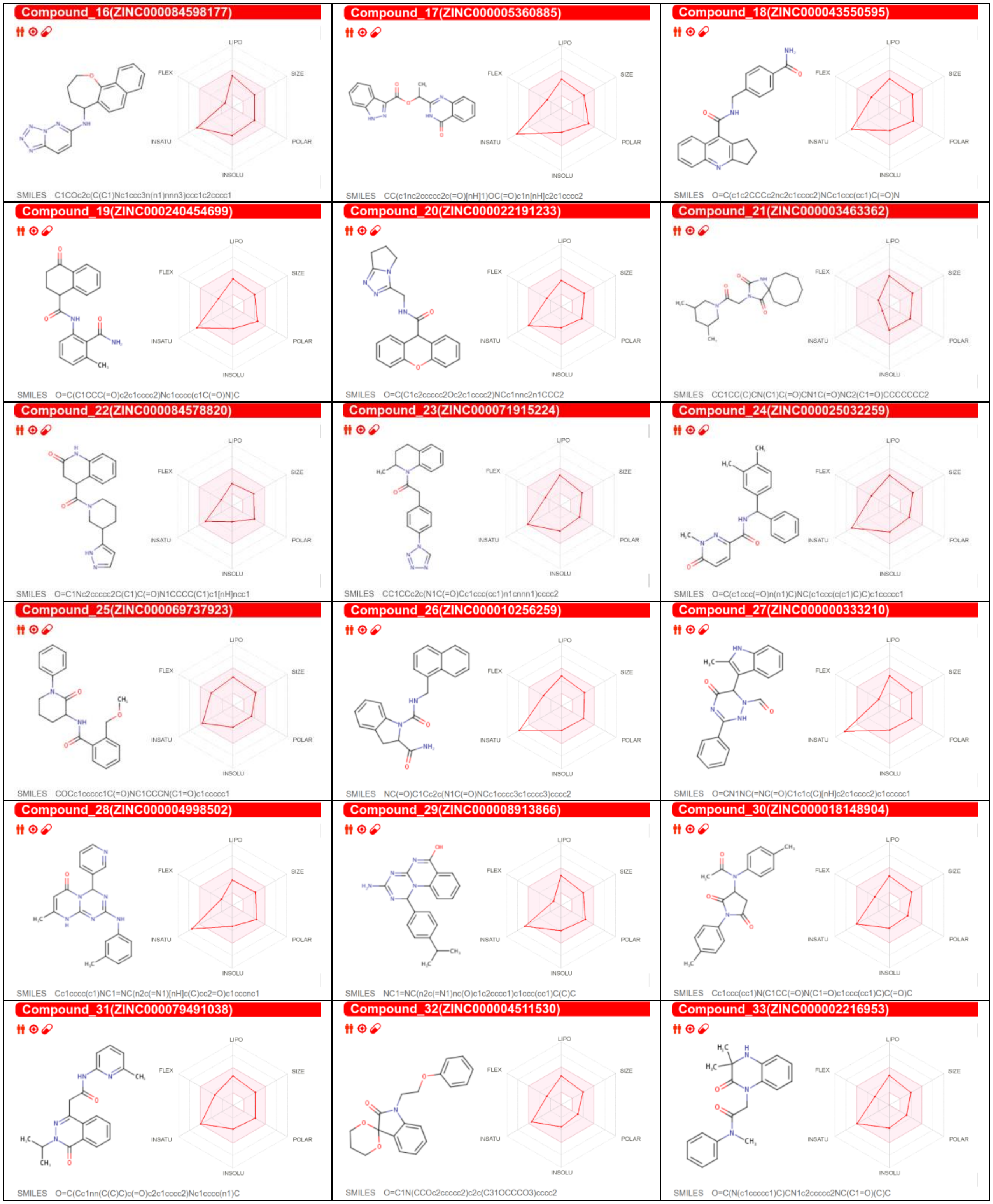


**Table S8.** Computed physiochemical and drug-likeness descriptors, along with selected ADME parameters for compounds **1–33** as calculated using the SwissADME server are given in pp. 47–51. More details about the descriptors can be found in ref. [1] and at http://www.swissadme.ch/

| ID | 1 | 2 | 3 | 4 | 5 | 6 | 7 |
|---|---|---|---|---|---|---|---|
| ZINC ID | ZINC000091486133 | ZINC000244816835 | ZINC000015078577 | ZINC000091270543 | ZINC000223692657 | ZINC000035869910 | ZINC000008595944 |
| Formula | C19H26N4O2 | C20H17N3O2 | C19H26N2O3 | C20H27N3O2 | C18H17N5O2 | C21H25N3O | C18H21N5O |
| MW | 342.4 | 331.4 | 330.4 | 341.5 | 335.4 | 335.4 | 323.4 |
| #Heavy atoms | 25 | 25 | 24 | 25 | 25 | 25 | 24 |
| #Aromatic heavy atoms | 6 | 18 | 5 | 6 | 17 | 12 | 15 |
| Fraction Csp3 | 0.63 | 0.15 | 0.68 | 0.65 | 0.22 | 0.38 | 0.39 |
| #Rotatable bonds | 5 | 4 | 4 | 3 | 5 | 4 | 2 |
| #H-bond acceptors | 3 | 4 | 3 | 3 | 5 | 1 | 4 |
| #H-bond donors | 1 | 2 | 0 | 0 | 1 | 1 | 0 |
| MR | 103.83 | 94.57 | 98.77 | 104.14 | 90.96 | 108.02 | 100.99 |
| TPSA | 65.54 | 75.11 | 53.76 | 53.51 | 84 | 35.58 | 58.29 |
| iLOGP | 2.85 | 2.38 | 3.45 | 2.96 | 2.34 | 2.71 | 3.2 |
| XLOGP3 | 1.28 | 2.72 | 2.34 | 2.03 | 2.27 | 3.39 | 3.5 |
| ESOL Class | Soluble | Soluble | Soluble | Soluble | Soluble | Moderately soluble | Moderately soluble |
| GI absorption | High | High | High | High | High | High | High |
| BBB permeant | No | Yes | Yes | Yes | No | Yes | Yes |
| Pgp substrate | Yes | Yes | Yes | Yes | No | Yes | No |
| CYP1A2 inhibitor | No | No | No | No | No | No | Yes |
| CYP2C19 inhibitor | No | Yes | No | No | Yes | Yes | No |
| CYP2C9 inhibitor | No | Yes | Yes | Yes | Yes | No | Yes |
| CYP2D6 inhibitor | Yes | Yes | Yes | Yes | No | Yes | Yes |
| CYP3A4 inhibitor | No | Yes | Yes | Yes | Yes | Yes | Yes |
| log Kp (cm/s) | -7.48 | -6.39 | -6.65 | -6.94 | -6.73 | -5.94 | -5.79 |
| Lipinski #violations | 0 | 0 | 0 | 0 | 0 | 0 | 0 |
| Ghose #violations | 0 | 0 | 0 | 0 | 0 | 0 | 0 |
| Veber #violations | 0 | 0 | 0 | 0 | 0 | 0 | 0 |
| Egan #violations | 0 | 0 | 0 | 0 | 0 | 0 | 0 |
| Muegge #violations | 0 | 0 | 0 | 0 | 0 | 0 | 0 |
| Bioavailability Score | 0.55 | 0.55 | 0.55 | 0.55 | 0.56 | 0.55 | 0.55 |
| PAINS #alerts | 0 | 0 | 0 | 0 | 0 | 0 | 0 |
| Brenk #alerts | 0 | 0 | 0 | 0 | 0 | 0 | 0 |
| Leadlikeness #violations | 0 | 0 | 0 | 0 | 0 | 0 | 0 |
| Synthetic Accessibility | 3.69 | 3.22 | 3.97 | 3.43 | 3.13 | 3.43 | 3.23 |

| ID | 8 | 9 | 10 | 11 | 12 | 13 | 14 |
|---|---|---|---|---|---|---|---|
| ZINC ID | ZINC000095410774 | ZINC000057563200 | ZINC000069562405 | ZINC000051467674 | ZINC000069778215 | ZINC000095415912 | ZINC000044919578 |
| Formula | C18H21FN2O3 | C20H16N2O4 | C20H20N4O2 | C19H18N2O4 | C20H18N4O | C20H20N4O2 | C18H15FN2O3 |
| MW | 332.4 | 348.4 | 348.4 | 338.4 | 330.4 | 348.4 | 326.3 |
| #Heavy atoms | 24 | 26 | 26 | 25 | 25 | 26 | 24 |
| #Aromatic heavy atoms | 6 | 16 | 15 | 12 | 19 | 15 | 12 |
| Fraction Csp3 | 0.56 | 0.15 | 0.35 | 0.26 | 0.2 | 0.25 | 0.22 |
| #Rotatable bonds | 3 | 4 | 3 | 3 | 2 | 5 | 2 |
| #H-bond acceptors | 4 | 5 | 4 | 4 | 3 | 3 | 4 |
| #H-bond donors | 0 | 1 | 0 | 1 | 2 | 2 | 1 |
| MR | 93.31 | 97.18 | 100.56 | 93.7 | 102.95 | 103.83 | 92.55 |
| TPSA | 49.85 | 81.28 | 59.73 | 81.86 | 64.78 | 76.02 | 58.64 |
| iLOGP | 2.75 | 3.07 | 2.96 | 2.19 | 2.15 | 1.58 | 2.42 |
| XLOGP3 | 1.41 | 2.69 | 2.76 | 1.23 | 2.45 | 2.39 | 1.64 |
| ESOL Class | Soluble | Soluble | Soluble | Soluble | Soluble | Soluble | Soluble |
| GI absorption | High | High | High | High | High | High | High |
| BBB permeant | Yes | No | Yes | No | Yes | Yes | Yes |
| Pgp substrate | Yes | No | No | Yes | Yes | Yes | Yes |
| CYP1A2 inhibitor | No | Yes | No | No | Yes | No | No |
| CYP2C19 inhibitor | No | Yes | Yes | No | No | Yes | Yes |
| CYP2C9 inhibitor | No | Yes | Yes | No | No | No | No |
| CYP2D6 inhibitor | Yes | No | Yes | Yes | Yes | Yes | Yes |
| CYP3A4 inhibitor | Yes | Yes | Yes | No | Yes | Yes | Yes |
| log Kp (cm/s) | -7.33 | -6.52 | -6.47 | -7.49 | -6.58 | -6.73 | -7.13 |
| Lipinski #violations | 0 | 0 | 0 | 0 | 0 | 0 | 0 |
| Ghose #violations | 0 | 0 | 0 | 0 | 0 | 0 | 0 |
| Veber #violations | 0 | 0 | 0 | 0 | 0 | 0 | 0 |
| Egan #violations | 0 | 0 | 0 | 0 | 0 | 0 | 0 |
| Muegge #violations | 0 | 0 | 0 | 0 | 0 | 0 | 0 |
| Bioavailability Score | 0.55 | 0.55 | 0.55 | 0.55 | 0.55 | 0.55 | 0.55 |
| PAINS #alerts | 0 | 0 | 0 | 0 | 1 | 0 | 0 |
| Brenk #alerts | 1 | 0 | 0 | 0 | 0 | 0 | 0 |
| Leadlikeness #violations | 0 | 0 | 0 | 0 | 0 | 0 | 0 |
| Synthetic Accessibility | 3.64 | 3.31 | 3.77 | 3.7 | 2.67 | 3.32 | 3.16 |

| ID | 15 | 16 | 17 | 18 | 19 | 20 | 21 |
|---|---|---|---|---|---|---|---|
| ZINC ID | ZINC000023642159 | ZINC000084598177 | ZINC000005360885 | ZINC000043550595 | ZINC000240454699 | ZINC000022191233 | ZINC000003463362 |
| Formula | C17H23N3O2S | C18H16N6O | C18H14N4O3 | C21H19N3O2 | C19H18N2O3 | C20H18N4O2 | C19H31N3O3 |
| MW | 333.5 | 332.4 | 334.3 | 345.4 | 322.4 | 346.4 | 349.5 |
| #Heavy atoms | 23 | 25 | 25 | 26 | 24 | 26 | 25 |
| #Aromatic heavy atoms | 5 | 19 | 19 | 16 | 12 | 17 | 0 |
| Fraction Csp3 | 0.65 | 0.22 | 0.11 | 0.19 | 0.21 | 0.25 | 0.84 |
| #Rotatable bonds | 2 | 2 | 4 | 5 | 4 | 4 | 3 |
| #H-bond acceptors | 3 | 5 | 5 | 3 | 3 | 4 | 3 |
| #H-bond donors | 1 | 1 | 2 | 2 | 2 | 1 | 1 |
| MR | 101.95 | 93.87 | 92.59 | 99.95 | 91.19 | 95.57 | 108.47 |
| TPSA | 80.89 | 77.23 | 100.73 | 85.08 | 89.26 | 69.04 | 69.72 |
| iLOGP | 2.97 | 2.77 | 1.59 | 2.34 | 1.78 | 2.62 | 3.37 |
| XLOGP3 | 2.52 | 3.43 | 2.64 | 2.52 | 2.36 | 1.83 | 3.07 |
| ESOL Class | Soluble | Moderately soluble | Soluble | Soluble | Soluble | Soluble | Soluble |
| GI absorption | High | High | High | High | High | High | High |
| BBB permeant | No | Yes | No | No | No | Yes | Yes |
| Pgp substrate | Yes | No | No | Yes | Yes | Yes | Yes |
| CYP1A2 inhibitor | No | Yes | No | Yes | No | Yes | No |
| CYP2C19 inhibitor | Yes | Yes | Yes | No | No | Yes | No |
| CYP2C9 inhibitor | Yes | No | No | Yes | No | No | No |
| CYP2D6 inhibitor | Yes | No | No | Yes | No | Yes | No |
| CYP3A4 inhibitor | Yes | No | No | Yes | Yes | Yes | No |
| log Kp (cm/s) | -6.54 | -5.89 | -6.47 | -6.62 | -6.59 | -7.11 | -6.25 |
| Lipinski #violations | 0 | 0 | 0 | 0 | 0 | 0 | 0 |
| Ghose #violations | 0 | 0 | 0 | 0 | 0 | 0 | 0 |
| Veber #violations | 0 | 0 | 0 | 0 | 0 | 0 | 0 |
| Egan #violations | 0 | 0 | 0 | 0 | 0 | 0 | 0 |
| Muegge #violations | 0 | 0 | 0 | 0 | 0 | 0 | 0 |
| Bioavailability Score | 0.55 | 0.55 | 0.55 | 0.55 | 0.55 | 0.55 | 0.55 |
| PAINS #alerts | 0 | 0 | 0 | 0 | 0 | 0 | 0 |
| Brenk #alerts | 1 | 0 | 0 | 0 | 0 | 0 | 1 |
| Leadlikeness #violations | 0 | 0 | 0 | 0 | 0 | 0 | 0 |
| Synthetic Accessibility | 4.17 | 3.59 | 3.16 | 2.61 | 3.05 | 3.65 | 4.19 |

| ID | 22 | 23 | 24 | 25 | 26 | 27 |
|---|---|---|---|---|---|---|
| ZINC ID | ZINC000084578820 | ZINC000071915224 | ZINC000025032259 | ZINC000069737923 | ZINC000010256259 | ZINC000000333210 |
| Formula | C18H20N4O2 | C19H19N5O | C21H21N3O2 | C20H22N2O3 | C21H19N3O2 | C19H16N4O2 |
| MW | 324.4 | 333.4 | 347.4 | 338.4 | 345.4 | 332.4 |
| #Heavy atoms | 24 | 25 | 26 | 25 | 26 | 25 |
| #Aromatic heavy atoms | 11 | 17 | 18 | 12 | 16 | 15 |
| Fraction Csp3 | 0.39 | 0.26 | 0.19 | 0.3 | 0.14 | 0.11 |
| #Rotatable bonds | 3 | 4 | 5 | 6 | 5 | 3 |
| #H-bond acceptors | 3 | 4 | 3 | 3 | 2 | 3 |
| #H-bond donors | 2 | 0 | 1 | 1 | 2 | 2 |
| MR | 96.91 | 98.26 | 101.66 | 99.47 | 104.34 | 106.35 |
| TPSA | 78.09 | 63.91 | 63.99 | 58.64 | 75.43 | 77.56 |
| iLOGP | 1.77 | 2.94 | 3.02 | 2.87 | 2.34 | 1.81 |
| XLOGP3 | 0.8 | 3 | 3.09 | 2.46 | 2.87 | 2.79 |
| ESOL Class | Soluble | Moderately soluble | Moderately soluble | Soluble | Soluble | Soluble |
| GI absorption | High | High | High | High | High | High |
| BBB permeant | No | Yes | Yes | Yes | Yes | No |
| Pgp substrate | Yes | No | No | Yes | Yes | No |
| CYP1A2 inhibitor | No | No | No | No | No | No |
| CYP2C19 inhibitor | No | Yes | Yes | Yes | Yes | Yes |
| CYP2C9 inhibitor | No | Yes | Yes | Yes | Yes | Yes |
| CYP2D6 inhibitor | Yes | No | No | Yes | Yes | No |
| CYP3A4 inhibitor | No | No | Yes | Yes | Yes | No |
| log Kp (cm/s) | -7.71 | -6.2 | -6.23 | -6.62 | -6.37 | -6.35 |
| Lipinski #violations | 0 | 0 | 0 | 0 | 0 | 0 |
| Ghose #violations | 0 | 0 | 0 | 0 | 0 | 0 |
| Veber #violations | 0 | 0 | 0 | 0 | 0 | 0 |
| Egan #violations | 0 | 0 | 0 | 0 | 0 | 0 |
| Muegge #violations | 0 | 0 | 0 | 0 | 0 | 0 |
| Bioavailability Score | 0.55 | 0.55 | 0.55 | 0.55 | 0.55 | 0.55 |
| PAINS #alerts | 0 | 0 | 0 | 0 | 0 | 1 |
| Brenk #alerts | 0 | 0 | 0 | 0 | 0 | 1 |
| Leadlikeness #violations | 0 | 0 | 0 | 0 | 0 | 0 |
| Synthetic Accessibility | 3.4 | 3 | 3.13 | 2.78 | 3.12 | 3.79 |

| ID | 28 | 29 | 30 | 31 | 32 | 33 |
|---|---|---|---|---|---|---|
| ZINC ID | ZINC000004998502 | ZINC000008913866 | ZINC000018148904 | ZINC000079491038 | ZINC000004511530 | ZINC000002216953 |
| Formula | C19H18N6O | C19H19N5O | C20H20N2O3 | C19H20N4O2 | C19H19NO4 | C19H21N3O2 |
| MW | 346.4 | 333.4 | 336.4 | 336.4 | 325.4 | 323.4 |
| #Heavy atoms | 26 | 25 | 25 | 25 | 24 | 24 |
| #Aromatic heavy atoms | 18 | 16 | 12 | 16 | 12 | 12 |
| Fraction Csp3 | 0.16 | 0.21 | 0.25 | 0.26 | 0.32 | 0.26 |
| #Rotatable bonds | 3 | 2 | 4 | 5 | 4 | 4 |
| #H-bond acceptors | 4 | 4 | 3 | 4 | 4 | 2 |
| #H-bond donors | 2 | 2 | 0 | 1 | 0 | 1 |
| MR | 107.01 | 104.44 | 100.15 | 98.44 | 91.82 | 102.3 |
| TPSA | 87.43 | 88.79 | 57.69 | 76.88 | 48 | 52.65 |
| iLOGP | 2.82 | 2.72 | 2.95 | 2.91 | 3.47 | 3.06 |
| XLOGP3 | 1.6 | 2.84 | 2.62 | 2.67 | 2.51 | 2.75 |
| ESOL Class | Soluble | Moderately soluble | Soluble | Soluble | Soluble | Soluble |
| GI absorption | High | High | High | High | High | High |
| BBB permeant | No | No | Yes | Yes | Yes | Yes |
| Pgp substrate | No | No | No | No | Yes | No |
| CYP1A2 inhibitor | No | No | No | No | Yes | Yes |
| CYP2C19 inhibitor | Yes | No | Yes | Yes | No | Yes |
| CYP2C9 inhibitor | No | Yes | Yes | Yes | Yes | Yes |
| CYP2D6 inhibitor | No | No | No | No | Yes | Yes |
| CYP3A4 inhibitor | Yes | No | Yes | No | Yes | Yes |
| log Kp (cm/s) | -7.28 | -6.32 | -6.49 | -6.46 | -6.5 | -6.32 |
| Lipinski #violations | 0 | 0 | 0 | 0 | 0 | 0 |
| Ghose #violations | 0 | 0 | 0 | 0 | 0 | 0 |
| Veber #violations | 0 | 0 | 0 | 0 | 0 | 0 |
| Egan #violations | 0 | 0 | 0 | 0 | 0 | 0 |
| Muegge #violations | 0 | 0 | 0 | 0 | 0 | 0 |
| Bioavailability Score | 0.55 | 0.55 | 0.55 | 0.55 | 0.55 | 0.55 |
| PAINS #alerts | 0 | 0 | 0 | 0 | 0 | 0 |
| Brenk #alerts | 0 | 0 | 1 | 0 | 0 | 0 |
| Leadlikeness #violations | 0 | 0 | 0 | 0 | 0 | 0 |
| Synthetic Accessibility | 4.34 | 4.15 | 2.75 | 2.81 | 3.21 | 2.55 |

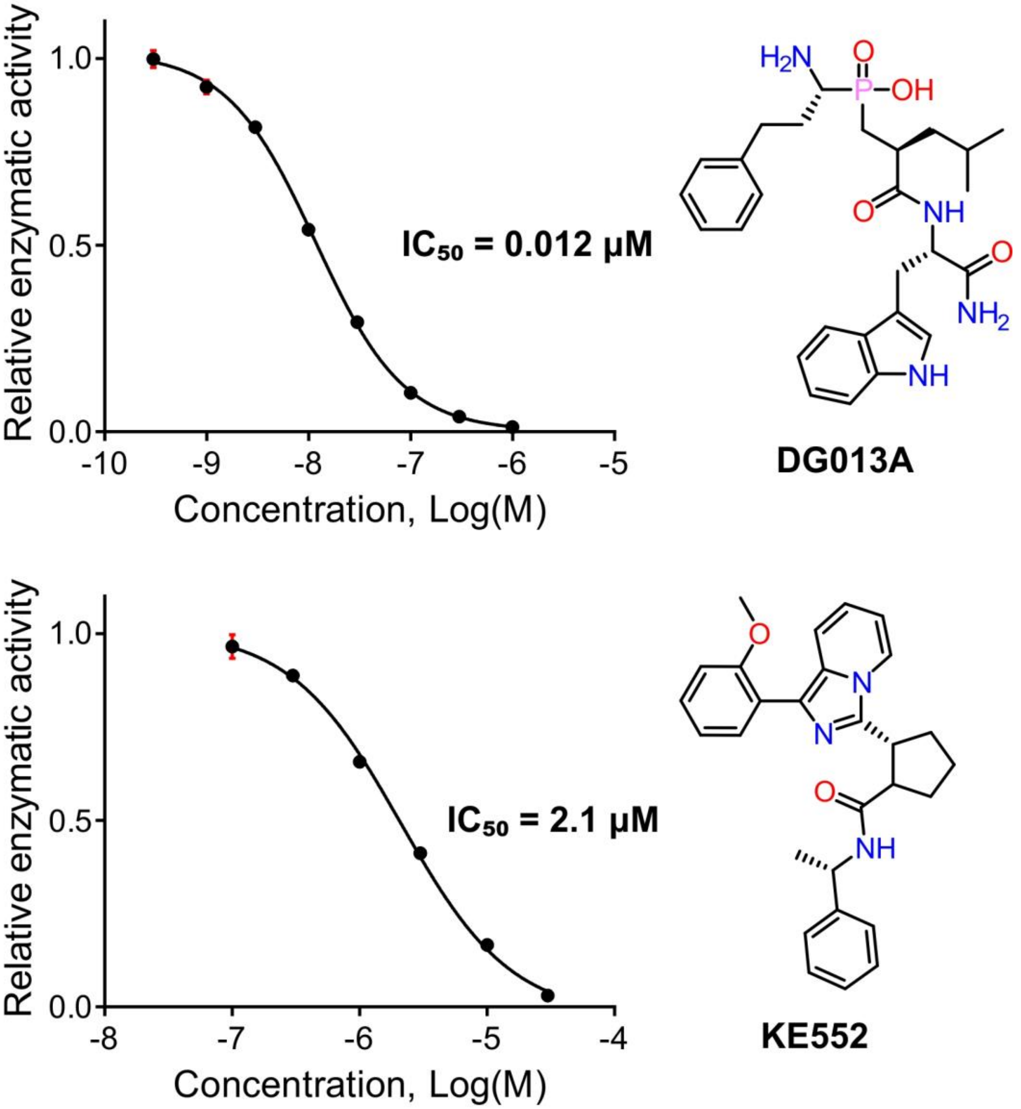


**Figure S5.** Validation of the effect of the known inhibitors **DG013A** and **KE552** on hydrolysis of the small fluorigenic substrate Leu-AMC by IRAP. Error bars indicate standard deviation and are shown only if they are significantly larger than the size of data points.

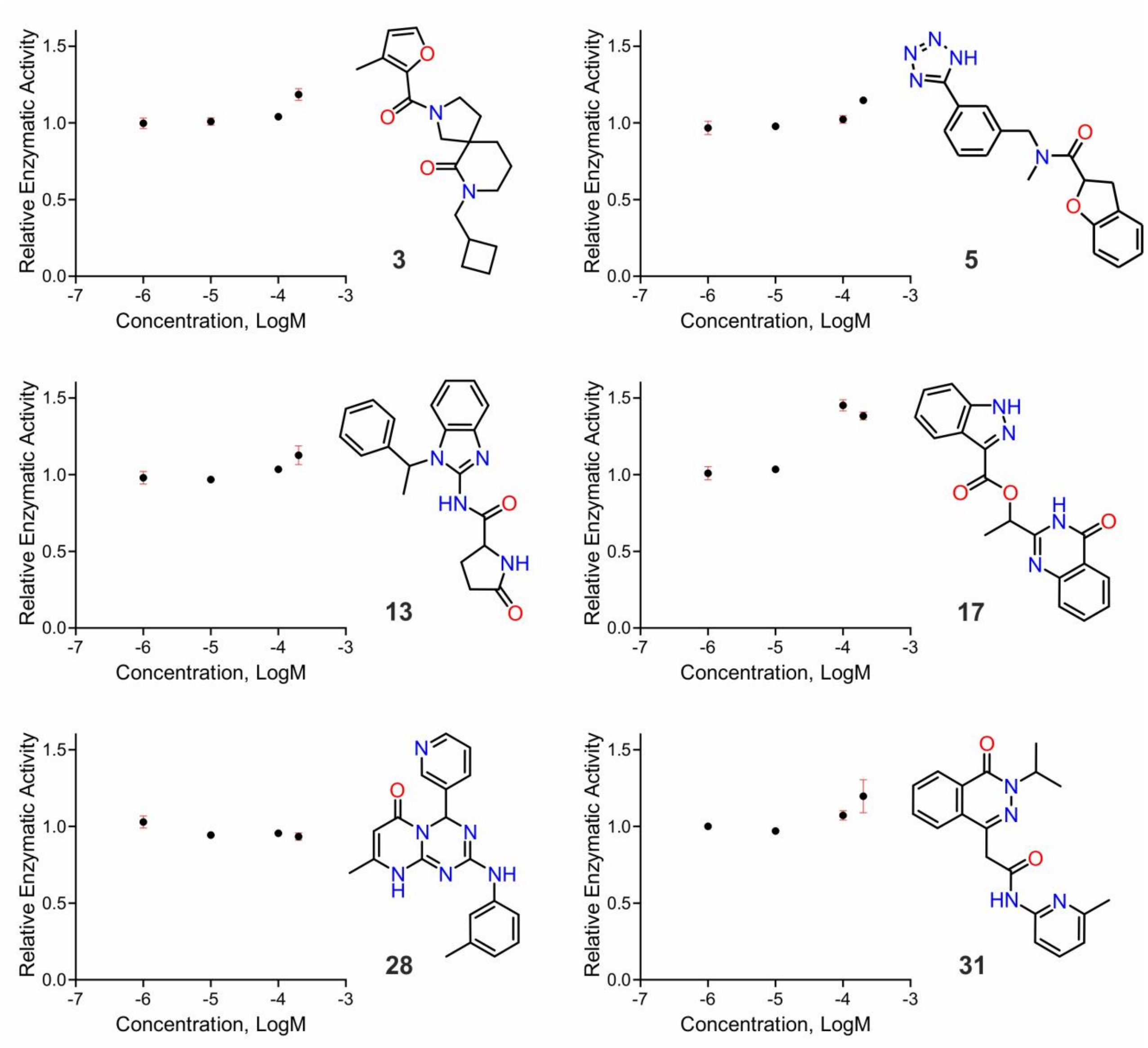


**Figure S6.** Effect of the hit compounds (**3, 5, 13, 17, 28** and **31**) on hydrolysis of the small fluorigenic substrate Leu-AMC by the homologous ER aminopeptidase ERAP1. Error bars indicate standard deviation and are shown only if they are significantly larger than the size of data points.

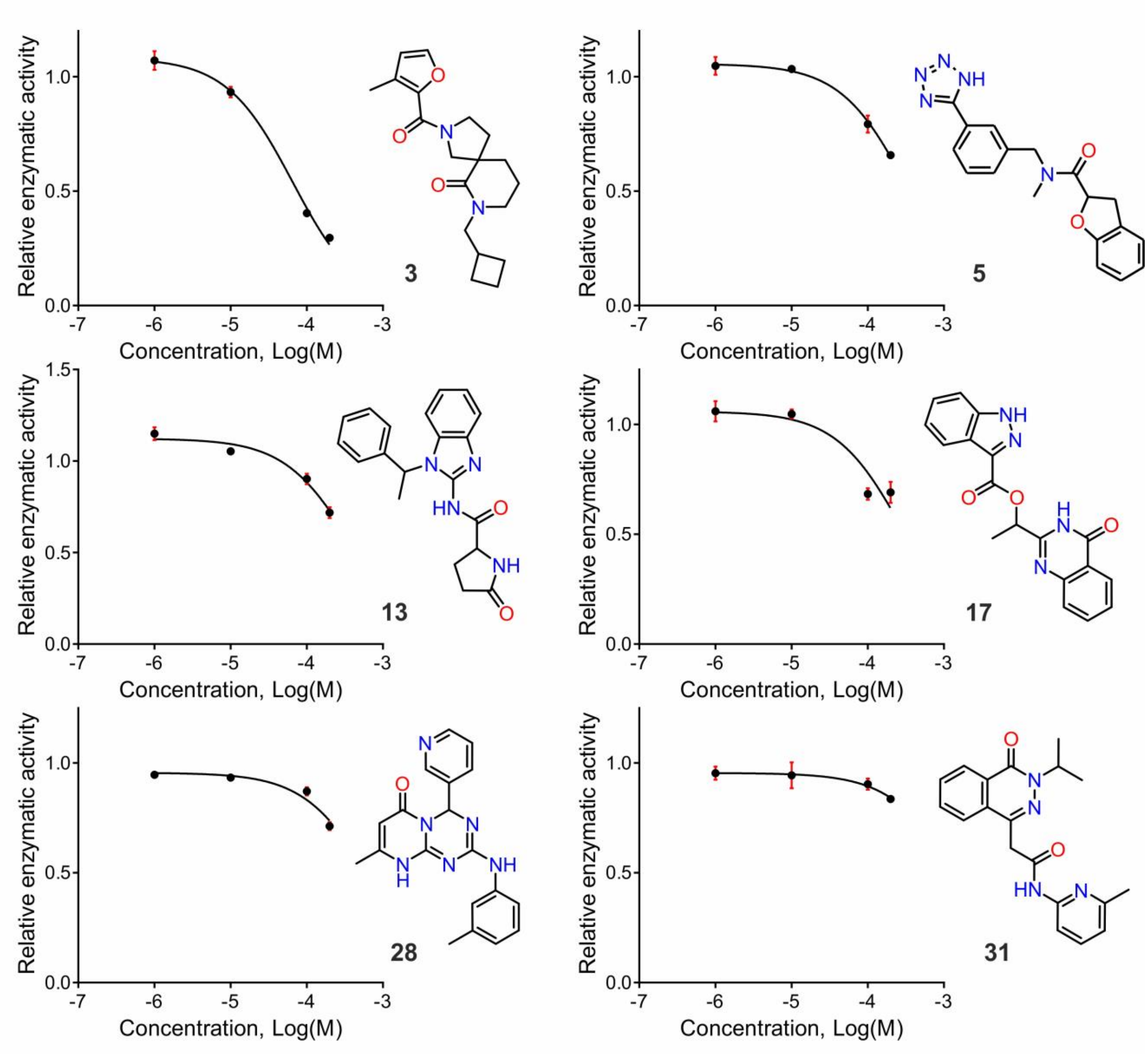


**Figure S7.** Effect of the hit compounds (**3, 5, 13, 17, 28** and **31)** on hydrolysis of the small fluorigenic substrate Arg-AMC by the homologous ER aminopeptidase ERAP2. Error bars indicate standard deviation and are shown only if they are significantly larger than the size of data points.

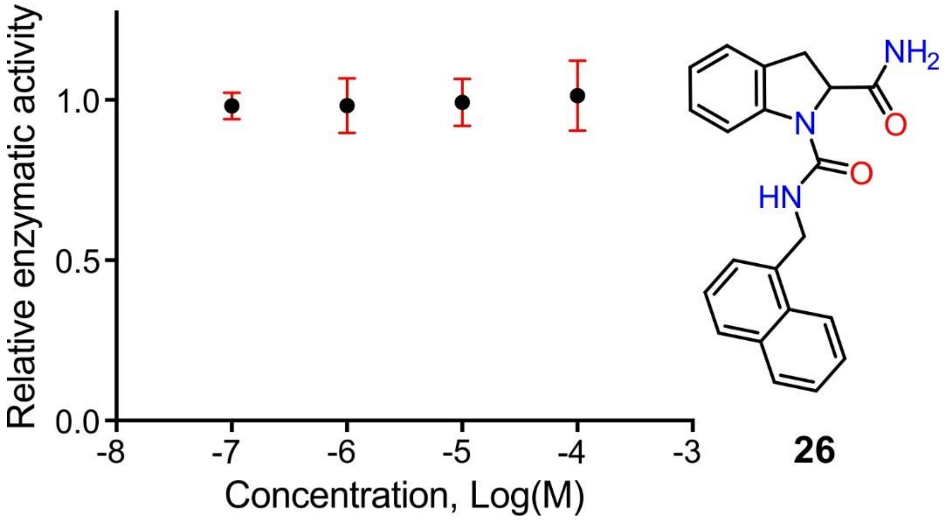

**26**

**Figure S8.** The effect of **26** on hydrolysis of the small fluorigenic substrate Leu-AMC, here at a concentration of 5 μM, by IRAP. Error bars indicate standard deviation.
